# Exploring causal links between autoimmune liver diseases, chronic hepatitis C, and thyroid disorders: Evidence from NHANES and GWAS studies

**DOI:** 10.1097/MD.0000000000044112

**Published:** 2025-08-29

**Authors:** Qilong Nie, Yongwen Jiang, Qiuyan Liang, Mingyang Li, Caiyang Huang, Yongwei Yuan, Tengyu Qiu, Xiaojun Ma, Jianhong Li

**Affiliations:** a The Eighth Clinical Medical College, Guangzhou University of Chinese Medicine, Foshan, Guangdong, China; b Foshan Hospital of Traditional Chinese Medicine, Guangzhou University of Chinese Medicine, Foshan, Guangdong, China.

**Keywords:** autoimmune liver disease, chronic hepatitis C, GWAS, Mendelian randomization, NHANES, thyroid disease

## Abstract

Autoimmune liver diseases (AILDs), including autoimmune hepatitis (AIH), primary biliary cholangitis (PBC), and primary sclerosing cholangitis (PSC), often have complex interactions with thyroid diseases (TDs), such as hypothyroidism, hyperthyroidism, and Hashimoto thyroiditis (HT). These conditions frequently coexist and may share common autoimmune mechanisms, but their exact relationship remains poorly understood. Chronic hepatitis C (CHC), a viral liver disease, also affects thyroid function, but its interaction with TD is still under investigation. This study explores the causal links between AILD, CHC, and TD using data from the National Health and Nutrition Examination Survey (NHANES) and Mendelian randomization (MR) analysis. Data were sourced from NHANES (2013–2018) and various genome-wide association studies. The NHANES analysis included 8978 participants after applying inclusion and exclusion criteria. Logistic regression models were used to assess associations between CHC and TD. MR analysis employed single-nucleotide polymorphisms as instrumental variables to investigate causal relationships between AILD, CHC, and TD. NHANES analysis revealed no significant association between CHC and TD. Forward MR analysis indicated significant causal relationships between PBC and hypothyroidism (inverse variance weighting [IVW] odds ratio [OR] = 1.004, 95% confidence intervals [CI] 1.002–1.006, *P* < .001), PSC and hyperthyroidism (IVW OR = 1.002, 95% CI 1.002–1.003, *P* < .001), and PBC and HT (IVW OR = 1.05, 95% CI 1.012–1.089, *P* = .010). Reverse MR analysis suggested causal links between hypothyroidism, hyperthyroidism, HT, thyroid cancer, and AIH, as well as hypothyroidism with PBC and PSC. Multivariable MR confirmed significant associations between AIH and hypothyroidism (*P* < .001), hyperthyroidism (*P* = .008) across IVW method. The IVW method also revealed another significant causal relationship between PSC and hyperthyroidism (*P* < .001), HT (*P* = .013). Multivariable MR also analysis to investigate the association between TD as exposures and AILD as outcomes; the IVW method revealed a noteworthy causal association solely between HT and AIH (*P* = .035). The study identified significant causal associations between AILD (particularly PBC and PSC) and specific TD, emphasizing the need for regular thyroid monitoring in AILD patients. However, no significant causal link was found between CHC and TD.

## 1. Introduction

Autoimmune liver disease (AILD) encompasses a spectrum of disorders characterized by the immune system mistakenly attacking the liver, leading to inflammation and potential liver damage. This category includes autoimmune hepatitis (AIH), primary biliary cholangitis (PBC), and primary sclerosing cholangitis (PSC).^[1]^

AIH often presents with symptoms ranging from acute hepatitis to asymptomatic cases. Diagnosis involves detecting specific autoantibodies, elevated IgG levels, and histological features like interface hepatitis and periportal necrosis.^[2]^ The global prevalence of AIH stands at 17.44 per 100,000 individuals.^[3]^ PBC is a chronic autoimmune disease that leads to the progressive destruction of bile ducts in the liver. This results in impaired bile flow, causing cholestasis, cirrhosis, and potentially liver failure. PBC is often diagnosed through the presence of anti-mitochondrial antibodies, elevated alkaline phosphatase, and elevated immunoglobulin M levels. Other diagnostic features include abnormal liver function tests and histological evidence of bile duct damage.^[4]^ PBC exhibits regional disparities in both incidence and prevalence, with an annual incidence rate of 4.3 per 100,000 individuals in the United States and 0.86 per 100,000 individuals in Korea. The corresponding prevalence rates are 39.2 and 4.75 per 100,000 individuals respectively.^[5,6]^ PSC is a chronic cholestatic liver disease characterized by progressive inflammation and fibrosis of bile ducts, leading to bile duct strictures. The disease typically presents with symptoms of cholestasis such as fatigue, pruritus, and right upper quadrant pain, although some patients may be asymptomatic. Diagnostic confirmation relies on elevated cholestatic liver enzymes like alkaline phosphatase and γ-glutamyl transferase (GGT), alongside imaging findings from cholangiography (typically magnetic resonance cholangiopancreatography) that show bile duct stricturing and dilation.^[7]^ The prevalence of PSC varies across different regions, with rates ranging from 4.15 to 16.2 per 100,000 in Northern Europe and North America, while as low as 0.95 per 100,000 in Japan. This disease exhibits a higher incidence among males and frequently co-occurs with ulcerative colitis, affecting approximately 50% to 80% of patients in Western countries.^[8]^ The presence of TD is intricately associated with AILD.^[9]^ The study conducted by Floreani et al reveals a frequent association between TD, particularly autoimmune thyroid diseases (AITD) like Hashimoto thyroiditis (HT), and PBC.^[10]^ The prevalence of TD in PBC was found to be 13% in Silveira et al’s study, compared to 11% in PSC and 25% in nonalcoholic fatty liver disease.^[11]^

Hepatitis C virus (HCV) is a hepatotropic RNA virus causing acute and chronic hepatitis. Transmission occurs mainly through unsafe medical injections and injecting drug use. Many infected individuals are asymptomatic, complicating early diagnosis. Chronic HCV can lead to severe liver damage, including cirrhosis and cancer. With the introduction of new direct-acting antivirals (DAAs), treatment outcomes have dramatically improved, offering cure rates over 95%.^[12]^ Efforts are ongoing to meet the WHO’s goal to eliminate HCV by 2030 through enhanced screening and access to care.^[13]^ As of 2020, around 57 million people globally have chronic HCV infection, most infections are in low-income and middle-income countries, with China, India, Pakistan, Russia, and the United States bearing the highest burdens.^[14]^ Annually, there are approximately 1.5 million new HCV infections,^[15]^ though rates have declined among injectors in high-income countries but remain high or increasing in some other regions.^[16]^

Hypothyroidism is the most prevalent thyroid disorder observed in patients with chronic hepatitis C (CHC) virus infection. Within this population, clinical hypothyroidism affects up to 13% of individuals, while circulating thyroid antibodies are present in up to 25%.^[17–19]^ The presence of a genetic predisposition seems to contribute to the development of TD in individuals with hepatitis C infection undergoing interferon therapy.^[20]^ In Vezali et al’s study, the administration of antiviral therapy for CHC may potentially reintroduce or exacerbate preexisting asymptomatic thyroid disease (TD). The presence of TD does not seem to be correlated with any virological parameters prior to treatment, including interferon dosage, treatment duration, viral dynamics, or virology results.^[21]^ Therefore, further investigation is required to determine the role of HCV in the development of TD.

The National Health and Nutrition Examination Survey (NHANES), managed by the National Center for Health Statistics under the Centers for Disease Control and Prevention, is a vital source of health and nutritional data in the United States. Its sophisticated multistage probability sampling approach is key to ensuring a representative cross-section of the US population, capturing diverse health and demographic characteristics. Through its rigorous combination of interviews, physical exams, and laboratory tests performed by experienced medical professionals, NHANES provides invaluable insights for public health research and policy-making.^[22–25]^ This rich data collection facilitates a nuanced understanding of national health patterns and influences, proving essential for studies on the relationship between CHC and TD.

Mendelian randomization (MR) enables the establishment of a causal relationship between exposure and outcome. Utilizing single-nucleotide polymorphisms (SNPs) as instrumental variables (IVs),^[26,27]^ the Mendelian laws of inheritance dictate that during meiosis, alleles are transmitted from parents to offspring in a random manner, unaffected by external factors.^[28]^ The use of MR confers a natural advantage in establishing causality by effectively addressing unobserved confounding variables. Moreover, the application of 2-sample MR, which necessitates independent and homogeneous samples for exposure and outcome measurements, provides access to an extensive array of resources and aggregated statistics derived from genome-wide association studies (GWASs).^[29]^ The utilization of MR can effectively mitigate confounding factors and attenuate the inherent bias commonly observed in observational studies. By leveraging genetic tools that are closely associated with specific risk factors, MR empowers researchers to reliably ascertain the causal effects of these factors on a diverse range of diseases and conditions. This pioneering technology is increasingly pivotal in epidemiology and public health as an indispensable instrument for elucidating the underlying biological mechanisms behind diseases and formulating targeted interventions.^[30]^

## 2. Methods

### 2.1. Research on CHC and TD in NHANES

**Figure 1.**
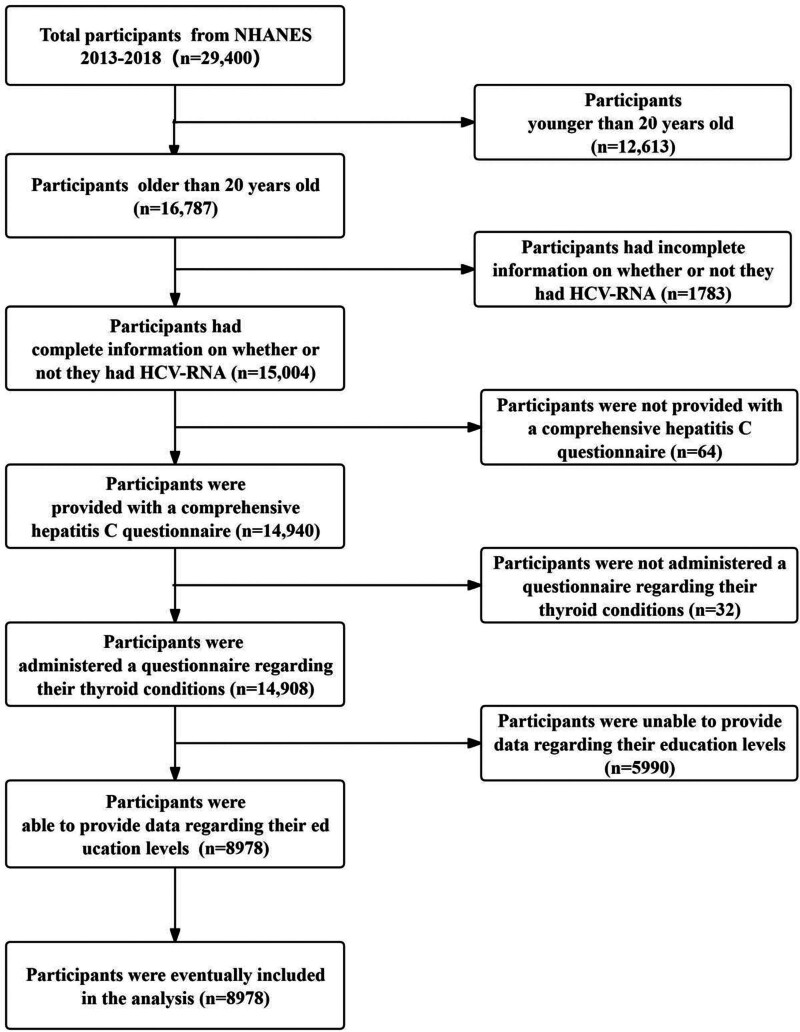
Flowchart of participants’ inclusion. HCV = hepatitis C virus, NHANES = National Health and Nutrition Examination Survey.

The data for this study were obtained from the NHANES database, available at https://www.cdc.gov/nchs/nhanes/?CDC_AAref_Val=https://www.cdc.gov/nchs/nhanes/index.htm. The protocols of the NHANES study have been approved by the National Center for Health Statistics Research Ethics Review Board, and all participants provided informed consent. Since this research utilized anonymized, publicly available data, it was exempt from obtaining institutional review board approval. The inclusion criteria for the study targeted participants who attended the NHANES Mobile Examination Center from 2013 to 2018 and met specific eligibility requirements. The participants included in this group meet the specified requirement: the minimum age requirement is 20 years or older. The study sample comprised 16,787 individuals who met the specified eligibility criteria. The exclusion criteria were as follows: participants had incomplete information on whether or not they had HCV-RNA (n = 1783); participants were not provided with a comprehensive hepatitis C questionnaire (n = 64); participants were not administered a questionnaire regarding their thyroid conditions (n = 32); participants were unable to provide data regarding their education levels (n = 5990). The study ultimately encompassed a total of 8978 participants. The specifics can be found in Figure 1.

The yes or no questionnaires were utilized for the purpose of diagnosing participants’ hepatitis C and thyroid problem. To mitigate potential confounding effects, adjustments were made for the following demographic characteristics: age, gender, education level, ratio of family income to poverty, and laboratory features: HCV-RNA, aspartate transaminase, alanine aminotransferase, and GGT.

This study investigated the association between CHC and TD utilizing data from NHANES, employing a weighted logistic regression framework across 3 distinct models. Model 1 evaluated the unadjusted relationship, while Model 2 accounted for demographic and socioeconomic factors (age, gender, education level, and family income-to-poverty ratio). Model 3 further adjusted for HCV-RNA, aspartate transaminase, alanine aminotransferase, and GGT. Associations were determined through the Wald test (*P* < .05 indicating significance), and model adequacy was assessed using the Akaike Information Criterion. Findings were presented as odds ratios (ORs) or beta coefficients with corresponding 95% confidence intervals (CI). Considering the complex probability clustering design of NHANES, the study accounted for weights in the statistical analysis.

### 2.2. Research on AILD, CHC, and TD in GWAS sources

#### 2.2.1. Source of data and selection and validation of IVs

The genetic variants with a strong association to AIH were sourced from Sakaue et al study,^[31]^ which encompasses data from 485,234 individuals of European descent, including 821 cases and 484,413 controls. CHC infection dataset was also sourced from Sakaue et al study,^[31]^ which encompasses data from 352,013 individuals of European descent, including 273 cases and 351,740 controls. We sourced genetic data pertaining to PBC from Cordell et al study, comprising entirely of European individuals, with a sample that included 8021 cases and 16,489 controls.^[32]^ International PSC Study Group provided the PSC with data, including a sample size of 14,890, consisting of 2871 cases and 12,019 controls.^[33]^ TD include hypothyroidism, hyperthyroidism, HT, and thyroid cancer. Hypothyroidism and hyperthyroidism dataset were sourced from the MRC Integrated Epidemiology Unit consortium, and HT and thyroid cancer dataset were also sourced from Sakaue et al study.^[31]^ We accessed the GWAS data through the Integrative Epidemiology Unit’s OpenGWAS database at https://gwas.mrcieu.ac.uk/. Each study had received the requisite ethical approvals from the respective institutional review boards, and informed consent was obtained from all contributors.

**Figure 2.**
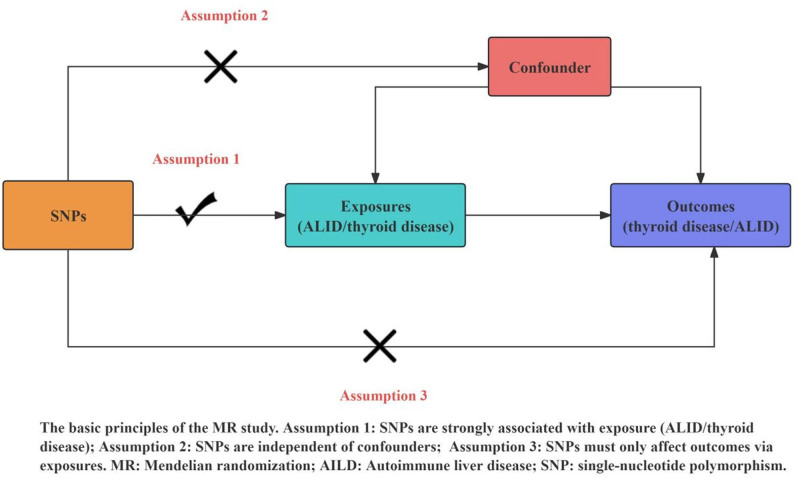
Foundations of Mendelian genetics: the 3 assumptions of randomization.

To investigate the causal relationships between TD and specific exposures, including AIH, PBC, PSC, and CHC infection, SNPs were utilized as IVs for the analysis. This study adhered to the 3 essential assumptions of 2-sample MR: 1. *SNP selection and instrument strength*: the selected IVs demonstrated robust associations with the exposure, confirmed by a significance level (*P* < 5 × 10⁻^6^) and an *F*-statistic >10. The *F*-statistic quantifies the proportion of variance in the exposure explained by the SNPs, providing an assessment of the instrument strength. A higher *F*-statistic indicates a stronger instrument and reduces the risk of weak instrument bias, which can lead to unreliable causal estimates. The *F*-statistic was calculated using the following formula: $F=\frac{R^{2}\times \left(N-2\right)}{1-R^{2}}$, where *R*^2^ represents the proportion of variance explained by the SNPs, *N* is the sample size, and *K* is the number of SNPs used in the analysis. SNPs with *F*-statistics >10 were considered sufficiently strong to ensure the validity of the MR analysis, minimizing potential bias. 2. *Independence from confounding*: the IVs were required to be independent of confounding factors between the exposure and outcome. To ensure this, we excluded SNPs exhibiting linkage disequilibrium with an *R*^2^ < 0.001 across a 10,000 kb range. This ensures that the selected SNPs were not correlated with other genetic variants that might confound the causal relationship between the exposure and the outcome. Furthermore, we employed the PhenoScanner database and LDlink (https://ldlink.nci.nih.gov/?tab=ldtrait) to systematically verify that each SNP was not associated with any confounders or traits linked to TD. This rigorous approach helped to mitigate the potential for confounding and ensure the reliability of our instruments. 3. *Exclusion of pleiotropy*: The IVs were assumed to affect the outcome solely through the exposure, without any other intervening pathways. To ensure this assumption was met, we conducted MR-Egger regression and other sensitivity analyses to assess for horizontal pleiotropy. If pleiotropy was detected, we adjusted for it using random-effects models or by excluding problematic SNPs from the analysis. This step is crucial to ensure that the SNPs influence the outcome only via the exposure of interest, maintaining the integrity of the causal inference. Figure 2 provides a visual representation of the SNP harmonization process and how the IVs were selected and validated.

#### 2.2.2. MR design and statistical analyses

In our 2-sample MR analysis, the exposure and outcome datasets were coordinated to ensure consistent referencing of the same allele. In order for SNPs to be selected as IVs, they must demonstrate effectiveness. Various methods, including inverse variance weighting (IVW), MR-Egger method, weighted median method, weighted model method, and simple model method, were employed to investigate the potential causal relationship between AILD, CHC and TD. The detailed overview of the data downloading and filtering process is presented in Table 1. The reverse MR analysis was conducted to assess the potential reverse causality between AILD and TD.

**Table 1:** Characteristics of GWAS participants included in the MR study.

Items	GWAS ID	Consortium	Sample size	PMID	Population
AIH	ebi-a-GCST90018785	NA	485,234	34594039	Europeans
PBC	ebi-a-GCST90061440	NA	24,510	34033851	Europeans
PSC	ieu-a-1112	NA	24,510	34033851	Europeans
CHC infection	ebi-a-GCST90018805	NA	352,013	34594039	Europeans
Hypothyroidism	ukb-b-19732	MRC-IEU	462,933	NA	Europeans
Hyperthyroidism	ukb-b-20289	MRC-IEU	462,933	NA	Europeans
HT	ebi-a-GCST90018855	NA	395,640	34594039	Europeans
Thyroid cancer	ebi-a-GCST90018929	NA	491,974	34594039	Europeans

AIH = autoimmune hepatitis, CHC = chronic hepatitis C, GWAS = genome-wide association studies, HT = Hashimoto thyroiditis, MR =Mendelian randomization, PBC = primary biliary cholangitis, PSC = primary sclerosing cholangitis.

In this study, we performed a multivariable Mendelian randomization (MVMR) analysis to explore the causal relationships between AILDs (AIH, PSC, and PBC) and TDs (hypothyroidism, hyperthyroidism, and HT). The analysis was conducted in 2 separate steps to examine the bidirectional causal relationships between these diseases. In the first part of the MVMR analysis, we examined the causal relationships between the 3 AILDs (AIH, PSC, and PBC) and each of the 3 TDs (hypothyroidism, hyperthyroidism, and HT) individually. For each liver disease, we tested its causal effect on each thyroid disorder using SNPs as IVs, accounting for potential confounding factors and shared genetic pathways. This step helped us identify whether specific AILDs were causally associated with thyroid dysfunction. The second part of the analysis focused on the reverse causal relationships, where we investigated whether TDs (hypothyroidism, hyperthyroidism, and HT) could have causal effects on AILDs (AIH, PSC, and PBC). For each TD, we tested its potential causal impact on each liver disease, using the same method of SNPs as IVs to control for confounding factors and shared genetic influences.

#### 2.2.3. Sensitivity analysis

A sensitivity analysis was conducted to assess possible departures from the model assumptions in the MR analysis. This evaluation included 3 specific tests: the heterogeneity test, the pleiotropic test, and the leave-one-out test.^[34,35]^ The heterogeneity of IVs was evaluated using Cochrane *Q* test.^[36,37]^ The results of the heterogeneity test indicate the presence of heterogeneity when the *P*-value is <.05. To account for potential heterogeneity’s impact on the findings, a random effects model was employed. Alternatively, a fixed effects model was utilized to assess causal relationship.

In addition to these tests, we employed MR Pleiotropy RESidual Sum and Outlier to assess and address potential pleiotropy. Specifically, MR Pleiotropy RESidual Sum and Outlier was used to detect SNP outliers that might contribute to horizontal pleiotropy. SNPs identified as outliers were excluded from the analysis to ensure that the IVs met the assumptions of the MR model. This process helped to further ensure the robustness of the results and reduce the potential bias due to pleiotropy.The MR-Egger regression test was employed to assess the impact of horizontal pleiotropy and ensure that the IV selected adheres to the fundamental assumptions of the MR analysis. In cases where the *P*-value < .05, it indicates instability in the results.^[29]^ Furthermore, leave-one-out test is employed to evaluate whether individual SNPs exert a disproportionate influence on the overall estimation. This is achieved by sequentially excluding each SNP and applying the IVW method to the remaining set of SNPs.

**Figure 3.**
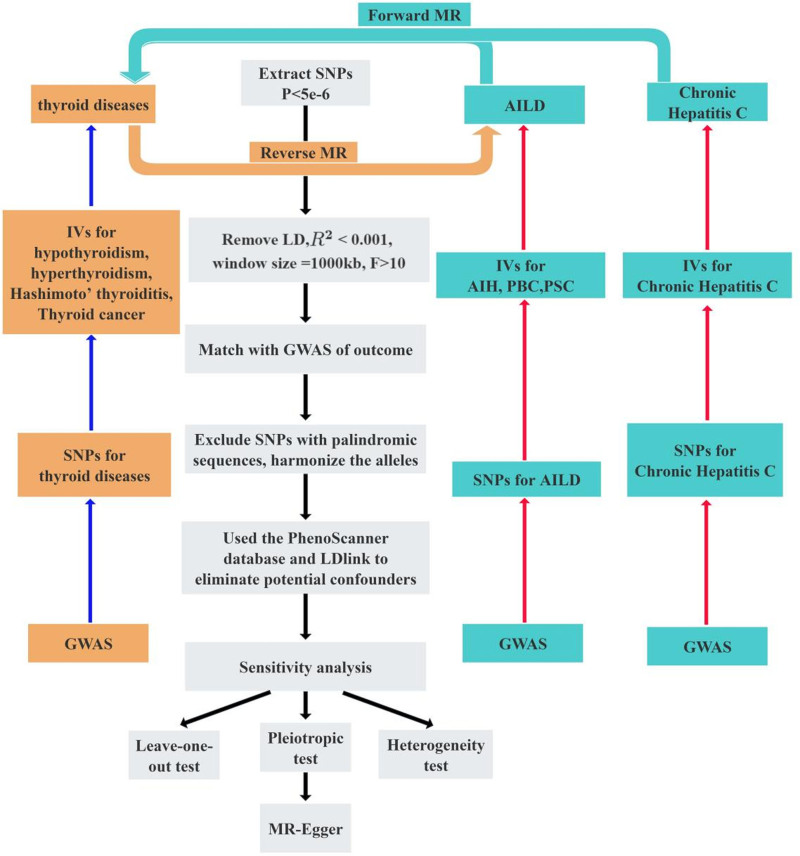
Flowchart of forward and reverse Mendelian randomization methods. AIH = autoimmune hepatitis, AILD = autoimmune liver disease, GWAS = genome-wide association studies, IV = instrumental variable, LD = linkage disequilibrium, MR =Mendelian randomization, PBC = primary biliary cholangitis, PSC = primary sclerosing cholangitis, SNPs = single-nucleotide polymorphisms.

The statistical analyses were conducted using R software, version 4.3.1 (R Foundation, Vienna, Austria), and EmpowerStats software (X&Y Solutions Inc., Boston). In this study, several R packages were utilized to conduct the MR and MVMR analyses. TwoSampleMR was used to extract exposure and outcome data, harmonize datasets, and calculate causal estimates using methods such as IVW and MR-Egger. The MendelianRandomization package enabled the application of various MR methods, including IVW, Egger regression, and weighted median methods, along with robust heterogeneity tests to assess the validity of IVs. The MVMR package was employed to perform MVMR analysis, utilizing functions such as strength_mvmr for *F*-statistics, pleiotropy_mvmr for testing pleiotropy, and ivw_mvmr for causal effect estimation while considering multiple exposures. robustbase was used for robust regression analysis to account for potential outliers and heteroscedasticity, ensuring more reliable causal estimates. Additionally, the tidyverse, RColorBrewer, cowplot, and gridExtra packages were employed for data manipulation, visualization, and presentation, enhancing the clarity and quality of the results. These R packages allowed for comprehensive and robust MR and MVMR analyses, ensuring accurate causal inference while controlling for pleiotropy and heterogeneity. The criterion for statistical significance was defined as a *P*-value below .05. The flow chart for IVs screening and the steps of MR analysis are illustrated in Figure 3.

## 3. Results

### 3.1. Demographic and clinical characteristics in the NHANES study were statistically based on hepatitis C

According to demographic and clinical characteristics from the NHANES data in Table 2, participants were categorized into 2 groups: 138 individuals with hepatitis C and 8780 without. The analysis revealed that patients with hepatitis C are generally older than those without. Furthermore, a higher prevalence of hepatitis C was observed among males, and the prevalence of hepatitis C does not appear to be associated with the level of education. Interestingly, the incidence of thyroid problems among individuals with hepatitis C is significantly lower compared to those without thyroid problems.

**Table 2:** Demographic and clinical characteristics of the participants with and without hepatitis C.

Items	Hepatitis C (n = 138)	No hepatitis C (n = 8780)	*P*-value
Age (yr)	55.1 (52.4–57.8)	48.8 (48.1–49.6)	.0001*
Gender			<.001†
Male	92 (66.7%)	4218 (48.0%)	
Female	46 (33.3%)	4562 (52.0%)	
Education level			.02†
Less than high school degree	24 (17.4%)	1760 (20.0%)	
High school grad/GED or some college/AA degree	75 (54.3%)	3745 (42.7%)	
College graduate or above	39 (28.3%)	3275 (37.3%)	
Ratio of family income to poverty	2.1 (1.6–2.6)	2.7 (2.6–2.8)	.0286*
Hepatitis C RNA			<.001†
Positive	65 (47.1%)	51 (0.6%)	
Negative	52 (37.7%)	87 (1.0%)	
Negative screening HCV antibody	21 (15.2%)	8642 (98.4%)	
Thyroid problem			.121†
Yes	10 (7.2%)	1008 (11.5%)	
No	128 (92.8%)	7772 (88.5%)	
Laboratory features			
AST (U/L)	37.1 (30.3–44.0)	24.0 (23.5–24.5)	.0005*
GGT (IU/L)	50.4 (40.4–60.4)	29.5 (28.6–30.5)	<.0001*
ALT (U/L)	39.5 (30.6–48.3)	23.3 (23.0–23.7)	.0008*

Data were presented as median (interquartile range) or n (%).

AA = Associate of arts, ALT = alanine aminotransferase, AST = aspartate transaminase, GED = General Educational Development, GGT = gamma-glutamyl transferase, HCV = hepatitis C virus.

* Mann–Whitney *U* test for continuous variables.

† Pearson chi-squared test for categorical variables.

### 3.2. Observational associations between hepatitis C and thyroid problem in NHANES

Our weighted logistic regression analysis indicates that there is no strong and significant association between hepatitis C and the risk of thyroid issues, consistent across models adjusting for various covariates. In Model 1, the results indicate no significant association between hepatitis C and thyroid problems (OR = 0.602 [95% CI 0.316–1.149], *P* = .124). Similarly, Models 2 (OR = 0.634 [95% CI 0.315–1.274], *P* = .201) and 3 (OR = 0.580 [95% CI 0.258–1.302], *P* = .186) also demonstrate no correlation with thyroid problems. The results of the analysis are shown in Table 3.

**Table 3:** Odds ratios (ORs) for the association between hepatitis C and thyroid problem.

Thyroid problem	Hepatitis C	No Hepatitis C	*P*-value
Model 1 OR (95% CI)	0.602 (0.316–1.149)	0	.124
Model 2 OR (95% CI)	0.634 (0.315–1.274)	0	.201
Model 3 OR (95% CI)	0.580 (0.258–1.302)	0	.186

Model 1: non-adjusted; Model 2: adjusted for age, gender, education level, and family income-to-poverty ratio; Model 3: adjusted for HCV-RNA, AST, GGT, and ALT.

ALT = alanine aminotransferase, AST = aspartate transaminase, CI = confidence interval, GGT = gamma-glutamyl transferase, HCV = hepatitis C virus

### 3.3. The causal effect of AILD and CHC infection on TD via forward MR

**Figure 4.**
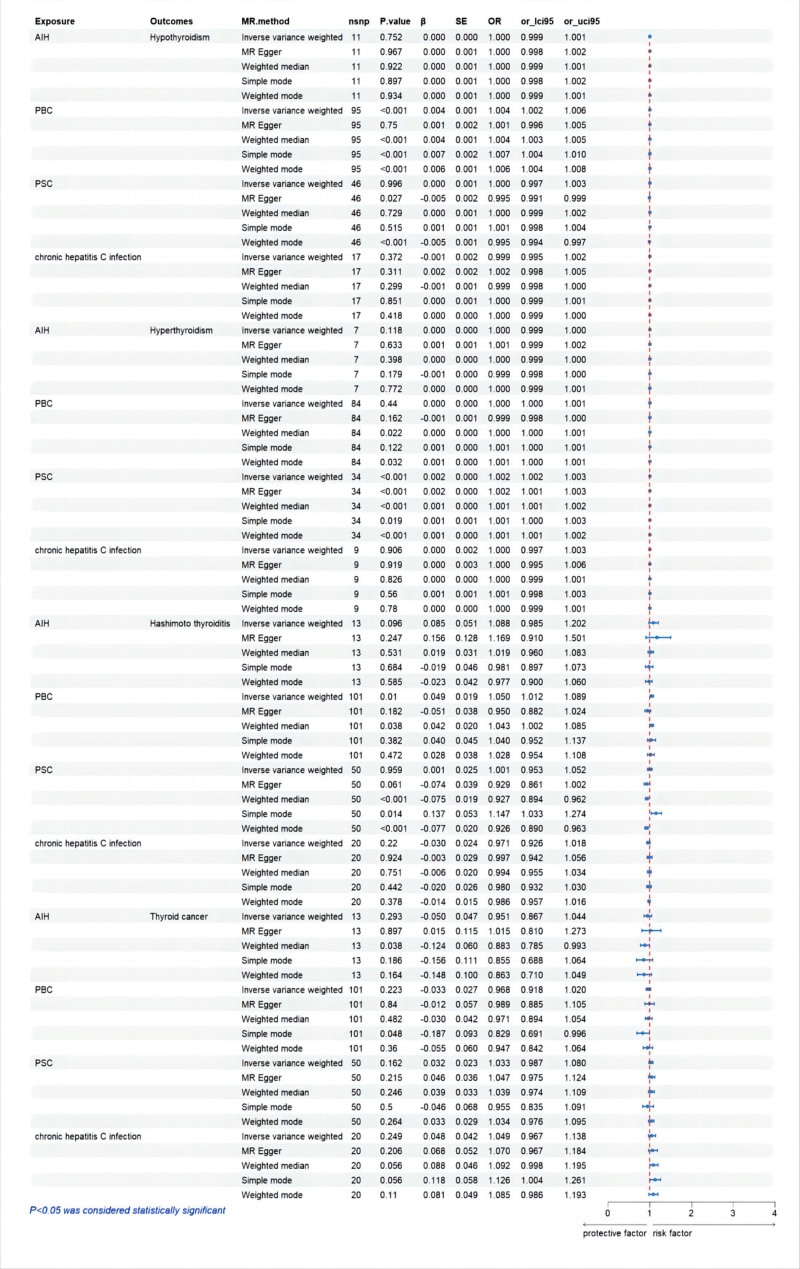
Forest plot of forward two-sample Mendelian randomization analysis for exposure effects. AIH = autoimmune hepatitis, OR = odds ratio, PBC = primary biliary cholangitis, PSC = primary sclerosing cholangitis.

To evaluate the relationship between AILD and CHC, and the likelihood of developing TD, MR analysis was conducted. The analysis confirmed a statistically significant correlation, with a *P*-value <.05. Table S1, Supplemental Digital Content, https://links.lww.com/MD/P760 describes the selected SNPs used as IVs to test for causal relationships. All results are presented in the form of a forest plot in Figure 4. We observed a significant causal association using the IVW method between AILD and TD (PBC and hypothyroidism, IVW OR = 1.004, 95% CI 1.002–1.006, *P* < .001; PBC and HT, IVW OR = 1.05, 95% CI 1.012–1.089, *P* = .010; PSC and hyperthyroidism, IVW OR = 1.002, 95% CI 1.002–1.003, *P* < .001) (Figures S1A and C and S2B, Supplemental Digital Content, https://links.lww.com/MD/P759). The causal relationships among these 3 are also significant and consistent in the weighted median results (PBC and hypothyroidism, *P* < .001; PBC and HT, *P* = .038; PSC and hyperthyroidism, *P* < .001). By employing leave-one-out sensitivity analysis, individual genetic variations’ influence on the overall results can be determined, further confirming the robustness of the MR analysis results (Figures S3A and C and S4B, Supplemental Digital Content, https://links.lww.com/MD/P759). The MR-Egger regression test indicates that horizontal pleiotropy is not significant (PBC and hypothyroidism, *P* = .122; PSC and hyperthyroidism, *P* = .807) (Figures S5A and C and S6B, Supplemental Digital Content, https://links.lww.com/MD/P759; Table S2, Supplemental Digital Content, https://links.lww.com/MD/P760). These findings may suggest that the causal relationships between PBC and hypothyroidism, as well as PSC and hyperthyroidism, are stable and unbiased. Contrarily, MR-Egger regression analysis revealed significant horizontal pleiotropy between PBC and HT (*P* = .004). This could suggest that the causal relationship between PSC and HT is unstable and biased. Statistical heterogeneity was detected using Cochran *Q* statistics test in the IVW analysis (PBC and hypothyroidism, Cochran *Q* = 1387.100, *P* < .001; PBC and HT, Cochran *Q* = 307.253, *P* < .001; PSC and hyperthyroidism, Cochran *Q* = 305.700, *P* < .001) (Figures S7A and C and S8B, Supplemental Digital Content, https://links.lww.com/MD/P759; Table S2, Supplemental Digital Content, https://links.lww.com/MD/P760). Therefore, the IVW method employs a multiplicative random-effects model to assess the causal relationship between them. There is no causal relationship between PBC and hyperthyroidism or thyroid cancer, nor is there a causal relationship between PSC and hypothyroidism, HT, or thyroid cancer (Figures S1, S3, S5, S7B and D and Figures S2, S4, S6, S8A, C and D, Supplemental Digital Content, https://links.lww.com/MD/P759; Table S2, Supplemental Digital Content, https://links.lww.com/MD/P760).

Summarizing the results from MR, there are causal relationships between PBC and hypothyroidism, PSC and hyperthyroidism, and PBC and HT. The inclusion of all sensitivity analyses as qualitative control measures collectively indicates a weak bias in the causal associations. MR analysis also indicates no causal relationship between AIH, CHC, and the 4 types of TD (Figures S9 and S10, Figures S11 and S12, Figures S13 and S14, Figures S15 and S16, Supplemental Digital Content, https://links.lww.com/MD/P759; Table S2, Supplemental Digital Content, https://links.lww.com/MD/P760).

### 3.4. The causal effect of TD on AILD via reverse MR

In the reverse MR analysis, we further investigate by considering TDs as the exposure and AILD as the outcomes to assess the causal relationships between them. Considering the selection criteria, we retrieved specific SNPs associated with AILD, aiming to study the impact of TD on AILD (Table S3, Supplemental Digital Content, https://links.lww.com/MD/P760). All results are presented in the form of a forest plot in Table 4. The IVW analysis method has demonstrated a significant association between hypothyroidism (*P* < .001), hyperthyroidism (*P* = .001), HT (*P* < .001), thyroid cancer (*P* = .012), and AIH (Figure S17, Supplemental Digital Content, https://links.lww.com/MD/P759); the IVW method also showed a significant causal relationship between hypothyroidism (*P* = .001) and PBC (Figure S18A, Supplemental Digital Content, https://links.lww.com/MD/P759); this method also provided compelling evidence of a significant causal relationship between hypothyroidism (*P* < .001), hyperthyroidism (*P* < .001), HT (*P* = .012) and PSC (Figure S19A–C, Supplemental Digital Content, https://links.lww.com/MD/P759). Leave-one-out analysis was employed to evaluate the robustness of the obtained results (Figures S20A–C, S21A, and S22A–C, Supplemental Digital Content, https://links.lww.com/MD/P759). The MR-Egger regression analysis was conducted to evaluate the presence of horizontal pleiotropy. The outcomes indicated that horizontal pleiotropy was improbable to influence the causal relationships involving hypothyroidism (intercept = 0.004, *P* = .613) (Figure S23A, Supplemental Digital Content, https://links.lww.com/MD/P759; Table S2, Supplemental Digital Content, https://links.lww.com/MD/P760), thyroid cancer (intercept = −0.005, *P* = .518) (Figure S23D, Supplemental Digital Content, https://links.lww.com/MD/P759; Table S2, Supplemental Digital Content, https://links.lww.com/MD/P760), and AIH; hypothyroidism (intercept = 0.026, *P* = .185) (Figure S24A, Supplemental Digital Content, https://links.lww.com/MD/P759; Table S2, Supplemental Digital Content, https://links.lww.com/MD/P760) and PBC; hypothyroidism (intercept = −0.006, *P* = .622) (Figure S25A, Supplemental Digital Content, https://links.lww.com/MD/P759; Table S2, Supplemental Digital Content, https://links.lww.com/MD/P760), hyperthyroidism (intercept = −0.164, *P* = .090) (Figure S25B, Supplemental Digital Content, https://links.lww.com/MD/P759; Table S2, Supplemental Digital Content, https://links.lww.com/MD/P760), HT (intercept = −0.015, *P* = .679) (Figure S25C, Supplemental Digital Content, https://links.lww.com/MD/P759; Table S2, Supplemental Digital Content, https://links.lww.com/MD/P760), and PSC. It is noteworthy that horizontal pleiotropy exists between hyperthyroidism (intercept = −0.067, *P* = .024) (Figure S23B, Supplemental Digital Content, https://links.lww.com/MD/P759; Table S2, Supplemental Digital Content, https://links.lww.com/MD/P760), HT (intercept = −0.044, *P* = .029) (Figure S23C, Supplemental Digital Content, https://links.lww.com/MD/P759; Table S2, Supplemental Digital Content, https://links.lww.com/MD/P760), and AIH. We conducted heterogeneity testing using IVW, revealing heterogeneity in the estimated values of the included SNPs. Therefore, we employed a multiplicative random-effects model to examine the causal relationship (hypothyroidism on AIH, Cochran *Q* = 394.065, *P* = .001; hyperthyroidism on AIH, Cochran *Q* = 91.331, *P* < .001; HT on AIH, Cochran *Q* = 87.316, *P* = .012; hypothyroidism on PBC, Cochran *Q* = 854.062, *P* < .001; hypothyroidism on PSC, Cochran *Q* = 892.241, *P* < .001; hyperthyroidism on PSC, Cochran *Q* = 269.278, *P* < .001; HT on PSC, Cochran *Q* = 251.173, *P* < .001) (Figure S26A–C, S27A, and S28A–C, Supplemental Digital Content, https://links.lww.com/MD/P759; Table S2, Supplemental Digital Content, https://links.lww.com/MD/P760). Only in the case of thyroid cancer and AIH was heterogeneity absent (Cochran *Q* = 48.068, *P* = .511) (Figure S26D, Supplemental Digital Content, https://links.lww.com/MD/P759; Table S2, Supplemental Digital Content, https://links.lww.com/MD/P760). Therefore, we utilized a fixed effects model to evaluate the causal relationship between the 2.

**Table 4:** The results of thyroid disease on AILD via reverse MR.

Exposure	Outcomes	MR method	nsnp	β	SE	OR	or_lci95	or_uci95	*P*-value
Hypothyroidism	AIH	Inverse variance weighted	312	6.048	1.008	423.211	58.634	3054.649	<.001
		MR Egger	312	5.056	2.207	156.926	2.077	11858.951	.023
		Weighted median	312	4.593	1.729	98.768	3.332	2927.860	.008
		Simple mode	312	4.856	4.506	128.564	0.019	880067.005	.282
		Weighted mode	312	3.957	2.357	52.280	0.515	5305.712	.094
Hyperthyroidism		Inverse variance weighted	45	29.874	9.196	9.42285E+12	140057.398	6.34E+20	.001
		MR Egger	45	73.157	20.430	5.91E+31	2.40727E+14	1.45E+49	.001
		Weighted median	45	32.024	10.279	8.09081E+13	143923.876	4.55E+22	.002
		Simple mode	45	41.517	19.672	1.07265E+18	19.273	5.97E+34	.041
		Weighted mode	45	35.982	11.696	4.23573E+15	468537.161	3.83E+25	.004
Hashimoto thyroiditis		Inverse variance weighted	61	0.347	0.073	1.414	1.225	1.632	<.001
		MR Egger	61	0.617	0.140	1.854	1.410	2.437	<.001
		Weighted median	61	0.306	0.101	1.358	1.114	1.655	.002
		Simple mode	61	0.112	0.220	1.119	0.727	1.722	.612
		Weighted mode	61	0.276	0.188	1.318	0.913	1.904	.146
Thyroid cancer		Inverse variance weighted	50	−0.029	0.012	0.971	0.949	0.994	.012
		MR Egger	50	−0.021	0.017	0.979	0.947	1.012	.217
		Weighted median	50	−0.027	0.019	0.974	0.939	1.010	.148
		Simple mode	50	−0.120	0.039	0.887	0.822	0.958	.004
		Weighted mode	50	−0.027	0.017	0.973	0.941	1.006	.116
Hypothyroidism	PBC	Inverse variance weighted	83	6.709	2.000	820.071	16.268	41339.880	.001
		MR Egger	83	1.579	4.327	4.851	0.001	23385.961	.716
		Weighted median	83	5.016	1.129	150.873	16.490	1380.391	<.001
		Simple mode	83	3.925	2.130	50.667	0.780	3293.131	.069
		Weighted mode	83	5.054	1.134	156.686	16.981	1445.739	.000
Hyperthyroidism		Inverse variance weighted	26	−4.524	9.379	0.011	1.13E−10	1043690.350	.630
		MR Egger	26	3.221	18.159	25.052	8.74E−15	7.18371E+16	.861
		Weighted median	26	−7.388	4.615	0.001	7.30E−08	5.246	.109
		Simple mode	26	−7.736	8.175	0.000	4.80E−11	3970.572	.353
		Weighted mode	26	−5.271	4.234	0.005	1.28E−06	20.644	.225
Hashimoto thyroiditis		Inverse variance weighted	34	0.173	0.090	1.189	0.997	1.419	.054
		MR Egger	34	0.047	0.176	1.048	0.742	1.481	.791
		Weighted median	34	0.021	0.063	1.022	0.903	1.156	.732
		Simple mode	34	−0.041	0.115	0.959	0.765	1.203	.721
		Weighted mode	34	−0.049	0.091	0.952	0.796	1.138	.592
Thyroid cancer		Inverse variance weighted	7	0.018	0.026	1.018	0.967	1.071	.498
		MR Egger	7	−0.048	0.077	0.953	0.820	1.107	.558
		Weighted median	7	0.029	0.035	1.030	0.961	1.104	.406
		Simple mode	7	0.044	0.053	1.045	0.943	1.159	.432
		Weighted mode	7	0.044	0.042	1.045	0.962	1.136	.336
Hypothyroidism	PSC	Inverse variance weighted	225	6.388	1.369	594.833	40.649	8704.498	<.001
		MR Egger	225	7.761	3.101	2347.624	5.380	1024381.378	.013
		Weighted median	225	3.839	1.348	46.489	3.310	652.896	.004
		Simple mode	225	2.646	3.777	14.095	0.009	23139.531	.484
		Weighted mode	225	3.002	3.914	20.133	0.009	43191.588	.444
Hyperthyroidism		Inverse variance weighted	17	105.034	16.653	4.13E+45	2.76E+31	6.18E+59	<.001
		MR Egger	17	167.455	25.399	5.30E+72	1.27E+51	2.21E+94	<.001
		Weighted median	17	79.066	12.578	2.18E+34	4.28E+23	1.11E+45	<.001
		Simple mode	17	19.082	16.142	1.94E+08	3.53E−06	1.07E+22	.254
		Weighted mode	17	157.121	15.869	1.72E+68	5.36E+54	5.55E+81	<.001
Hashimoto thyroiditis		Inverse variance weighted	42	0.303	0.121	1.354	1.068	1.718	.012
		MR Egger	42	0.416	0.296	1.515	0.848	2.709	.169
		Weighted median	42	0.095	0.091	1.099	0.920	1.314	.299
		Simple mode	42	0.008	0.161	1.008	0.735	1.382	.963
		Weighted mode	42	0.034	0.154	1.034	0.764	1.400	.829
Thyroid cancer		Inverse variance weighted	10	0.048	0.040	1.049	0.971	1.134	.225
		MR Egger	10	−0.044	0.095	0.957	0.795	1.153	.658
		Weighted median	10	0.083	0.049	1.086	0.987	1.195	.090
		Simple mode	10	0.049	0.078	1.050	0.900	1.224	.551
		Weighted mode	10	0.074	0.049	1.077	0.979	1.185	.163

AIH = autoimmune hepatitis, AILD = autoimmune liver disease, MR =Mendelian randomization, OR = odds ratio, PBC = primary biliary cholangitis, PSC = primary sclerosing cholangitis, SE = standard error.

Additionally, summary data obtained from the IVW analysis indicates a lack of evidence to assess causality between hyperthyroidism (*P* = .630), HT (*P* = .054), thyroid cancer (*P* = .498), and PBC; thyroid cancer (*P* = .225) and PSC (Figures S21B–D, S22D, S27B–D, S28D, S24B–D, S25D, S18B–D, S19D, and S20D, Supplemental Digital Content, https://links.lww.com/MD/P759; Table S2, Supplemental Digital Content, https://links.lww.com/MD/P760).

### 3.5. The causal effect of AILD on TD via MVMR

Following the completion of our initial 2-sample MR analysis, we extended our inquiry by employing the MVMR framework. This approach enables us to address both the potential confounding effects of correlated exposures and any mediating effects. The MVMR analysis integrates various methods including the IVW method, the median method, and MR-Egger regression to ensure the robustness and comprehensiveness of our findings. All the results are presented in Table 5.

**Table 5:** The results of AILD and thyroid disease via MVMR.

Exposures	Outcomes	MVMR method	β	SE	OR	or_lci95	or_upci95	*P*-value
AIH	Hypothyroidism	Inverse variance weighted	0.019	0.004	1.019	1.010	1.027	**<.001**
		Median	0.010	0.003	1.010	0.004	0.015	.000
		MR-Egger	0.022	0.006	0.018	0.010	0.034	.000
PBC		Inverse variance weighted	0.003	0.002	1.003	0.999	1.006	.120
		Median	0.003	0.001	0.002	0.001	0.005	.001
		MR-Egger	0.003	0.002	NA	−0.004	0.005	.090
PSC		Inverse variance weighted	0.001	0.002	1.001	0.996	1.005	.713
		Median	0.003	0.001	0.000	0.000	0.006	.025
		MR-Egger	0.000	0.001	NA	−0.002	0.001	.482
AIH	Hyperthyroidism	Inverse variance weighted	0.002	0.001	1.002	1.001	1.004	**.008**
		Median	0.000	0.001	NA	−0.001	0.002	.822
		MR-Egger	0.002	0.001	NA	−0.001	0.004	.208
PBC		Inverse variance weighted	0.000	0.000	1.000	0.999	1.000	.584
		Median	0.000	0.000	0.000	0.000	0.001	.210
		MR-Egger	0.000	0.000	NA	−0.001	0.000	.464
PSC		Inverse variance weighted	0.002	0.000	1.002	1.002	1.003	**<.001**
		Median	0.001	0.001	0.000	0.000	0.003	.082
		MR-Egger	0.003	0.001	0.002	0.002	0.004	.000
AIH	HT	Inverse variance weighted	0.127	0.091	1.136	0.950	1.358	.163
		Median	0.181	0.084	0.074	0.016	0.346	.031
		MR-Egger	0.140	0.142	NA	−0.137	0.417	.323
PBC		Inverse variance weighted	0.053	0.031	1.054	0.992	1.121	.089
		Median	0.033	0.084	NA	−0.026	0.092	.270
		MR-Egger	0.054	0.034	NA	−0.012	0.121	.110
PSC		Inverse variance weighted	0.110	0.044	1.116	1.023	1.216	**.013**
		Median	0.083	0.030	NA	−0.005	0.171	.066
		MR-Egger	0.106	0.052	0.032	0.005	0.207	.039
Hypothyroidism	AIH	Inverse variance weighted	0.353	0.280	1.423	0.822	2.464	.208
		Median	0.394	0.290	NA	−0.173	0.962	.173
		MR-Egger	0.313	0.281	NA	−0.238	0.864	.266
Hyperthyroidism		Inverse variance weighted	−2.836	4.876	0.059	4.15E−06	828.940	.561
		Median	−5.921	5.109	NA	−15.935	4.093	.247
		MR-Egger	1.308	5.922	NA	−10.298	12.915	.825
HT		Inverse variance weighted	28.959	13.707	3.77315E+12	8.105	1.76E+24	**.035**
		Median	28.165	15.145	NA	−1.519	57.848	.063
		MR-Egger	28.580	13.678	NA	1.773	55.388	.037
Hypothyroidism	PBC	Inverse variance weighted	0.079	0.347	1.082	0.548	2.136321302	.820
		Median	0.127	0.191	NA	−0.248	0.501	.507
		MR-Egger	0.102	0.346	NA	−0.577	0.781	.768
Hyperthyroidism		Inverse variance weighted	8.636	6.600	5.63E+03	0.014	2.34E+09	.191
		Median	2.750	3.418	NA	−3.950	9.449	.421
		MR-Egger	3.313	8.011	NA	−12.388	19.015	.679
Hashimoto thyroiditis		Inverse variance weighted	−9.666	20.760	6.34E−05	1.35E−22	2.97322E+13	.641
		Median	2.914	9.832	NA	−16.355	22.184	.767
		MR-Egger	−10.256	20.702	NA	−50.830	30.318	.620
Hypothyroidism	PSC	Inverse variance weighted	−0.047	0.216	0.954	0.624	1.458	.829
		Median	0.130	0.146	NA	−0.155	0.416	.371
		MR-Egger	−0.044	0.224	NA	−0.483	0.396	.846
Hyperthyroidism		Inverse variance weighted	6.338	4.125	565.483	0.174	1.84E+06	.124
		Median	0.435	2.843	NA	−5.138	6.007	.879
		MR-Egger	6.492	4.909	NA	−3.129	16.113	.186
HT		Inverse variance weighted	22.293	16.005	4.81E+09	0.000	2.02E+23	.164
		Median	10.797	11.389	NA	−11.525	33.120	.343
		MR-Egger	22.462	16.354	NA	−9.591	54.515	.170

AIH = autoimmune hepatitis, AILD = autoimmune liver disease, HT = Hashimoto thyroiditis, MVMR =multivariable Mendelian randomization, OR = odds ratio, PBC = primary biliary cholangitis, PSC = primary sclerosing cholangitis, SE = standard error.

First of all, AILD were treated as exposures and analyzed separately against 3 TD, hypothyroidism, hyperthyroidism, and HT, using MVMR. The limited number of SNPs in IVs, as per our screening criteria, precluded us from establishing a causal relationship between AIH and TD through multivariate MR. AIH showed a significant association with hypothyroidism (*P* < .001), hyperthyroidism (*P* = .008) across IVW method. The IVW method also revealed another significant causal relationship between PSC and hyperthyroidism (*P* < .001) and HT (*P* = .013).

Subsequently, we conducted multivariate MR analysis to investigate the association between TD as exposures and AILD as outcomes. The IVW method revealed a noteworthy causal association solely between HT and AIH (*P* = .035).

The causal relationship was found to be significant in the 2-sample MR analysis, but not in the MVMR analysis. This discrepancy may arise from potential unaccounted confounders in the 2-sample analysis, whereas MVMR accounted for these confounders by considering other relevant exposures and mediating effects. Moreover, MVMR is capable of identifying mediating effects where one variable influences the outcome through its influence on other variables. Additionally, as multiple exposures are considered in multivariate analyses, they can capture more complex interactions that might not have been fully captured in 2-sample analyses. Consequently, it is possible that the results obtained from the multivariate analysis appear inconsistent with those from the 2-sample analysis.

**Figure 5.**
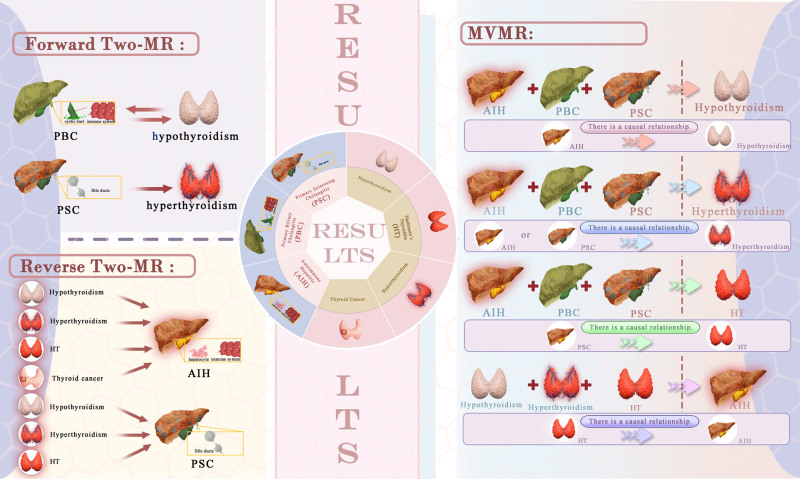
Visual summary of key causal associations identified by Mendelian randomization. AIH = autoimmune hepatitis, HT = Hashimoto thyroiditis, PBC = primary biliary cholangitis, PSC = primary sclerosing cholangitis.

To facilitate a clearer understanding of the principal causal relationships identified in our MR analyses, we present a comprehensive visual summary (Fig. 5).

## 4. Discussion

### 4.1. Discussion of the relationship between AIH and TD

In the forward MR analysis, no significant causal relationships were found between AIH and the 4 types of TD. However, the reverse MR analysis revealed significant causal associations between hypothyroidism, hyperthyroidism, HT, thyroid cancer, and AIH. Several factors may explain this discrepancy. First, while the genetic variants used in the forward analysis are strongly associated with the exposure, their direct effect on the outcome may be weak, resulting in nonsignificant findings. In contrast, the genetic variants in the reverse analysis have a stronger association with the outcome, leading to significant causal relationships. Additionally, differences in pleiotropy and statistical power could also contribute to the observed inconsistencies. The MR-Egger test indicated the presence of horizontal pleiotropy in the relationship between hyperthyroidism and AIH, suggesting that this causal association may be unstable and biased.

AITD, including HT and Graves’ disease, is a typical organ-specific autoimmune disorder.^[38,39]^ Yan et al’s epidemiological study found that among 41 AIH patients, 12 had concurrent HT and 6 had concurrent Graves’ disease, suggesting that AIH patients are at a significantly higher risk of developing TDs compared to those without AIH.^[40]^ Similarly, another study identified AITD as the most common complication in AIH patients.^[41]^ A cross-sectional study reported that 45.8% of AIH patients also developed AITD, with HT being the most prevalent (65.5%).^[42]^ Furthermore, a case-control study showed that AIH was independently associated with hypothyroidism, but not with hyperthyroidism.^[43]^ Additionally, 2 case reports have described HT in patients with AIH.^[44,45]^

### 4.2. Discussion of the relationship between PBC and TD

In the context of hyperthyroidism and PBC, we define PBC and hyperthyroidism as potential reciprocal causal factors in both MR and reverse MR analyses. However, PBC and HT were found to have a significant causal relationship in forward MR analysis, while the reverse analysis did not reveal a significant causal relationship.

Consistent with our findings, epidemiological studies emphasize the critical role of genetic factors in the pathogenesis of HT.^[46]^ One such study, examining the prevalence of extrahepatic autoimmune diseases in PBC patients, reported that among 1544 PBC patients, 141 (10.7%) also had HT.^[47]^ Another comparative study conducted across 2 European centers found that, out of 921 patients, 150 (16.3%) had thyroid disorders, with 94 (10.2%) diagnosed with HT. These results further support the strong association between AITD, particularly HT, and PBC.^[10]^ Additionally, an epidemiological study from Taiwan revealed that among extrahepatic outcomes in PBC patients, thyroid disorders had the highest prevalence at 24.4%, surpassing other autoimmune diseases.^[48]^ Based on these findings, clinical guidelines now recommend regular thyroid function screening for PBC patients to facilitate early detection and management of associated thyroid disorders.

The causal relationship between PBC and hypothyroidism can be analyzed from several perspectives. First, there is a complex interplay between thyroid hormones and liver function. Thyroid hormones regulate the basal metabolic rate of all cells, including hepatocytes, thus influencing liver function. Conversely, the liver plays a key role in metabolizing thyroid hormones and regulating their systemic endocrine effects.^[49,50]^ Disruption of thyroid function can impair liver function, while liver diseases, such as PBC, can alter thyroid hormone metabolism. PBC-induced liver damage can lead to changes in the expression of the enzyme D3, which regulates thyroid hormone activity. This alteration results in decreased accumulation of active thyroid hormones, triggering regulatory disturbances in the hypothalamic-pituitary-thyroid axis, leading to increased thyroid stimulating hormone (TSH) levels and, ultimately, hypothyroidism.^[51–54]^

Second, there is substantial evidence linking specific human leukocyte antigen (HLA) genotypes to the susceptibility of both PBC and AITD, including hypothyroidism. Notably, HLA-DRB108:01 and HLA-DRB108:03 have been identified as key genetic markers for PBC. While these genotypes are primarily associated with PBC, other HLA genotypes such as HLA-DR3 and HLA-DR5 are commonly observed in patients with AITD, including hypothyroidism and HT. These findings underscore the pivotal role of HLA genes in the pathogenesis of these autoimmune diseases and deepen our understanding of their shared pathophysiological mechanisms.^[55–57]^

Third, the relationship between PBC and hypothyroidism can be further understood through the role of Protein Y in thyroid hormone metabolism and liver function. Protein Y, which is abundantly expressed in the liver, facilitates the absorption and metabolism of thyroid hormones. In PBC patients, liver damage leads to reduced expression of Protein Y, impairing the liver’s ability to metabolize thyroid hormones effectively. This reduction in active thyroid hormones disrupts the hypothalamic-pituitary-thyroid axis, leading to elevated TSH levels and the development of hypothyroidism. This mechanism explains the higher incidence of hypothyroidism observed in PBC patients and highlights the interconnectedness between liver and thyroid function.^[58–60]^

Finally, in PBC patients, elevated levels of Th1 cytokines, particularly interferon-γ (IFN-γ), play a central role in the autoimmune response. IFN-γ activates macrophages and enhances antigen presentation by upregulating MHC class II molecules, thereby promoting autoimmune processes. The increased IFN-γ levels observed in PBC patients contribute to the destruction of biliary epithelial cells and the pathogenesis of AITD, including hypothyroidism. Studies have shown that hypothyroidism is significantly more prevalent in PBC patients than in the general population, suggesting a shared immunopathogenic mechanism. This connection is supported by evidence that IFN-γ exacerbates thyroid autoimmunity, leading to thyroid dysfunction and elevated TSH levels.^[61–63]^

### 4.3. Discussion of the relationship between PSC and TD

There is significant evidence of a causal relationship between PSC and hyperthyroidism, indicating that PSC may act as a potential risk factor for the development of hyperthyroid conditions. Furthermore, reverse MR analysis suggests that genetic susceptibility to hypothyroidism, hyperthyroidism, and HT may influence the risk of PSC; they may also potentially be a risk factor for PBC, as supported by a series of sensitivity analyses that corroborate the findings above.

The immunological mechanisms linking PSC and hyperthyroidism involve several key factors. Both diseases are autoimmune in nature, characterized by the immune system attacking the body’s own tissues. In PSC, the immune response targets the bile ducts, causing inflammation and fibrosis, with autoantibodies such as antinuclear antibodies and anti-smooth muscle antibodies commonly present. In Graves’ disease, autoantibodies, including thyroid-stimulating immunoglobulins, stimulate excessive thyroid hormone production. Shared genetic susceptibility, particularly certain HLA genotypes such as HLA-DR3 and HLA-DR4, indicates a common predisposition to immune dysregulation in both conditions.^[64,65]^

The connection between PSC and TD, particularly hyperthyroidism, can be explained through the gut microbiota. Dysbiosis, an imbalance in gut bacteria, is common in both PSC and TD. In PSC patients, changes in the gut microbiota, such as increased pathogenic bacteria like *Klebsiella* and *Bacteroides* and reduced beneficial bacteria, lead to intestinal barrier dysfunction. This dysfunction increases gut permeability, allowing microbial products to enter the bloodstream and trigger inflammation, which affects both the liver and thyroid.^[66,67]^ Similarly, TD, including Graves’ disease and HT, are associated with gut microbiota imbalances that promote autoimmune reactions by increasing gut permeability.^[68,69]^ The gut-liver-thyroid axis highlights the gut microbiota’s role in immune and metabolic regulation. Dysbiosis disrupts this axis, leading to inflammatory cytokine production (e.g., TNF-α, IL-1, IL-6), key to both PSC and TD pathogenesis.^[70]^ Additionally, gut microbiota influences the balance of T-cell subsets, including Treg and Th17 cells, which are crucial for immune tolerance. Dysregulation of these T-cell subsets is observed in both diseases, suggesting a shared immune pathway mediated by gut microbiota.^[71]^

### 4.4. Discussion of the relationship between hepatitis C and TD

The MR analysis found no significant causal association between CHC virus infection and TD. However, previous studies have reported a higher prevalence of hypothyroidism and thyroid autoimmunity in CHC patients, even without cirrhosis, hepatocellular carcinoma, or interferon therapy, compared to normal controls or individuals with chronic hepatitis B infection.^[18]^ A meta-analysis of 1735 chronic HCV-infected subjects who had not received IFN-α and 1868 controls showed a combined risk of hypothyroidism of 3.10 (95% CI 2.19–4.40), but no significant difference in the prevalence of hyperthyroidism.^[72]^ Additionally, a systematic review found that thyroid dysfunction occurred in 2.7% of HCV patients receiving interferon monotherapy, while the rate increased to 12.8% in those receiving combination therapy.^[73]^ IFN-α treatment is considered one of the most common causes of thyroid dysfunction in CHC patients, whereas newer DAAs have minimal impact on thyroid function. Moreover, evidence suggests that thyroid dysfunction may improve in patients cured of CHC with DAAs.^[74]^ The current understanding remains insufficient to determine whether TD in CHC patients results from the viral infection itself or from interferon therapy.^[21,75,76]^ Extensive documentation of TD coexistence in CHC patients suggests that interferon and ribavirin combination therapy, rather than viral load, plays a key role.^[77–80]^ Thyroid function should be promptly assessed before and during interferon treatment in CHC patients, with timely interventions if abnormalities are detected.

### 4.5. The strengths and limitations of this study

This study leverages high-quality, large-scale data from NHANES and GWAS, which enhances the robustness and reliability of our findings. The use of MR allows for effective control of confounding variables, thereby strengthening the causal inferences drawn from the data. By employing various MR methods such as IVW, MR-Egger, and the weighted median approach, we have ensured a thorough validation of our results. However, there are limitations to consider. The study population is predominantly of European descent, which may limit the applicability of our findings to other ethnic groups. Genetic variations and environmental factors that influence disease susceptibility may differ across populations, which could impact the generalizability of our results to non-European populations. While the GWAS data from European populations offer strong statistical power, future studies that include diverse populations from different racial and ethnic backgrounds are essential to verify the broader applicability of our findings. The validity of MR is heavily dependent on the selection of appropriate IVs, and any violations of MR assumptions, such as the presence of horizontal pleiotropy, could introduce bias. Additionally, the cross-sectional nature of NHANES data constrains our ability to infer temporal relationships and causality beyond genetic associations. Despite these limitations, our study provides valuable insights that warrant further investigation in more diverse populations and through longitudinal studies to confirm our findings.

## 5. Conclusion

This study elucidates the complex relationships between AILD, CHC, and TD using MR. Significant causal associations were identified between PBC and hypothyroidism, PSC and hyperthyroidism, and PBC and HT. These findings highlight the predisposition of individuals with AILD to thyroid dysfunction, underscoring the need for vigilant thyroid monitoring in these patients. Although no significant causal relationship was found between AIH and TD in the forward MR analysis, potential associations were indicated in the reverse MR analysis. This discrepancy suggests that while AIH may not directly cause TDs, there could be underlying genetic or environmental factors contributing to this relationship.

Additionally, no significant causal link was found between CHC and TD in the MR analysis, which contrasts with existing literature that reports higher thyroid dysfunction prevalence among HCV patients, especially those undergoing interferon therapy. This inconsistency underscores the complexity of HCV’s impact on thyroid health and highlights the importance of considering treatment effects separately from the disease itself.

These results emphasize the necessity of integrated care approaches for patients with these conditions, involving regular thyroid function monitoring and personalized management strategies. Further research, including large-scale clinical trials, is essential to validate these associations and to elucidate the underlying biological mechanisms. This will ultimately enhance our understanding and improve therapeutic strategies for managing the intersection of liver and TDs.

## Acknowledgments

The authors express their gratitude to the participants and coordinators of NHANES, MRC-IEU, the UK Biobank, and FinnGen Consortium for their pivotal role in facilitating this research through their exceptional dataset.

## Author contributions

**Conceptualization:** Qilong Nie, Yongwen Jiang.

**Data curation:** Qilong Nie, Yongwen Jiang, Qiuyan Liang.

**Formal analysis:** Qiuyan Liang, Caiyang Huang.

**Funding acquisition:** Jianhong Li.

**Investigation:** Mingyang Li.

**Methodology:** Yongwen Jiang, Mingyang Li.

**Project administration:** Yongwen Jiang, Yongwei Yuan, Tengyu Qiu.

**Resources:** Tengyu Qiu, Xiaojun Ma.

**Software:** Tengyu Qiu.

**Validation:** Caiyang Huang, Xiaojun Ma.

**Visualization:** Qilong Nie.

**Writing – original draft:** Qilong Nie.

**Writing – review & editing:** Jianhong Li.

## Supplementary Material


