# Supplementary material for: Exploring causal links between autoimmune liver diseases, chronic hepatitis C, and thyroid disorders: Evidence from NHANES and GWAS studies
Source: Medicine (Baltimore). 2025 Aug 29;104(35):e44112. doi: 10.1097/MD.0000000000044112 (PMC12401383; doi:10.1097/MD.0000000000044112)
Supplement: Supplementary file 1 [file medi-104-e44112-s001.pdf]

Table S1. Effect estimates of the associations between genetically predicted exposures and risk of TD

SNP	Chr	Position	Effect Allele	Other Allele	F	Association with exposure			Association with Hypothyroidism		
						Beta	SE	*P*	Beta	SE	*P*
**AIH**											
rs11139219	9	84023759	T	C	24.499	0.275	0.056	7.44E-07	0.000	0.001	0.49
rs115487918	19	53733101	T	G	22.270	0.489	0.104	2.38E-06	0.000	0.001	0.86
rs143112167	19	43579341	A	G	23.446	1.229	0.254	1.28E-06	0.001	0.002	0.48
rs1570330	1	168934842	C	T	24.650	0.255	0.051	7.03E-07	0.000	0.000	0.86
rs532543688	17	56268035	A	G	21.866	0.975	0.208	2.94E-06	0.000	0.001	0.85
rs57629035	19	15941100	T	C	23.135	0.263	0.055	1.50E-06	0.001	0.001	0.0790005
rs67967502	13	31577538	G	A	26.075	0.250	0.049	3.26E-07	0.000	0.000	0.86
rs7552246	1	244373475	C	T	21.389	0.258	0.056	3.68E-06	-0.001	0.001	0.0669993
rs77430417	4	184755725	C	G	22.646	0.882	0.185	1.95E-06	0.000	0.001	0.8
rs78388701	8	81677635	C	T	21.508	0.463	0.100	3.53E-06	-0.001	0.001	0.23
rs78753170	4	78167840	A	G	24.237	0.294	0.060	8.56E-07	-0.001	0.001	0.2
**PSC**											
rs10909839	1	2708430	A	G	30.082	-0.186	0.034	3.16E-08	-0.002	0.000	3.00E-07
rs1111463	6	31269926	C	A	410.063	-1.377	0.068	2.21E-101	0.008	0.001	2.70E-42
rs113198082	16	3881494	C	T	20.712	0.282	0.062	2.92E-06	0.000	0.001	0.56
rs114484678	6	32215057	C	T	29.652	-0.474	0.087	1.18E-08	-0.002	0.001	0.12
rs11632488	15	80267501	G	A	22.135	0.165	0.035	1.47E-06	-0.001	0.001	0.28
rs12956324	18	67537270	A	C	25.422	0.176	0.035	3.32E-07	0.002	0.000	6.30E-07
rs13119723	4	123218313	G	A	37.876	-0.271	0.044	2.22E-10	-0.002	0.001	0.00015
rs138763718	15	36686378	T	A	22.920	0.603	0.126	3.53E-07	0.001	0.002	0.649999
rs139458638	7	21872295	A	G	20.607	0.481	0.106	1.68E-06	0.000	0.002	0.79

rs145832854	22	25310129	A	G	27.526	-0.624	0.119	2.58E-08	0.001	0.002	0.36
rs145931087	1	192038977	C	T	20.855	0.365	0.080	2.29E-06	0.000	0.001	0.88
rs150464045	14	89834364	C	T	19.406	0.573	0.130	2.56E-06	-0.001	0.002	0.67
rs1893592	21	43855067	C	A	25.534	-0.202	0.040	1.90E-07	-0.002	0.000	2.70E-06
rs228616	4	103579691	A	G	23.226	-0.140	0.029	1.25E-06	0.001	0.000	0.0629999
rs231389	2	204634730	T	C	32.808	-0.206	0.036	4.42E-09	-0.002	0.001	0.000420001
rs2815037	6	39242453	G	A	29.142	-0.178	0.033	6.02E-08	0.001	0.000	0.00269998
rs3095227	6	31491000	G	A	43.005	0.230	0.035	3.58E-11	0.003	0.000	2.00E-09
rs3117012	6	33095684	G	A	23.072	-0.144	0.030	1.18E-06	0.002	0.000	0.000350002
rs3128931	6	32971708	A	G	41.731	-0.239	0.037	2.67E-11	0.000	0.001	0.42
rs3131781	6	30937732	G	A	835.569	1.070	0.037	1.00E-200	0.008	0.001	7.50E-34
rs313839	19	47221557	G	C	29.959	-0.279	0.051	2.12E-08	-0.001	0.001	0.08
rs3184504	12	111884608	C	T	38.639	-0.186	0.030	5.05E-10	-0.010	0.000	3.00E-116
rs34645399	6	32589169	G	A	252.396	0.747	0.047	1.63E-59	-0.008	0.001	1.50E-16
rs36023390	3	71523093	T	C	26.120	-0.184	0.036	2.16E-07	-0.001	0.000	0.23
rs4147359	10	6108439	A	G	52.188	0.217	0.030	4.06E-13	0.003	0.000	6.90E-12
rs428947	6	32234058	A	G	64.068	-0.632	0.079	5.62E-17	-0.004	0.001	1.70E-07
rs444697	6	33575009	A	G	27.196	0.177	0.034	1.07E-07	-0.001	0.001	0.15
rs453098	6	31691657	A	G	27.875	-0.338	0.064	4.71E-08	-0.001	0.001	0.58
rs4817988	21	40468838	A	G	58.957	-0.315	0.041	4.20E-15	0.000	0.000	1
rs492602	19	49206417	G	A	20.372	0.135	0.030	3.72E-06	0.002	0.000	5.00E-07
rs59377618	6	32788137	C	T	36.739	-0.303	0.050	4.39E-10	-0.003	0.001	4.20E-06
rs61954179	13	40794504	T	C	25.308	0.262	0.052	2.64E-07	0.000	0.001	0.68
rs62398260	6	31356234	A	C	61.215	-0.446	0.057	1.03E-15	-0.001	0.001	0.1
rs663743	11	64107735	A	G	27.580	-0.168	0.032	8.42E-08	-0.003	0.000	3.00E-10
rs687308	6	32567256	T	C	162.346	-0.624	0.049	2.44E-39	0.010	0.001	6.80E-67
rs725613	16	11169683	G	T	36.011	-0.198	0.033	5.50E-10	-0.001	0.000	0.11
rs72837826	2	111933001	T	G	35.484	0.304	0.051	1.26E-09	-0.002	0.001	0.0129999

rs73045256	19	41811072	G	A	22.650	0.338	0.071	7.13E-07	0.002	0.001	0.0690001
rs74655177	16	69098821	T	C	21.107	0.299	0.065	2.30E-06	0.000	0.001	0.649999
rs75030813	3	154810318	C	T	21.312	0.503	0.109	1.20E-06	0.002	0.001	0.17
rs7750271	6	91036225	G	A	24.652	0.213	0.043	3.06E-07	0.003	0.001	4.10E-08
rs7758790	6	31552850	C	T	133.403	-0.439	0.038	2.51E-32	0.000	0.001	0.52
rs7773324	6	382559	A	G	20.922	0.165	0.036	4.31E-06	0.003	0.000	6.80E-09
rs80060485	3	71153890	C	T	31.092	0.346	0.062	8.54E-09	0.002	0.001	0.0739997
rs850526	17	47285766	C	T	23.400	0.179	0.037	1.05E-06	0.000	0.000	0.86
rs9469323	6	32996802	T	C	35.852	0.222	0.037	1.50E-09	-0.003	0.001	1.40E-08
**PBC**											
rs10062349	5	141509597	A	G	28.967	0.120	0.022	7.36E-08	0.000	0.000	0.46
rs10137524	14	93017149	T	C	21.516	0.105	0.023	3.51E-06	0.000	0.000	0.66
rs10225558	7	138725878	T	C	29.552	-0.121	0.022	5.44E-08	-0.001	0.000	0.00680002
rs10398	11	308180	G	A	27.331	-0.147	0.028	1.71E-07	-0.001	0.001	0.00969996
rs1044044	4	100820850	T	C	23.708	-0.129	0.027	1.12E-06	-0.002	0.001	7.90E-05
rs10456404	6	32212857	C	T	49.284	-0.241	0.034	2.21E-12	0.000	0.001	0.98
rs10774625	12	111910219	G	A	79.271	-0.191	0.021	5.50E-19	-0.010	0.000	3.10E-111
rs10935453	3	141747640	G	T	22.453	0.110	0.023	2.15E-06	0.001	0.000	0.012
rs11117432	16	86019271	A	G	103.460	-0.273	0.027	2.82E-24	-0.003	0.001	1.40E-06
rs1119132	16	27403469	G	A	38.141	0.203	0.033	6.58E-10	0.001	0.001	0.0860003
rs1126511	6	33055501	T	G	452.905	0.516	0.024	4.91E-96	-0.006	0.001	6.20E-33
rs115079513	6	32820564	A	C	26.585	0.311	0.060	2.52E-07	-0.002	0.001	0.16
rs116906668	17	45371946	T	C	22.537	0.210	0.044	2.06E-06	0.003	0.001	0.002
rs11742240	5	35881376	T	G	78.952	-0.224	0.025	6.43E-19	-0.002	0.000	5.50E-06
rs11856301	15	79232330	C	T	26.468	0.119	0.023	2.68E-07	0.000	0.000	0.33
rs11912433	22	20884910	C	T	25.431	-0.131	0.026	4.58E-07	-0.001	0.001	0.015
rs12603276	17	34946547	C	T	21.491	-0.100	0.021	3.56E-06	0.001	0.000	0.22

rs12637633	3	160466737	A	G	23.677	-0.119	0.024	1.14E-06	-0.001	0.000	0.26
rs12706861	7	128616582	T	C	178.713	0.419	0.031	9.71E-41	0.004	0.001	5.70E-07
rs12712133	2	102866273	G	A	21.657	-0.101	0.022	3.26E-06	0.000	0.000	0.69
rs13053375	22	30752942	A	C	22.087	0.116	0.025	2.61E-06	0.001	0.001	0.025
rs13090881	3	159550129	C	T	73.999	0.197	0.023	7.92E-18	0.001	0.000	0.0179999
rs13238568	7	74076493	G	A	21.034	-0.100	0.022	4.51E-06	0.000	0.000	0.27
rs1330292	9	135884806	G	T	24.435	0.131	0.026	7.69E-07	0.001	0.001	0.31
rs137687	22	39740078	A	G	99.123	-0.218	0.022	2.37E-23	-0.002	0.000	2.50E-06
rs151102334	6	32743495	T	C	44.043	-0.350	0.053	3.21E-11	-0.004	0.001	1.00E-04
rs1582418	5	159882797	A	C	23.184	0.104	0.022	1.47E-06	0.000	0.000	0.46
rs16833239	2	191940260	A	G	34.051	-0.260	0.045	5.37E-09	-0.002	0.001	0.017
rs17214311	6	32988175	A	G	46.968	0.343	0.050	7.22E-12	0.003	0.001	0.0239999
rs1796357	12	42815553	C	T	26.201	0.168	0.033	3.08E-07	0.001	0.000	0.01
rs1800693	12	6440009	C	T	68.628	0.180	0.022	1.19E-16	0.000	0.000	0.760001
rs1808094	18	67533400	C	T	35.326	-0.128	0.021	2.79E-09	-0.003	0.000	1.10E-09
rs1811241	19	18235882	G	A	29.192	0.127	0.023	6.55E-08	0.001	0.000	0.012
rs210134	6	33540209	G	A	21.332	0.106	0.023	3.86E-06	0.000	0.000	0.47
rs2229092	6	31540757	C	A	44.217	0.279	0.042	2.94E-11	0.005	0.001	5.30E-08
rs2284033	22	37534034	A	G	23.690	-0.104	0.021	1.13E-06	-0.002	0.000	3.90E-05
rs2293370	3	119219934	A	G	106.756	-0.299	0.029	5.54E-25	-0.001	0.001	0.1
rs2304256	19	10475652	A	C	70.586	-0.205	0.024	4.43E-17	-0.003	0.000	2.70E-11
rs2305754	19	17179789	A	G	22.426	0.113	0.024	2.18E-06	0.000	0.000	0.33
rs2327832	6	137973068	G	A	40.189	0.161	0.025	2.31E-10	0.002	0.001	0.000309999
rs2428004	9	136806818	G	A	25.236	0.117	0.023	5.07E-07	0.001	0.000	0.0810009
rs243323	16	11361202	G	A	80.044	-0.214	0.024	3.71E-19	-0.001	0.000	0.0430002
rs2546890	5	158759900	G	A	44.530	-0.144	0.022	2.50E-11	-0.001	0.000	0.00409996
rs2732544	11	35092951	C	T	22.282	0.104	0.022	2.35E-06	0.000	0.000	0.5
rs2858829	6	116768917	G	A	21.040	-0.102	0.022	4.50E-06	0.001	0.000	0.0140001

rs30187	5	96124330	C	T	22.054	0.107	0.023	2.65E-06	0.001	0.000	0.27
rs35801	5	102608213	T	G	23.871	-0.114	0.023	1.03E-06	-0.003	0.000	4.10E-11
rs3745516	19	50926742	G	A	130.920	-0.274	0.024	2.65E-30	0.000	0.001	0.47
rs3771317	2	191543962	C	T	94.557	0.290	0.030	2.40E-22	0.002	0.001	0.000629999
rs3784099	14	68749927	A	G	69.400	-0.203	0.024	8.31E-17	-0.003	0.000	5.90E-11
rs3912060	17	44165435	T	C	41.601	-0.171	0.026	1.12E-10	0.001	0.001	0.0109999
rs4733851	8	129264420	G	A	26.863	-0.114	0.022	2.18E-07	0.001	0.000	0.00759994
rs4752829	11	47396654	A	G	23.963	0.138	0.028	9.82E-07	-0.001	0.000	0.29
rs4758501	11	2990693	T	G	21.804	-0.117	0.025	3.02E-06	0.000	0.001	0.4
rs485789	3	159730148	T	G	246.870	-0.354	0.023	1.96E-55	-0.002	0.000	2.60E-05
rs4936443	11	118740864	T	C	152.329	0.367	0.030	5.39E-35	0.000	0.001	0.69
rs4973334	2	228654079	C	T	21.161	0.100	0.022	4.22E-06	-0.001	0.000	0.17
rs55734382	1	201019059	T	C	35.913	-0.140	0.023	2.06E-09	-0.001	0.000	0.0025
rs59643720	14	103564807	C	A	167.441	0.316	0.024	2.73E-38	0.002	0.001	7.90E-05
rs59996420	6	162122039	G	A	26.445	0.432	0.084	2.71E-07	0.000	0.002	1
rs60600003	7	37382465	G	T	52.325	0.253	0.035	4.70E-13	0.004	0.001	2.00E-09
rs606252	13	51199462	A	C	26.561	-0.177	0.034	2.55E-07	-0.002	0.001	0.017
rs61839660	10	6094697	T	C	28.706	0.187	0.035	8.43E-08	-0.003	0.001	1.00E-04
rs62187044	2	225430410	T	C	23.322	-0.418	0.087	1.37E-06	-0.001	0.001	0.43
rs6478109	9	117568766	G	A	21.955	0.108	0.023	2.79E-06	0.000	0.000	0.85
rs6518350	21	45621817	G	A	26.657	-0.152	0.030	2.43E-07	0.000	0.001	0.94
rs6550965	3	25383587	A	C	57.349	0.163	0.022	3.65E-14	0.001	0.000	0.18
rs6679356	1	67820194	T	C	282.437	-0.439	0.026	6.61E-63	-0.003	0.001	4.20E-07
rs6848199	4	940838	T	C	28.190	0.118	0.022	1.10E-07	-0.001	0.000	0.0739997
rs7097397	10	50025396	A	G	40.095	-0.144	0.023	2.42E-10	-0.001	0.000	0.21
rs7130339	11	646232	A	G	29.852	0.122	0.022	4.66E-08	0.001	0.000	0.0061
rs72699866	14	93114787	A	G	44.250	-0.195	0.029	2.89E-11	-0.002	0.001	0.000219999
rs72808078	10	30814860	G	T	21.480	0.111	0.024	3.58E-06	0.000	0.000	0.93

rs728162	4	175076323	T	G	29.239	-0.120	0.022	6.40E-08	0.001	0.000	0.0479999
rs7317996	13	99805319	C	T	23.798	0.112	0.023	1.07E-06	0.001	0.000	0.00430002
rs7555082	1	198598663	A	G	23.053	-0.176	0.037	1.58E-06	-0.002	0.001	0.00109999
rs7663401	4	106128954	T	C	30.868	0.126	0.023	2.76E-08	-0.001	0.000	0.17
rs7674640	4	103540780	T	C	95.988	0.216	0.022	1.56E-22	0.000	0.000	0.86
rs7774434	6	32657578	C	T	471.948	0.470	0.022	3.68E-104	-0.002	0.000	2.40E-06
rs7805218	7	20378801	A	G	30.093	0.129	0.023	4.12E-08	0.002	0.000	0.000329997
rs78982396	6	32371652	A	C	179.982	0.530	0.040	5.41E-41	0.000	0.001	0.85
rs79577483	16	68036939	G	A	45.923	0.212	0.031	1.23E-11	0.002	0.001	0.0129999
rs8067378	17	38051348	G	A	145.479	0.260	0.022	1.75E-33	-0.001	0.000	0.0539995
rs859767	2	135341200	G	A	36.479	-0.139	0.023	1.54E-09	0.000	0.000	0.99
rs867436	1	2523723	T	C	35.193	0.134	0.023	2.99E-09	-0.003	0.000	5.70E-08
rs891058	2	8442547	A	G	25.117	-0.120	0.024	5.39E-07	-0.003	0.000	1.30E-08
rs9268517	6	32379740	T	C	33.293	0.292	0.051	7.93E-09	0.010	0.001	7.30E-16
rs9358913	6	26239404	G	A	28.521	0.130	0.024	9.27E-08	-0.003	0.001	5.70E-07
rs9533122	13	43055002	G	A	51.903	0.155	0.022	5.83E-13	0.003	0.000	9.00E-12
rs9591325	13	50811220	C	T	81.130	-0.452	0.050	2.14E-19	-0.003	0.001	0.000109999
rs9652601	16	11174365	A	G	101.718	-0.240	0.024	6.69E-24	-0.001	0.000	0.012
rs9810515	3	149184414	T	C	20.977	-0.110	0.024	4.65E-06	0.000	0.000	0.38
rs9843053	3	121617433	G	A	25.471	-0.132	0.026	4.49E-07	0.000	0.001	0.43
rs9853972	3	159877251	C	T	24.180	0.114	0.023	8.77E-07	0.001	0.000	0.0759994
rs9876137	3	16961265	G	A	42.242	0.144	0.022	8.06E-11	-0.001	0.000	0.08
**CHC**											
rs11776106	8	49260427	T	C	21.483	1.034	0.223	3.59E-06	-0.001	0.001	0.59
rs12613265	2	25329898	C	T	24.068	-0.083	0.017	9.67E-07	0.001	0.001	0.2
rs12927082	16	70810962	T	A	21.841	1.222	0.262	2.97E-06	-0.001	0.001	0.31
rs148302083	4	134395270	T	C	22.755	1.247	0.262	1.84E-06	0.000	0.001	0.82

rs189938683	20	18105918	A	G	21.310	1.979	0.429	3.91E-06	0.001	0.002	0.51
rs2052213	7	93865697	G	T	29.305	1.222	0.226	6.22E-08	-0.001	0.001	0.53
rs2212335	11	88748687	A	T	22.670	-0.116	0.024	1.90E-06	-0.001	0.001	0.48
rs29232	6	29611431	T	C	28.273	0.099	0.019	1.02E-07	-0.003	0.000	6.60E-12
rs3130506	6	31145451	A	G	23.733	0.110	0.023	1.08E-06	-0.004	0.000	1.80E-20
rs35800511	6	32636433	G	A	30.196	0.111	0.020	3.88E-08	0.006	0.001	9.20E-07
rs4778084	15	93603862	C	T	22.081	-0.102	0.022	2.70E-06	-0.001	0.000	0.18
rs62283057	4	6276805	T	C	21.407	0.568	0.123	3.72E-06	-0.002	0.001	0.00809991
rs62445574	7	14252711	G	A	21.443	0.416	0.090	3.67E-06	0.001	0.001	0.14
rs75918461	17	35111303	T	C	23.031	-0.108	0.022	1.65E-06	0.001	0.001	0.12
rs8113007	19	39743103	T	A	92.941	0.271	0.028	5.42E-22	0.000	0.000	0.6
rs9275267	6	32662676	G	A	49.080	-0.123	0.018	2.32E-12	0.005	0.000	1.10E-27
rs9501400	6	32394184	A	G	42.212	0.111	0.017	8.05E-11	-0.004	0.001	3.00E-11

**SNP**	**Chr**	**Position**	**Effect Allele**	**Other Allele**	**F**	**Association with exposure**			**Association with Hyperthyroidism**		
						**Beta**	**SE**	***P***	**Beta**	**SE**	***P***
**AIH**											
rs11139219	9	84023759	T	C	24.499	0.275	0.056	7.44E-07	0.000	0.000	0.31
rs115487918	19	53733101	T	G	22.270	0.489	0.104	2.38E-06	0.000	0.000	0.93
rs1570330	1	168934842	C	T	24.650	0.255	0.051	7.03E-07	0.000	0.000	0.85
rs57629035	19	15941100	T	C	23.135	0.263	0.055	1.50E-06	0.000	0.000	0.17
rs67967502	13	31577538	G	A	26.075	0.250	0.049	3.26E-07	0.000	0.000	0.64
rs7552246	1	244373475	C	T	21.389	0.258	0.056	3.68E-06	0.000	0.000	0.450001
rs78753170	4	78167840	A	G	24.237	0.294	0.060	8.56E-07	0.000	0.000	0.37
**PBC**											
rs10062349	5	141509597	A	G	28.967	0.120	0.022	7.36E-08	0.000	0.000	0.34

rs10137524	14	93017149	T	C	21.516	0.105	0.023	3.51E-06	0.000	0.000	0.11
rs10225558	7	138725878	T	C	29.552	-0.121	0.022	5.44E-08	0.000	0.000	0.61
rs10398	11	308180	G	A	27.331	-0.147	0.028	1.71E-07	-0.001	0.000	0.016
rs1044044	4	100820850	T	C	23.708	-0.129	0.027	1.12E-06	0.000	0.000	0.0519996
rs10456404	6	32212857	C	T	49.284	-0.241	0.034	2.21E-12	0.000	0.000	0.18
rs10774625	12	111910219	G	A	79.271	-0.191	0.021	5.50E-19	-0.001	0.000	3.10E-06
rs10935453	3	141747640	G	T	22.453	0.110	0.023	2.15E-06	0.000	0.000	0.2
rs11117432	16	86019271	A	G	103.460	-0.273	0.027	2.82E-24	-0.001	0.000	0.000420001
rs1119132	16	27403469	G	A	38.141	0.203	0.033	6.58E-10	0.000	0.000	0.8
rs1126511	6	33055501	T	G	452.905	0.516	0.024	4.91E-96	-0.001	0.000	5.40E-11
rs11742240	5	35881376	T	G	78.952	-0.224	0.025	6.43E-19	0.000	0.000	0.53
rs11856301	15	79232330	C	T	26.468	0.119	0.023	2.68E-07	0.000	0.000	0.86
rs11912433	22	20884910	C	T	25.431	-0.131	0.026	4.58E-07	0.000	0.000	0.760001
rs12603276	17	34946547	C	T	21.491	-0.100	0.021	3.56E-06	0.000	0.000	0.12
rs12637633	3	160466737	A	G	23.677	-0.119	0.024	1.14E-06	0.000	0.000	0.54
rs12706861	7	128616582	T	C	178.713	0.419	0.031	9.71E-41	0.000	0.000	0.46
rs12712133	2	102866273	G	A	21.657	-0.101	0.022	3.26E-06	0.000	0.000	0.0669993
rs13053375	22	30752942	A	C	22.087	0.116	0.025	2.61E-06	0.000	0.000	0.77
rs13090881	3	159550129	C	T	73.999	0.197	0.023	7.92E-18	0.000	0.000	0.0710003
rs13238568	7	74076493	G	A	21.034	-0.100	0.022	4.51E-06	0.000	0.000	0.18
rs1330292	9	135884806	G	T	24.435	0.131	0.026	7.69E-07	0.000	0.000	0.21
rs137687	22	39740078	A	G	99.123	-0.218	0.022	2.37E-23	0.000	0.000	0.28
rs1582418	5	159882797	A	C	23.184	0.104	0.022	1.47E-06	0.000	0.000	0.84
rs1796357	12	42815553	C	T	26.201	0.168	0.033	3.08E-07	0.000	0.000	0.18
rs1800693	12	6440009	C	T	68.628	0.180	0.022	1.19E-16	0.000	0.000	0.25
rs1808094	18	67533400	C	T	35.326	-0.128	0.021	2.79E-09	0.000	0.000	0.9
rs1811241	19	18235882	G	A	29.192	0.127	0.023	6.55E-08	0.000	0.000	0.91
rs210134	6	33540209	G	A	21.332	0.106	0.023	3.86E-06	0.000	0.000	0.015

rs2284033	22	37534034	A	G	23.690	-0.104	0.021	1.13E-06	0.000	0.000	0.0439997
rs2293370	3	119219934	A	G	106.756	-0.299	0.029	5.54E-25	0.000	0.000	0.15
rs2304256	19	10475652	A	C	70.586	-0.205	0.024	4.43E-17	0.000	0.000	0.025
rs2305754	19	17179789	A	G	22.426	0.113	0.024	2.18E-06	0.000	0.000	0.57
rs2327832	6	137973068	G	A	40.189	0.161	0.025	2.31E-10	0.001	0.000	0.000379997
rs2428004	9	136806818	G	A	25.236	0.117	0.023	5.07E-07	0.000	0.000	0.43
rs243323	16	11361202	G	A	80.044	-0.214	0.024	3.71E-19	0.000	0.000	0.44
rs2546890	5	158759900	G	A	44.530	-0.144	0.022	2.50E-11	0.000	0.000	0.630001
rs2732544	11	35092951	C	T	22.282	0.104	0.022	2.35E-06	0.000	0.000	0.74
rs2858829	6	116768917	G	A	21.040	-0.102	0.022	4.50E-06	0.000	0.000	0.93
rs3016014	6	31351153	A	G	77.153	0.215	0.024	1.59E-18	0.002	0.000	9.00E-21
rs30187	5	96124330	C	T	22.054	0.107	0.023	2.65E-06	0.000	0.000	0.0299999
rs3024495	1	206942413	T	C	22.318	0.134	0.028	2.31E-06	0.000	0.000	0.13
rs3093024	6	167532793	G	A	22.271	-0.102	0.022	2.37E-06	0.000	0.000	0.17
rs34655300	2	25514333	T	C	38.589	0.137	0.022	5.23E-10	0.000	0.000	0.62
rs35801	5	102608213	T	G	23.871	-0.114	0.023	1.03E-06	0.000	0.000	0.61
rs3771317	2	191543962	C	T	94.557	0.290	0.030	2.40E-22	0.000	0.000	0.22
rs4752829	11	47396654	A	G	23.963	0.138	0.028	9.82E-07	0.000	0.000	0.6
rs4758501	11	2990693	T	G	21.804	-0.117	0.025	3.02E-06	0.000	0.000	0.760001
rs485789	3	159730148	T	G	246.870	-0.354	0.023	1.96E-55	0.000	0.000	0.35
rs4936443	11	118740864	T	C	152.329	0.367	0.030	5.39E-35	0.000	0.000	0.21
rs4973334	2	228654079	C	T	21.161	0.100	0.022	4.22E-06	0.000	0.000	0.29
rs55734382	1	201019059	T	C	35.913	-0.140	0.023	2.06E-09	0.000	0.000	0.4
rs59643720	14	103564807	C	A	167.441	0.316	0.024	2.73E-38	0.000	0.000	0.760001
rs60600003	7	37382465	G	T	52.325	0.253	0.035	4.70E-13	0.000	0.000	0.98
rs606252	13	51199462	A	C	26.561	-0.177	0.034	2.55E-07	0.000	0.000	0.96
rs61839660	10	6097283	T	C	28.706	0.187	0.035	8.43E-08	-0.001	0.000	4.00E-05
rs6478109	9	117568766	G	A	21.955	0.108	0.023	2.79E-06	0.000	0.000	0.48

rs6518350	21	45621817	G	A	26.657	-0.152	0.030	2.43E-07	0.000	0.000	0.36
rs6550965	3	25383587	A	C	57.349	0.163	0.022	3.65E-14	0.000	0.000	0.95
rs6679356	1	67820194	T	C	282.437	-0.439	0.026	6.61E-63	0.000	0.000	0.36
rs6848199	4	940838	T	C	28.190	0.118	0.022	1.10E-07	0.000	0.000	0.86
rs7097397	10	50025396	A	G	40.095	-0.144	0.023	2.42E-10	0.000	0.000	0.81
rs7130339	11	646232	A	G	29.852	0.122	0.022	4.66E-08	0.000	0.000	0.69
rs72699866	14	93114787	A	G	44.250	-0.195	0.029	2.89E-11	0.000	0.000	0.97
rs72808078	10	30814860	G	T	21.480	0.111	0.024	3.58E-06	0.000	0.000	0.24
rs728162	4	175076323	T	G	29.239	-0.120	0.022	6.40E-08	-0.001	0.000	0.00530005
rs7317996	13	99805319	C	T	23.798	0.112	0.023	1.07E-06	0.000	0.000	0.95
rs7555082	1	198598663	A	G	23.053	-0.176	0.037	1.58E-06	-0.001	0.000	0.000109999
rs7663401	4	106128954	T	C	30.868	0.126	0.023	2.76E-08	0.000	0.000	0.73
rs7674640	4	103540780	T	C	95.988	0.216	0.022	1.56E-22	0.000	0.000	0.0140001
rs7774434	6	32657578	C	T	471.948	0.470	0.022	3.68E-104	-0.001	0.000	2.80E-13
rs7805218	7	20378801	A	G	30.093	0.129	0.023	4.12E-08	0.001	0.000	0.0051
rs79577483	16	68036939	G	A	45.923	0.212	0.031	1.23E-11	0.000	0.000	0.32
rs8067378	17	38051348	G	A	145.479	0.260	0.022	1.75E-33	0.000	0.000	0.13
rs859767	2	135341200	G	A	36.479	-0.139	0.023	1.54E-09	0.000	0.000	0.41
rs867436	1	2523723	T	C	35.193	0.134	0.023	2.99E-09	-0.001	0.000	0.0017
rs891058	2	8442547	A	G	25.117	-0.120	0.024	5.39E-07	0.000	0.000	0.51
rs9358913	6	26239404	G	A	28.521	0.130	0.024	9.27E-08	0.000	0.000	0.46
rs9533122	13	43055002	G	A	51.903	0.155	0.022	5.83E-13	0.000	0.000	0.0350002
rs9652601	16	11174365	A	G	101.718	-0.240	0.024	6.69E-24	0.000	0.000	0.61
rs9810515	3	149184414	T	C	20.977	-0.110	0.024	4.65E-06	0.000	0.000	0.709999
rs9843053	3	121617433	G	A	25.471	-0.132	0.026	4.49E-07	0.000	0.000	0.33
rs9853972	3	159877251	C	T	24.180	0.114	0.023	8.77E-07	0.000	0.000	0.52
rs9876137	3	16961265	G	A	42.242	0.144	0.022	8.06E-11	0.000	0.000	0.97

PSC

rs10909839	1	2708430	A	G	30.082	-0.186	0.034	3.16E-08	-0.001	0.000	0.00269998
rs1111463	6	31269926	C	A	410.063	-1.377	0.068	2.21E-101	-0.002	0.000	5.90E-14
rs11632488	15	80267501	G	A	22.135	0.165	0.035	1.47E-06	0.000	0.000	0.41
rs12956324	18	67537270	A	C	25.422	0.176	0.035	3.32E-07	0.000	0.000	0.15
rs13119723	4	123218313	G	A	37.876	-0.271	0.044	2.22E-10	0.000	0.000	0.98
rs1893592	21	43855067	C	A	25.534	-0.202	0.040	1.90E-07	-0.001	0.000	7.10E-05
rs228616	4	103579691	A	G	23.226	-0.140	0.029	1.25E-06	0.000	0.000	0.016
rs231389	2	204634730	T	C	32.808	-0.206	0.036	4.42E-09	-0.001	0.000	0.000700003
rs2815037	6	39242453	G	A	29.142	-0.178	0.033	6.02E-08	0.000	0.000	0.97
rs3095227	6	31491000	G	A	43.005	0.230	0.035	3.58E-11	0.002	0.000	4.90E-14
rs3117012	6	33095684	G	A	23.072	-0.144	0.030	1.18E-06	-0.001	0.000	1.50E-11
rs3128931	6	32971708	A	G	41.731	-0.239	0.037	2.67E-11	-0.001	0.000	7.20E-09
rs3131781	6	30937732	G	A	835.569	1.070	0.037	1.00E-200	0.005	0.000	3.70E-75
rs313839	19	47221557	G	C	29.959	-0.279	0.051	2.12E-08	0.000	0.000	0.709999
rs3184504	12	111884608	C	T	38.639	-0.186	0.030	5.05E-10	-0.001	0.000	7.30E-07
rs36023390	3	71523093	T	C	26.120	-0.184	0.036	2.16E-07	0.000	0.000	0.31
rs4147359	10	6108439	A	G	52.188	0.217	0.030	4.06E-13	0.001	0.000	3.20E-05
rs444697	6	33575009	A	G	27.196	0.177	0.034	1.07E-07	0.001	0.000	0.00980009
rs4817988	21	40468838	A	G	58.957	-0.315	0.041	4.20E-15	0.000	0.000	0.19
rs492602	19	49206417	G	A	20.372	0.135	0.030	3.72E-06	0.000	0.000	0.35
rs59377618	6	32788137	C	T	36.739	-0.303	0.050	4.39E-10	-0.001	0.000	0.012
rs663743	11	64107735	A	G	27.580	-0.168	0.032	8.42E-08	0.000	0.000	0.0219999
rs687308	6	32567256	T	C	162.346	-0.624	0.049	2.44E-39	-0.001	0.000	3.30E-06
rs725613	16	11169683	G	T	36.011	-0.198	0.033	5.50E-10	0.000	0.000	0.719999
rs72837826	2	111933001	T	G	35.484	0.304	0.051	1.26E-09	0.000	0.000	0.86
rs7750271	6	91036225	G	A	24.652	0.213	0.043	3.06E-07	0.000	0.000	0.061
rs7758790	6	31552850	C	T	133.403	-0.439	0.038	2.51E-32	-0.001	0.000	0.00189998
rs7773324	6	382559	A	G	20.922	0.165	0.036	4.31E-06	0.001	0.000	0.00530005

rs850526	17	47285766	C	T	23.400	0.179	0.037	1.05E-06	0.000	0.000	0.41
rs9461273	6	26584526	A	G	59.348	-0.231	0.030	3.92E-15	-0.001	0.000	0.00409996
rs9469323	6	32996802	T	C	35.852	0.222	0.037	1.50E-09	0.002	0.000	1.90E-12
rs9528775	13	65078624	T	C	21.138	0.166	0.036	2.66E-06	0.000	0.000	0.0810009
rs9553523	13	25630049	A	G	23.363	-0.222	0.046	6.21E-07	0.000	0.000	0.780001
rs9858213	3	49731861	T	G	83.737	0.284	0.031	2.43E-20	0.000	0.000	0.57
**CHC**											
rs12613265	2	25329898	C	T	24.068	-0.083	0.017	9.67E-07	0.000	0.000	0.22
rs29232	6	29611431	T	C	28.273	0.099	0.019	1.02E-07	-0.001	0.000	2.50E-11
rs3130506	6	31145451	A	G	23.733	0.110	0.023	1.08E-06	0.001	0.000	5.60E-06
rs4778084	15	93603862	C	T	22.081	-0.102	0.022	2.70E-06	0.000	0.000	0.46
rs62283057	4	6276805	T	C	21.407	0.568	0.123	3.72E-06	0.000	0.000	0.91
rs75918461	17	35111303	T	C	23.031	-0.108	0.022	1.65E-06	0.000	0.000	0.64
rs8113007	19	39743103	T	A	92.941	0.271	0.028	5.42E-22	0.000	0.000	0.79
rs9275267	6	32662676	G	A	49.080	-0.123	0.018	2.32E-12	-0.001	0.000	1.30E-12
rs9501400	6	32394184	A	G	42.212	0.111	0.017	8.05E-11	-0.002	0.000	1.80E-14

SNP	Chr	Position	Effect Allele	Other Allele	F	Association with exposure			Association with HT		
						Beta	SE	*P*	Beta	SE	*P*
**AIH**											
rs11139219	9	84023759	T	C	24.499	0.275	0.056	7.44E-07	0.010	0.019	0.5805
rs115487918	19	53729529	T	G	22.270	0.489	0.104	2.38E-06	0.112	0.113	0.3206
rs143112167	19	43579341	A	G	23.446	1.229	0.254	1.28E-06	0.132	0.087	0.1287
rs1570330	1	168934824	C	T	24.650	0.255	0.051	7.03E-07	-0.008	0.018	0.6369
rs1794514	6	32667473	C	G	56.317	0.501	0.067	6.09E-14	0.218	0.024	7.51E-20
rs192033796	3	60645112	T	C	22.351	0.754	0.159	2.28E-06	-0.066	0.044	0.1395

rs532543688	17	56266554	A	G	21.866	0.975	0.208	2.94E-06	-0.336	0.215	0.1186
rs57629035	19	15941100	T	C	23.135	0.263	0.055	1.50E-06	-0.008	0.019	0.672
rs67967502	13	31577538	G	A	26.075	0.250	0.049	3.26E-07	0.030	0.017	0.0814592
rs7552246	1	244373475	C	T	21.389	0.258	0.056	3.68E-06	0.020	0.019	0.2836
rs77430417	4	184755725	C	G	22.646	0.882	0.185	1.95E-06	0.058	0.063	0.3585
rs78388701	8	81677635	C	T	21.508	0.463	0.100	3.53E-06	-0.014	0.036	0.6998
rs78753170	4	78167840	A	G	24.237	0.294	0.060	8.56E-07	-0.017	0.021	0.4035
**PBC**											
rs10062349	5	141509597	A	G	28.967	0.120	0.022	7.36E-08	-0.002	0.017	0.9141
rs10137524	14	93017149	T	C	21.516	0.105	0.023	3.51E-06	-0.005	0.018	0.772999
rs10225558	7	138725878	T	C	29.552	-0.121	0.022	5.44E-08	0.002	0.016	0.8948
rs10398	11	308180	G	A	27.331	-0.147	0.028	1.71E-07	-0.047	0.021	0.0216098
rs1044044	4	100820850	T	C	23.708	-0.129	0.027	1.12E-06	-0.002	0.019	0.9117
rs10456404	6	32212857	C	T	49.284	-0.241	0.034	2.21E-12	0.001	0.030	0.965
rs10774625	12	111910219	G	A	79.271	-0.191	0.021	5.50E-19	-0.174	0.017	1.65E-24
rs10935453	3	141747640	G	T	22.453	0.110	0.023	2.15E-06	0.045	0.018	0.01136
rs11117432	16	86019271	A	G	103.460	-0.273	0.027	2.82E-24	-0.061	0.116	0.599601
rs1119132	16	27403469	G	A	38.141	0.203	0.033	6.58E-10	0.027	0.027	0.3192
rs1126511	6	33048466	T	G	452.905	0.516	0.024	4.91E-96	-0.098	0.022	9.39E-06
rs112729669	6	31410153	A	G	27.738	0.505	0.096	1.39E-07	0.336	0.520	0.5188
rs115079513	6	32820564	A	C	26.585	0.311	0.060	2.52E-07	0.030	0.046	0.5081
rs116906668	17	45371946	T	C	22.537	0.210	0.044	2.06E-06	0.133	0.052	0.01006
rs11742240	5	35881376	T	G	78.952	-0.224	0.025	6.43E-19	-0.029	0.018	0.0958606
rs11856301	15	79232330	C	T	26.468	0.119	0.023	2.68E-07	-0.011	0.017	0.5403
rs11912433	22	20875406	C	T	25.431	-0.131	0.026	4.58E-07	-0.049	0.054	0.3663
rs12603276	17	34946547	C	T	21.491	-0.100	0.021	3.56E-06	0.014	0.017	0.3987
rs12637633	3	160466737	A	G	23.677	-0.119	0.024	1.14E-06	-0.046	0.017	0.00747395
rs12706861	7	128616582	T	C	178.713	0.419	0.031	9.71E-41	0.022	0.024	0.3436

rs12712133	2	102866273	G	A	21.657	-0.101	0.022	3.26E-06	0.013	0.017	0.4335
rs13053375	22	30752942	A	C	22.087	0.116	0.025	2.61E-06	0.002	0.022	0.9304
rs13090881	3	159550129	C	T	73.999	0.197	0.023	7.92E-18	-0.020	0.019	0.3084
rs13238568	7	74076493	G	A	21.034	-0.100	0.022	4.51E-06	-0.025	0.018	0.1772
rs1330292	9	135884806	G	T	24.435	0.131	0.026	7.69E-07	-0.007	0.022	0.7662
rs137687	22	39740078	A	G	99.123	-0.218	0.022	2.37E-23	-0.011	0.017	0.5386
rs144007819	6	31855008	T	C	36.786	-0.688	0.113	1.32E-09	-0.043	0.094	0.647099
rs151102334	6	32743495	T	C	44.043	-0.350	0.053	3.21E-11	-0.021	0.035	0.5489
rs1582418	5	159882797	A	C	23.184	0.104	0.022	1.47E-06	-0.012	0.016	0.468
rs16833239	2	191940260	A	G	34.051	-0.260	0.045	5.37E-09	-0.058	0.034	0.08569
rs17214311	6	32988175	A	G	46.968	0.343	0.050	7.22E-12	-0.054	0.060	0.3689
rs1796357	12	42815553	C	T	26.201	0.168	0.033	3.08E-07	0.034	0.017	0.0433202
rs1800693	12	6440009	C	T	68.628	0.180	0.022	1.19E-16	0.005	0.017	0.750601
rs1808094	18	67526026	C	T	35.326	-0.128	0.021	2.79E-09	-0.009	0.052	0.8589
rs1811241	19	18235882	G	A	29.192	0.127	0.023	6.55E-08	0.019	0.018	0.2823
rs185453502	14	103135649	G	A	28.459	0.490	0.092	9.57E-08	0.124	0.426	0.7716
rs210134	6	33540209	G	A	21.332	0.106	0.023	3.86E-06	-0.009	0.020	0.6691
rs2229092	6	31540757	C	A	44.217	0.279	0.042	2.94E-11	-0.014	0.037	0.715
rs2284033	22	37534034	A	G	23.690	-0.104	0.021	1.13E-06	0.000	0.016	0.9866
rs2293370	3	119219934	A	G	106.756	-0.299	0.029	5.54E-25	-0.013	0.024	0.5903
rs2304256	19	10475652	A	C	70.586	-0.205	0.024	4.43E-17	-0.014	0.019	0.4424
rs2305754	19	17179789	A	G	22.426	0.113	0.024	2.18E-06	0.010	0.018	0.5866
rs2327832	6	137973068	G	A	40.189	0.161	0.025	2.31E-10	-0.058	0.115	0.611999
rs2428004	9	136806818	G	A	25.236	0.117	0.023	5.07E-07	-0.011	0.018	0.5299
rs243323	16	11361202	G	A	80.044	-0.214	0.024	3.71E-19	-0.012	0.018	0.5086
rs2732544	11	35092951	C	T	22.282	0.104	0.022	2.35E-06	-0.006	0.017	0.7089
rs3024495	1	206942413	T	C	22.318	0.134	0.028	2.31E-06	0.006	0.023	0.8089
rs3093024	6	167532793	G	A	22.271	-0.102	0.022	2.37E-06	-0.047	0.016	0.00404101

rs34655300	2	25514333	T	C	38.589	0.137	0.022	5.23E-10	0.031	0.017	0.0629796
rs35801	5	102608213	T	G	23.871	-0.114	0.023	1.03E-06	-0.047	0.018	0.00742301
rs3745516	19	50926742	G	A	130.920	-0.274	0.024	2.65E-30	-0.015	0.021	0.4694
rs3771317	2	191543962	C	T	94.557	0.290	0.030	2.40E-22	0.034	0.024	0.1647
rs3784099	14	68749927	A	G	69.400	-0.203	0.024	8.31E-17	-0.057	0.018	0.00171799
rs3912060	17	44165435	T	C	41.601	-0.171	0.026	1.12E-10	0.057	0.031	0.0646398
rs4733851	8	129264420	G	A	26.863	-0.114	0.022	2.18E-07	-0.039	0.018	0.02639
rs4752829	11	47396654	A	G	23.963	0.138	0.028	9.82E-07	-0.020	0.017	0.2557
rs4758501	11	2990693	T	G	21.804	-0.117	0.025	3.02E-06	-0.013	0.019	0.520701
rs485789	3	159730148	T	G	246.870	-0.354	0.023	1.96E-55	0.010	0.017	0.5572
rs4936443	11	118740864	T	C	152.329	0.367	0.030	5.39E-35	-0.004	0.022	0.8616
rs4973334	2	228654079	C	T	21.161	0.100	0.022	4.22E-06	-0.010	0.018	0.5938
rs55734382	1	201019059	T	C	35.913	-0.140	0.023	2.06E-09	-0.056	0.020	0.00431698
rs56119517	6	31847233	T	C	29.073	0.530	0.098	6.97E-08	0.325	0.222	0.1443
rs59643720	14	103564807	C	A	167.441	0.316	0.024	2.73E-38	0.036	0.019	0.0603893
rs59996420	6	162122039	G	A	26.445	0.432	0.084	2.71E-07	-0.041	0.077	0.591701
rs60600003	7	37382465	G	T	52.325	0.253	0.035	4.70E-13	0.032	0.028	0.2506
rs606252	13	51199462	A	C	26.561	-0.177	0.034	2.55E-07	0.001	0.027	0.9642
rs61839660	10	6094697	T	C	28.706	0.187	0.035	8.43E-08	0.016	0.042	0.701
rs62187044	2	225430410	T	C	23.322	-0.418	0.087	1.37E-06	-0.011	0.036	0.768999
rs6478109	9	117568766	G	A	21.955	0.108	0.023	2.79E-06	0.029	0.018	0.1002
rs6518350	21	45621817	G	A	26.657	-0.152	0.030	2.43E-07	-0.128	0.084	0.1247
rs6550965	3	25383587	A	C	57.349	0.163	0.022	3.65E-14	0.016	0.017	0.3422
rs6679356	1	67820194	T	C	282.437	-0.439	0.026	6.61E-63	-0.089	0.028	0.001523
rs6848199	4	940838	T	C	28.190	0.118	0.022	1.10E-07	0.041	0.098	0.677901
rs7097397	10	50025396	A	G	40.095	-0.144	0.023	2.42E-10	-0.060	0.017	0.000380198
rs7130339	11	646232	A	G	29.852	0.122	0.022	4.66E-08	-0.045	0.060	0.4491
rs72699866	14	93114787	A	G	44.250	-0.195	0.029	2.89E-11	-0.045	0.024	0.0668698

rs72808078	10	30814860	G	T	21.480	0.111	0.024	3.58E-06	0.070	0.105	0.5044
rs728162	4	175076323	T	G	29.239	-0.120	0.022	6.40E-08	0.002	0.017	0.8926
rs7287486	22	42322854	T	G	23.338	-0.475	0.098	1.36E-06	0.060	0.054	0.2624
rs73154557	3	160050257	A	G	22.254	0.689	0.146	2.39E-06	0.248	0.189	0.1899
rs7317996	13	99805319	C	T	23.798	0.112	0.023	1.07E-06	0.015	0.019	0.4489
rs7555082	1	198598663	A	G	23.053	-0.176	0.037	1.58E-06	-0.022	0.033	0.4983
rs7663401	4	106128954	T	C	30.868	0.126	0.023	2.76E-08	0.005	0.017	0.788301
rs7674640	4	103540780	T	C	95.988	0.216	0.022	1.56E-22	0.015	0.016	0.3494
rs7774434	6	32657578	C	T	471.948	0.470	0.022	3.68E-104	-0.024	0.020	0.2383
rs7805218	7	20378801	A	G	30.093	0.129	0.023	4.12E-08	-0.082	0.104	0.4297
rs78982396	6	32371652	A	C	179.982	0.530	0.040	5.41E-41	-0.083	0.023	0.000252203
rs79577483	16	68036939	G	A	45.923	0.212	0.031	1.23E-11	0.018	0.022	0.4263
rs8067378	17	38051348	G	A	145.479	0.260	0.022	1.75E-33	-0.030	0.017	0.0762009
rs859767	2	135341200	G	A	36.479	-0.139	0.023	1.54E-09	-0.027	0.017	0.1137
rs867436	1	2523723	T	C	35.193	0.134	0.023	2.99E-09	-0.079	0.099	0.427
rs891058	2	8442547	A	G	25.117	-0.120	0.024	5.39E-07	-0.045	0.017	0.01046
rs9268517	6	32379740	T	C	33.293	0.292	0.051	7.93E-09	0.148	0.063	0.0189802
rs9358913	6	26239404	G	A	28.521	0.130	0.024	9.27E-08	-0.044	0.017	0.01187
rs9533122	13	43055002	G	A	51.903	0.155	0.022	5.83E-13	0.041	0.017	0.0152598
rs9591325	13	50811220	C	T	81.130	-0.452	0.050	2.14E-19	-0.066	0.035	0.0606806
rs9652601	16	11174365	A	G	101.718	-0.240	0.024	6.69E-24	-0.039	0.018	0.0283903
rs9810515	3	149184414	T	C	20.977	-0.110	0.024	4.65E-06	-0.019	0.018	0.2774
rs9843053	3	121617433	G	A	25.471	-0.132	0.026	4.49E-07	-0.001	0.020	0.9538
rs9853972	3	159877251	C	T	24.180	0.114	0.023	8.77E-07	-0.013	0.019	0.4896
rs9876137	3	16961265	G	A	42.242	0.144	0.022	8.06E-11	-0.015	0.016	0.3478
**PSC**											
rs10909839	1	2708430	A	G	30.082	-0.186	0.034	3.16E-08	-0.024	0.017	0.1601
rs1111463	6	31269926	C	A	410.063	-1.377	0.068	2.21E-101	0.117	0.024	1.10E-06

rs113198082	16	3881494	C	T	20.712	0.282	0.062	2.92E-06	-0.056	0.034	0.0987393
rs114484678	6	32215057	C	T	29.652	-0.474	0.087	1.18E-08	-0.103	0.033	0.001622
rs11632488	15	80267501	G	A	22.135	0.165	0.035	1.47E-06	-0.022	0.020	0.2624
rs12956324	18	67537270	A	C	25.422	0.176	0.035	3.32E-07	0.021	0.017	0.2295
rs13119723	4	123218313	G	A	37.876	-0.271	0.044	2.22E-10	0.002	0.027	0.9334
rs138763718	15	36686378	T	A	22.920	0.603	0.126	3.53E-07	0.130	0.116	0.2616
rs139010734	6	31974014	T	C	574.410	3.355	0.140	1.98E-154	0.333	0.240	0.1656
rs139458638	7	21872295	A	G	20.607	0.481	0.106	1.68E-06	-0.261	0.106	0.01376
rs145832854	22	25310129	A	G	27.526	-0.624	0.119	2.58E-08	-0.116	0.096	0.2253
rs145931087	1	192038977	C	T	20.855	0.365	0.080	2.29E-06	-0.062	0.046	0.1808
rs150464045	14	89834364	C	T	19.406	0.573	0.130	2.56E-06	-0.040	0.116	0.726901
rs1893592	21	43855067	C	A	25.534	-0.202	0.040	1.90E-07	-0.054	0.018	0.00231398
rs228616	4	103579691	A	G	23.226	-0.140	0.029	1.25E-06	-0.016	0.016	0.3423
rs231389	2	204634730	T	C	32.808	-0.206	0.036	4.42E-09	-0.048	0.022	0.0257899
rs2815037	6	39242453	G	A	29.142	-0.178	0.033	6.02E-08	-0.016	0.018	0.3557
rs3095227	6	31491000	G	A	43.005	0.230	0.035	3.58E-11	-0.006	0.023	0.792399
rs3117012	6	33095684	G	A	23.072	-0.144	0.030	1.18E-06	0.040	0.017	0.0176202
rs3128931	6	32971708	A	G	41.731	-0.239	0.037	2.67E-11	0.021	0.021	0.3063
rs3131781	6	30937732	G	A	835.569	1.070	0.037	1.00E-200	0.170	0.033	2.43E-07
rs313839	19	47221557	G	C	29.959	-0.279	0.051	2.12E-08	-0.020	0.023	0.3965
rs3184504	12	111884608	C	T	38.639	-0.186	0.030	5.05E-10	-0.179	0.017	1.40E-25
rs34645399	6	32589169	G	A	252.396	0.747	0.047	1.63E-59	-0.105	0.031	0.000696194
rs36023390	3	71523093	T	C	26.120	-0.184	0.036	2.16E-07	-0.033	0.017	0.0566604
rs4147359	10	6108439	A	G	52.188	0.217	0.030	4.06E-13	0.034	0.017	0.0422396
rs428947	6	32195786	A	G	64.068	-0.632	0.079	5.62E-17	-0.127	0.085	0.1346
rs444697	6	33575009	A	G	27.196	0.177	0.034	1.07E-07	-0.002	0.021	0.9199
rs453098	6	31691657	A	G	27.875	-0.338	0.064	4.71E-08	-0.042	0.034	0.2148
rs4548024	6	138165744	C	T	27.706	-0.226	0.043	9.63E-08	-0.017	0.020	0.3995

rs4817988	21	40468838	A	G	58.957	-0.315	0.041	4.20E-15	0.023	0.019	0.2196
rs492602	19	49206417	G	A	20.372	0.135	0.030	3.72E-06	0.028	0.018	0.1162
rs59377618	6	32788137	C	T	36.739	-0.303	0.050	4.39E-10	-0.013	0.022	0.57
rs61954179	13	40794504	T	C	25.308	0.262	0.052	2.64E-07	-0.028	0.028	0.309
rs62398260	6	31356234	A	C	61.215	-0.446	0.057	1.03E-15	0.011	0.104	0.9144
rs663743	11	64107735	A	G	27.580	-0.168	0.032	8.42E-08	-0.040	0.017	0.02064
rs687308	6	32567256	T	C	162.346	-0.624	0.049	2.44E-39	0.163	0.024	8.63E-12
rs725613	16	11169683	G	T	36.011	-0.198	0.033	5.50E-10	-0.037	0.018	0.03359
rs72837826	2	111933001	T	G	35.484	0.304	0.051	1.26E-09	-0.003	0.028	0.9107
rs73045256	19	41811072	G	A	22.650	0.338	0.071	7.13E-07	0.049	0.030	0.1017
rs74407346	15	92060774	T	G	19.698	0.679	0.153	1.86E-06	0.208	0.186	0.2617
rs74655177	16	69098821	T	C	21.107	0.299	0.065	2.30E-06	-0.028	0.040	0.4866
rs75030813	3	154810318	C	T	21.312	0.503	0.109	1.20E-06	-0.155	0.117	0.1866
rs7750271	6	91036225	G	A	24.652	0.213	0.043	3.06E-07	0.091	0.026	0.000432703
rs7758790	6	31552850	C	T	133.403	-0.439	0.038	2.51E-32	-0.033	0.021	0.1166
rs79940565	11	63560994	C	T	27.321	0.763	0.146	2.00E-08	0.086	0.079	0.2767
rs9469323	6	32996802	T	C	35.852	0.222	0.037	1.50E-09	0.060	0.129	0.6392
rs9528775	13	65078624	T	C	21.138	0.166	0.036	2.66E-06	-0.041	0.018	0.0201902
rs9553523	13	25630049	A	G	23.363	-0.222	0.046	6.21E-07	0.021	0.025	0.4029
rs9858213	3	49731861	T	G	83.737	0.284	0.031	2.43E-20	-0.007	0.017	0.690501
**CHC**											
rs11776106	8	49260427	T	C	21.483	1.034	0.223	3.59E-06	0.062	0.051	0.2238
rs12613265	2	25329898	C	T	24.068	-0.083	0.017	9.67E-07	-0.018	0.019	0.3438
rs12927082	16	70810962	T	A	21.841	1.222	0.262	2.97E-06	-0.020	0.048	0.6821
rs143003569	17	76540839	T	C	21.991	-0.103	0.022	2.67E-06	0.051	0.069	0.4603
rs148302083	4	134395270	T	C	22.755	1.247	0.262	1.84E-06	0.029	0.058	0.6133
rs149513365	2	231646702	T	C	23.766	3.621	0.743	1.09E-06	-0.211	0.288	0.4642
rs189938683	20	18105918	A	G	21.310	1.979	0.429	3.91E-06	-0.008	0.047	0.8651

rs2052213	7	93865697	G	T	29.305	1.222	0.226	6.22E-08	-0.129	0.046	0.00472498
rs2212335	11	88748687	A	T	22.670	-0.116	0.024	1.90E-06	-0.019	0.032	0.542
rs29232	6	29611431	T	C	28.273	0.099	0.019	1.02E-07	-0.045	0.019	0.0169399
rs3130506	6	31145451	A	G	23.733	0.110	0.023	1.08E-06	-0.058	0.021	0.00644303
rs35800511	6	32636433	G	A	30.196	0.111	0.020	3.88E-08	0.169	0.044	0.000132801
rs4778084	15	93603862	C	T	22.081	-0.102	0.022	2.70E-06	0.012	0.017	0.4717
rs550678256	14	45385952	T	A	21.017	1.333	0.291	4.55E-06	-0.346	0.294	0.2389
rs62283057	4	6276805	T	C	21.407	0.568	0.123	3.72E-06	-0.037	0.022	0.0904107
rs62445574	7	14252711	G	A	21.443	0.416	0.090	3.67E-06	-0.003	0.028	0.9071
rs75918461	17	35111303	T	C	23.031	-0.108	0.022	1.65E-06	0.035	0.019	0.0625403
rs8113007	19	39743103	T	A	92.941	0.271	0.028	5.42E-22	0.005	0.020	0.8048
rs9275267	6	32662425	G	A	49.080	-0.123	0.018	2.32E-12	0.116	0.053	0.0285299
rs9501400	6	32394184	A	G	42.212	0.111	0.017	8.05E-11	-0.089	0.025	0.0003491

SNP	Chr	Position	Effect Allele	Other Allele	F	Association with exposure			Association with Thyroid cancer		
						**Beta**	**SE**	***P***	**Beta**	**SE**	***P***
**AIH**											
rs11139219	9	84023759	T	C	24.499	0.275	0.056	7.44E-07	0.275	0.044	0.2204
rs115487918	19	53729529	T	G	22.270	0.489	0.104	2.38E-06	0.489	0.071	0.2765
rs143112167	19	43579341	A	G	23.446	1.229	0.254	1.28E-06	1.229	0.201	0.4609
rs1570330	1	168934824	C	T	24.650	0.255	0.051	7.03E-07	0.255	0.042	0.1244
rs1794514	6	32667473	C	G	56.317	0.501	0.067	6.09E-14	0.501	0.054	0.2147
rs192033796	3	60645112	T	C	22.351	0.754	0.159	2.28E-06	0.754	0.156	0.3081
rs532543688	17	56266554	A	G	21.866	0.975	0.208	2.94E-06	0.975	0.135	0.4878
rs57629035	19	15941100	T	C	23.135	0.263	0.055	1.50E-06	0.263	0.044	0.9814
rs67967502	13	31577538	G	A	26.075	0.250	0.049	3.26E-07	0.250	0.039	0.3592

rs7552246	1	244373475	C	T	21.389	0.258	0.056	3.68E-06	0.258	0.046	0.318
rs77430417	4	184755725	C	G	22.646	0.882	0.185	1.95E-06	0.882	0.150	0.1006
rs78388701	8	81677635	C	T	21.508	0.463	0.100	3.53E-06	0.463	0.085	0.9255
rs78753170	4	78167840	A	G	24.237	0.294	0.060	8.56E-07	0.294	0.045	0.1064
**PBC**											
rs10062349	5	141509597	A	G	28.967	0.120	0.022	7.36E-08	0.006	0.039	0.8863
rs10137524	14	93017149	T	C	21.516	0.105	0.023	3.51E-06	-0.033	0.046	0.4722
rs10225558	7	138725878	T	C	29.552	-0.121	0.022	5.44E-08	0.067	0.039	0.0816507
rs10398	11	308180	G	A	27.331	-0.147	0.028	1.71E-07	0.088	0.046	0.0574394
rs1044044	4	100820850	T	C	23.708	-0.129	0.027	1.12E-06	-0.021	0.045	0.641501
rs10456404	6	32212857	C	T	49.284	-0.241	0.034	2.21E-12	-0.082	0.067	0.2181
rs10774625	12	111910219	G	A	79.271	-0.191	0.021	5.50E-19	0.054	0.044	0.2176
rs10935453	3	141747640	G	T	22.453	0.110	0.023	2.15E-06	-0.026	0.041	0.5223
rs11117432	16	86019271	A	G	103.460	-0.273	0.027	2.82E-24	-0.076	0.073	0.2981
rs1119132	16	27403469	G	A	38.141	0.203	0.033	6.58E-10	0.074	0.055	0.1794
rs1126511	6	33048466	T	G	452.905	0.516	0.024	4.91E-96	-0.100	0.048	0.0377303
rs112729669	6	31410153	A	G	27.738	0.505	0.096	1.39E-07	-0.080	0.325	0.8055
rs115079513	6	32820564	A	C	26.585	0.311	0.060	2.52E-07	-0.004	0.127	0.9726
rs116906668	17	45371946	T	C	22.537	0.210	0.044	2.06E-06	0.007	0.110	0.9481
rs11742240	5	35881376	T	G	78.952	-0.224	0.025	6.43E-19	-0.018	0.042	0.663001
rs11856301	15	79232330	C	T	26.468	0.119	0.023	2.68E-07	-0.055	0.043	0.1978
rs11912433	22	20875406	C	T	25.431	-0.131	0.026	4.58E-07	-0.013	0.052	0.8003
rs12603276	17	34946547	C	T	21.491	-0.100	0.021	3.56E-06	-0.042	0.038	0.2692
rs12637633	3	160466737	A	G	23.677	-0.119	0.024	1.14E-06	-0.054	0.040	0.1739
rs12706861	7	128616582	T	C	178.713	0.419	0.031	9.71E-41	0.088	0.065	0.1744
rs12712133	2	102866273	G	A	21.657	-0.101	0.022	3.26E-06	0.024	0.040	0.5439
rs13053375	22	30752942	A	C	22.087	0.116	0.025	2.61E-06	-0.067	0.051	0.184
rs13090881	3	159550129	C	T	73.999	0.197	0.023	7.92E-18	-0.017	0.043	0.699199

rs13238568	7	74076493	G	A	21.034	-0.100	0.022	4.51E-06	0.001	0.044	0.9794
rs1330292	9	135884806	G	T	24.435	0.131	0.026	7.69E-07	-0.052	0.054	0.3382
rs137687	22	39740078	A	G	99.123	-0.218	0.022	2.37E-23	0.043	0.043	0.3227
rs144007819	6	31855008	T	C	36.786	-0.688	0.113	1.32E-09	0.248	0.218	0.2556
rs151102334	6	32743495	T	C	44.043	-0.350	0.053	3.21E-11	0.182	0.086	0.0341499
rs1582418	5	159882797	A	C	23.184	0.104	0.022	1.47E-06	0.021	0.038	0.5872
rs16833239	2	191940260	A	G	34.051	-0.260	0.045	5.37E-09	0.016	0.066	0.8062
rs17214311	6	32988175	A	G	46.968	0.343	0.050	7.22E-12	0.024	0.105	0.8192
rs1796357	12	42815553	C	T	26.201	0.168	0.033	3.08E-07	-0.044	0.040	0.2696
rs1800693	12	6440009	C	T	68.628	0.180	0.022	1.19E-16	-0.063	0.041	0.12
rs1808094	18	67526026	C	T	35.326	-0.128	0.021	2.79E-09	0.054	0.047	0.2521
rs1811241	19	18235882	G	A	29.192	0.127	0.023	6.55E-08	-0.028	0.043	0.517201
rs185453502	14	103135649	G	A	28.459	0.490	0.092	9.57E-08	-0.190	0.270	0.4821
rs210134	6	33540209	G	A	21.332	0.106	0.023	3.86E-06	-0.063	0.043	0.1455
rs2229092	6	31540757	C	A	44.217	0.279	0.042	2.94E-11	0.113	0.088	0.2023
rs2284033	22	37534034	A	G	23.690	-0.104	0.021	1.13E-06	0.030	0.038	0.4303
rs2293370	3	119219934	A	G	106.756	-0.299	0.029	5.54E-25	0.083	0.048	0.0850903
rs2304256	19	10475652	A	C	70.586	-0.205	0.024	4.43E-17	-0.093	0.042	0.02558
rs2305754	19	17179789	A	G	22.426	0.113	0.024	2.18E-06	-0.042	0.040	0.3011
rs2327832	6	137973068	G	A	40.189	0.161	0.025	2.31E-10	-0.130	0.072	0.0734598
rs2428004	9	136806818	G	A	25.236	0.117	0.023	5.07E-07	-0.014	0.041	0.742899
rs243323	16	11361202	G	A	80.044	-0.214	0.024	3.71E-19	-0.096	0.043	0.0244399
rs2732544	11	35092951	C	T	22.282	0.104	0.022	2.35E-06	0.100	0.039	0.01086
rs3024495	1	206942413	T	C	22.318	0.134	0.028	2.31E-06	0.021	0.059	0.7302
rs3093024	6	167532793	G	A	22.271	-0.102	0.022	2.37E-06	-0.003	0.038	0.9461
rs34655300	2	25514333	T	C	38.589	0.137	0.022	5.23E-10	-0.036	0.041	0.3764
rs35801	5	102608213	T	G	23.871	-0.114	0.023	1.03E-06	0.038	0.041	0.3523
rs3745516	19	50926742	G	A	130.920	-0.274	0.024	2.65E-30	-0.006	0.048	0.8948

rs3771317	2	191543962	C	T	94.557	0.290	0.030	2.40E-22	0.075	0.050	0.1342
rs3784099	14	68749927	A	G	69.400	-0.203	0.024	8.31E-17	-0.075	0.044	0.0898504
rs3912060	17	44165435	T	C	41.601	-0.171	0.026	1.12E-10	0.081	0.061	0.1901
rs4733851	8	129264420	G	A	26.863	-0.114	0.022	2.18E-07	0.020	0.042	0.6223
rs4752829	11	47396654	A	G	23.963	0.138	0.028	9.82E-07	-0.012	0.041	0.763999
rs4758501	11	2990693	T	G	21.804	-0.117	0.025	3.02E-06	-0.038	0.048	0.437
rs485789	3	159730148	T	G	246.870	-0.354	0.023	1.96E-55	-0.008	0.043	0.8549
rs4936443	11	118740864	T	C	152.329	0.367	0.030	5.39E-35	-0.050	0.052	0.3391
rs4973334	2	228654079	C	T	21.161	0.100	0.022	4.22E-06	-0.022	0.040	0.5857
rs55734382	1	201019059	T	C	35.913	-0.140	0.023	2.06E-09	-0.093	0.047	0.0510799
rs56119517	6	31847233	T	C	29.073	0.530	0.098	6.97E-08	-0.221	0.315	0.4828
rs59643720	14	103564807	C	A	167.441	0.316	0.024	2.73E-38	0.030	0.044	0.502
rs59996420	6	162122039	G	A	26.445	0.432	0.084	2.71E-07	0.098	0.175	0.5761
rs60600003	7	37382465	G	T	52.325	0.253	0.035	4.70E-13	-0.127	0.073	0.0826399
rs606252	13	51199462	A	C	26.561	-0.177	0.034	2.55E-07	-0.034	0.063	0.5898
rs61839660	10	6094697	T	C	28.706	0.187	0.035	8.43E-08	-0.143	0.085	0.0945693
rs62187044	2	225430410	T	C	23.322	-0.418	0.087	1.37E-06	0.101	0.097	0.2964
rs6478109	9	117568766	G	A	21.955	0.108	0.023	2.79E-06	0.067	0.040	0.0944104
rs6518350	21	45621817	G	A	26.657	-0.152	0.030	2.43E-07	0.031	0.067	0.6428
rs6550965	3	25383587	A	C	57.349	0.163	0.022	3.65E-14	0.028	0.040	0.4801
rs6679356	1	67820194	T	C	282.437	-0.439	0.026	6.61E-63	-0.100	0.063	0.1117
rs6848199	4	940838	T	C	28.190	0.118	0.022	1.10E-07	0.039	0.062	0.535499
rs7097397	10	50025396	A	G	40.095	-0.144	0.023	2.42E-10	-0.030	0.039	0.4504
rs7130339	11	646232	A	G	29.852	0.122	0.022	4.66E-08	-0.035	0.051	0.4901
rs72699866	14	93114787	A	G	44.250	-0.195	0.029	2.89E-11	0.020	0.059	0.7317
rs72808078	10	30814860	G	T	21.480	0.111	0.024	3.58E-06	-0.082	0.066	0.2178
rs728162	4	175076323	T	G	29.239	-0.120	0.022	6.40E-08	-0.020	0.041	0.6242
rs7287486	22	42322854	T	G	23.338	-0.475	0.098	1.36E-06	0.096	0.123	0.4352

rs73154557	3	160050257	A	G	22.254	0.689	0.146	2.39E-06	0.363	0.418	0.3844
rs7317996	13	99805319	C	T	23.798	0.112	0.023	1.07E-06	-0.061	0.045	0.18
rs7555082	1	198598663	A	G	23.053	-0.176	0.037	1.58E-06	-0.095	0.075	0.2025
rs7663401	4	106128954	T	C	30.868	0.126	0.023	2.76E-08	-0.015	0.039	0.698499
rs7674640	4	103540780	T	C	95.988	0.216	0.022	1.56E-22	-0.003	0.038	0.9435
rs7774434	6	32657578	C	T	471.948	0.470	0.022	3.68E-104	-0.023	0.041	0.5825
rs7805218	7	20378801	A	G	30.093	0.129	0.023	4.12E-08	0.001	0.065	0.9865
rs78982396	6	32371652	A	C	179.982	0.530	0.040	5.41E-41	0.017	0.059	0.773101
rs79577483	16	68036939	G	A	45.923	0.212	0.031	1.23E-11	-0.060	0.054	0.2695
rs8067378	17	38051348	G	A	145.479	0.260	0.022	1.75E-33	-0.067	0.039	0.0852806
rs859767	2	135341200	G	A	36.479	-0.139	0.023	1.54E-09	0.024	0.040	0.549
rs867436	1	2523723	T	C	35.193	0.134	0.023	2.99E-09	0.059	0.063	0.3493
rs891058	2	8442547	A	G	25.117	-0.120	0.024	5.39E-07	-0.018	0.041	0.666699
rs9268517	6	32379740	T	C	33.293	0.292	0.051	7.93E-09	-0.103	0.110	0.3504
rs9358913	6	26239404	G	A	28.521	0.130	0.024	9.27E-08	-0.021	0.044	0.624499
rs9533122	13	43055002	G	A	51.903	0.155	0.022	5.83E-13	0.000	0.040	0.9936
rs9591325	13	50811220	C	T	81.130	-0.452	0.050	2.14E-19	0.030	0.090	0.7392
rs9652601	16	11174365	A	G	101.718	-0.240	0.024	6.69E-24	-0.040	0.043	0.3484
rs9810515	3	149184414	T	C	20.977	-0.110	0.024	4.65E-06	0.109	0.046	0.0181501
rs9843053	3	121617433	G	A	25.471	-0.132	0.026	4.49E-07	-0.044	0.047	0.3544
rs9853972	3	159877251	C	T	24.180	0.114	0.023	8.77E-07	-0.021	0.042	0.6108
rs9876137	3	16961265	G	A	42.242	0.144	0.022	8.06E-11	0.004	0.039	0.9241
**PSC**											
rs10909839	1	2708430	A	G	30.082	-0.186	0.039	0.8065	0.010	0.034	3.16E-08
rs1111463	6	31269926	C	A	410.063	-1.377	0.049	0.2712	-0.054	0.068	2.21E-101
rs113198082	16	3881494	C	T	20.712	0.282	0.086	0.6555	-0.038	0.062	2.92E-06
rs114484678	6	32215057	C	T	29.652	-0.474	0.076	0.7563	0.024	0.087	1.18E-08
rs11632488	15	80267501	G	A	22.135	0.165	0.045	0.4798	-0.032	0.035	1.47E-06

rs12956324	18	67537270	A	C	25.422	0.176	0.040	0.6782	-0.017	0.035	3.32E-07
rs13119723	4	123218313	G	A	37.876	-0.271	0.062	0.0219801	-0.143	0.044	2.22E-10
rs138763718	15	36686378	T	A	22.920	0.603	0.205	0.3715	0.183	0.126	3.53E-07
rs139010734	6	31974014	T	C	574.410	3.355	0.848	0.1888	1.115	0.140	1.98E-154
rs139458638	7	21872295	A	G	20.607	0.481	0.199	0.2112	-0.249	0.106	1.68E-06
rs145832854	22	25310129	A	G	27.526	-0.624	0.174	0.793201	0.046	0.119	2.58E-08
rs145931087	1	192038977	C	T	20.855	0.365	0.128	0.543	-0.078	0.080	2.29E-06
rs150464045	14	89834364	C	T	19.406	0.573	0.202	0.576901	-0.113	0.130	2.56E-06
rs1893592	21	43855067	C	A	25.534	-0.202	0.042	0.02773	0.092	0.040	1.90E-07
rs228616	4	103579691	A	G	23.226	-0.140	0.038	0.8293	-0.008	0.029	1.25E-06
rs231389	2	204634730	T	C	32.808	-0.206	0.051	0.9223	0.005	0.036	4.42E-09
rs2815037	6	39242453	G	A	29.142	-0.178	0.040	0.2544	-0.046	0.033	6.02E-08
rs3095227	6	31491000	G	A	43.005	0.230	0.050	0.7474	0.016	0.035	3.58E-11
rs3117012	6	33095684	G	A	23.072	-0.144	0.039	0.5611	-0.023	0.030	1.18E-06
rs3128931	6	32971708	A	G	41.731	-0.239	0.045	0.8416	-0.009	0.037	2.67E-11
rs3131781	6	30937732	G	A	835.569	1.070	0.071	0.8806	0.011	0.037	1.00E-200
rs313839	19	47221557	G	C	29.959	-0.279	0.052	0.5538	0.031	0.051	2.12E-08
rs3184504	12	111884608	C	T	38.639	-0.186	0.044	0.0986893	0.073	0.030	5.05E-10
rs34645399	6	32589169	G	A	252.396	0.747	0.088	0.3135	0.089	0.047	1.63E-59
rs36023390	3	71523093	T	C	26.120	-0.184	0.045	0.1004	-0.074	0.036	2.16E-07
rs4147359	10	6108439	A	G	52.188	0.217	0.039	0.4484	0.030	0.030	4.06E-13
rs428947	6	32195786	A	G	64.068	-0.632	0.107	0.02857	-0.234	0.079	5.62E-17
rs444697	6	33575009	A	G	27.196	0.177	0.044	0.4938	-0.030	0.034	1.07E-07
rs453098	6	31691657	A	G	27.875	-0.338	0.090	0.01348	-0.221	0.064	4.71E-08
rs4548024	6	138165744	C	T	27.706	-0.226	0.048	0.3395	0.046	0.043	9.63E-08
rs4817988	21	40468838	A	G	58.957	-0.315	0.045	0.737801	0.015	0.041	4.20E-15
rs492602	19	49206417	G	A	20.372	0.135	0.045	0.5039	0.030	0.030	3.72E-06
rs59377618	6	32788137	C	T	36.739	-0.303	0.053	0.9654	0.002	0.050	4.39E-10

rs61954179	13	40794504	T	C	25.308	0.262	0.070	0.4888	-0.048	0.052	2.64E-07
rs62398260	6	31356234	A	C	61.215	-0.446	0.091	0.0122301	-0.228	0.057	1.03E-15
rs663743	11	64107735	A	G	27.580	-0.168	0.041	0.1944	-0.053	0.032	8.42E-08
rs687308	6	32567256	T	C	162.346	-0.624	0.050	0.3284	-0.048	0.049	2.44E-39
rs725613	16	11169683	G	T	36.011	-0.198	0.042	0.5019	-0.028	0.033	5.50E-10
rs72837826	2	111933001	T	G	35.484	0.304	0.072	0.1975	0.092	0.051	1.26E-09
rs73045256	19	41811072	G	A	22.650	0.338	0.085	0.6536	-0.038	0.071	7.13E-07
rs74407346	15	92060774	T	G	19.698	0.679	0.268	0.8565	-0.048	0.153	1.86E-06
rs74655177	16	69098821	T	C	21.107	0.299	0.101	0.9527	-0.006	0.065	2.30E-06
rs75030813	3	154810318	C	T	21.312	0.503	0.182	0.6995	0.070	0.109	1.20E-06
rs7750271	6	91036225	G	A	24.652	0.213	0.061	0.9777	-0.002	0.043	3.06E-07
rs7758790	6	31552850	C	T	133.403	-0.439	0.049	0.0866004	0.084	0.038	2.51E-32
rs79940565	11	63560994	C	T	27.321	0.763	0.210	0.3343	-0.203	0.146	2.00E-08
rs9469323	6	32996802	T	C	35.852	0.222	0.081	0.579499	0.045	0.037	1.50E-09
rs9528775	13	65078624	T	C	21.138	0.166	0.040	0.4937	0.027	0.036	2.66E-06
rs9553523	13	25630049	A	G	23.363	-0.222	0.055	0.8699	0.009	0.046	6.21E-07
rs9858213	3	49731861	T	G	83.737	0.284	0.047	0.1846	-0.062	0.031	2.43E-20
**CHC**											
rs11776106	8	49260427	T	C	21.483	1.034	0.223	3.59E-06	-0.014	0.113	0.8991
rs12613265	2	25329898	C	T	24.068	-0.083	0.017	9.67E-07	0.105	0.046	0.02231
rs12927082	16	70810962	T	A	21.841	1.222	0.262	2.97E-06	0.110	0.123	0.3693
rs143003569	17	76540839	T	C	21.991	-0.103	0.022	2.67E-06	-0.080	0.065	0.2172
rs148302083	4	134395270	T	C	22.755	1.247	0.262	1.84E-06	0.167	0.131	0.2039
rs149513365	2	231646702	T	C	23.766	3.621	0.743	1.09E-06	0.397	0.368	0.2808
rs189938683	20	18105918	A	G	21.310	1.979	0.429	3.91E-06	-0.209	0.144	0.1466
rs2052213	7	93865697	G	T	29.305	1.222	0.226	6.22E-08	0.107	0.109	0.3265
rs2212335	11	88748687	A	T	22.670	-0.116	0.024	1.90E-06	-0.029	0.073	0.6888
rs29232	6	29611431	T	C	28.273	0.099	0.019	1.02E-07	-0.003	0.041	0.9381

rs3130506	6	31145451	A	G	23.733	0.110	0.023	1.08E-06	0.086	0.045	0.0546701
rs35800511	6	32636433	G	A	30.196	0.111	0.020	3.88E-08	0.090	0.073	0.2176
rs4778084	15	93603862	C	T	22.081	-0.102	0.022	2.70E-06	-0.015	0.041	0.717
rs550678256	14	45385952	T	A	21.017	1.333	0.291	4.55E-06	0.329	0.185	0.0753494
rs62283057	4	6276805	T	C	21.407	0.568	0.123	3.72E-06	0.034	0.058	0.5581
rs62445574	7	14252711	G	A	21.443	0.416	0.090	3.67E-06	0.063	0.073	0.3925
rs75918461	17	35111303	T	C	23.031	-0.108	0.022	1.65E-06	0.113	0.045	0.0120901
rs8113007	19	39743103	T	A	92.941	0.271	0.028	5.42E-22	0.048	0.047	0.3073
rs9275267	6	32662425	G	A	49.080	-0.123	0.018	2.32E-12	0.060	0.048	0.2081
rs9501400	6	32394184	A	G	42.212	0.111	0.017	8.05E-11	-0.048	0.050	0.3391

SNP: single nucleotide polymorphism; AIH: Autoimmune Hepatitis; PBC: Primary Biliary Cholangitis; PSC: Primary Sclerosing Cholangitis; CHC: chronic hepatitis C; HT: Hashimoto's thyroiditis; SE: standard error;

Table S2. Pleiotropy and heterogeneity test between AILD and TD

Exposure	Outcome	Heterogeneity			Pleiotropy			Model*
		IVW			MR-Egger			
		Q	*df*	*P*	*Intercept*	*Se*	*P*	
AIH	Hypothyroidism	10.585	10	0.391	0.000	0.000	0.838	1
	Hyperthyroidism	2.087	6	0.912	0.000	0.000	0.387	1
	HT	79.503	12	<0.001	-0.027	0.044	0.552	2
	Thyroid cancer	14.472	12	0.272	-0.026	0.042	0.546	1
PBC	Hypothyroidism	1387.100	94	<0.001	0.001	0.000	**0.122**	2
	Hyperthyroidism	376.275	83	<0.001	0.000	0.000	0.051	2
	HT	307.253	100	<0.001	0.023	0.008	**0.004**	2
	Thyroid cancer	119.791	100	0.086	-0.005	0.011	0.672	1
PSC	Hypothyroidism	1675.533	45	<0.001	0.002	0.001	0.005	2
	Hyperthyroidism	305.700	33	<0.001	0.000	0.000	**0.807**	2
	HT	327.638	49	<0.001	0.033	0.013	0.016	2
	Thyroid cancer	55.345	49	0.248	-0.006	0.013	0.629	1
CHC	Hypothyroidism	322.246	16	<0.001	-0.002	0.001	**0.217**	2
	Hyperthyroidism	176.295	8	<0.001	0.000	0.001	**0.965**	2
	HT	61.112	19	<0.001	-0.021	0.013	**0.141**	2
	Thyroid cancer	30.855	19	0.042	-0.014	0.021	**0.516**	2
Hypothyroidism	AIH	394.065	311	0.001	0.004	0.009	**0.613**	2
Hyperthyroidism	AIH	91.331	44	<0.001	-0.067	0.028	**0.024**	2
HT	AIH	87.316	60	0.012	-0.044	0.020	**0.029**	2
Thyroid cancer	AIH	48.068	49	0.511	-0.005	0.007	**0.518**	1

Hypothyroidism	PBC	854.026	82	<0.001	0.026	0.020	**0.185**	2
Hyperthyroidism		259.143	25	<0.001	-0.015	0.031	0.621	2
HT		244.998	33	<0.001	0.021	0.025	0.410	2
Thyroid cancer		3.841	6	0.698	0.025	0.027	0.403	1
Hypothyroidism	PSC	892.241	224	<0.001	-0.006	0.012	**0.622**	2
Hyperthyroidism		269.278	16	<0.001	-0.164	0.056	**0.090**	2
HT		251.173	41	<0.001	-0.015	0.037	**0.679**	2
Thyroid cancer		11.321	9	0.254	0.037	0.035	0.318	1

*1, fixed mode effects; 2, multiplicative random effects; PSC, primary sclerosing cholangitis; IVW, inverse-variance weighted; AIH: Autoimmune Hepatitis; PBC: Primary Biliary Cholangitis; PSC: Primary Sclerosing Cholangitis; CHC: chronic hepatitis C; HT: Hashimoto's thyroiditis

Table S3. Effect estimates of the associations between genetically predicted exposures and risk ofAILD

SNP	Chr	Position	Effect Allele	Other Allele	F	Association with exposure			Association with AIH		
						Beta	SE	*P*	Beta	SE	*P*
**Hypothyroidism**											
rs10021756	4	40285931	T	C	23.600	0.006	0.001	1.20E-06	0.406	0.183	0.026
rs10032998	4	80957826	A	G	22.219	-0.002	0.000	2.40E-06	-0.018	0.048	0.710
rs10036386	5	76543603	T	C	50.908	0.003	0.000	9.70E-13	0.040	0.049	0.413
rs10077159	5	142822029	A	T	29.564	-0.003	0.000	5.40E-08	0.062	0.053	0.240
rs10087240	8	129012574	T	C	27.847	0.002	0.000	1.30E-07	0.065	0.048	0.172
rs10147094	14	99727591	G	A	21.814	0.002	0.000	3.00E-06	0.071	0.049	0.149
rs10277273	7	4785129	G	T	35.139	-0.003	0.000	3.10E-09	-0.035	0.053	0.507
rs1032129	8	119951900	C	A	39.530	-0.003	0.000	3.20E-10	-0.056	0.049	0.251
rs10424978	19	4837557	A	C	95.380	-0.004	0.000	1.60E-22	0.111	0.049	0.023
rs10440761	5	108784915	G	A	22.365	0.008	0.002	2.30E-06	0.157	0.182	0.390
rs1050976	6	408079	T	C	53.308	0.003	0.000	2.90E-13	-0.040	0.048	0.404
rs10737048	10	6197459	G	A	23.130	0.006	0.001	1.50E-06	0.128	0.095	0.176
rs10742340	11	35317712	C	T	79.585	0.004	0.000	4.60E-19	0.102	0.049	0.036
rs10748781	10	101283330	A	C	51.113	-0.003	0.000	8.70E-13	0.016	0.050	0.756
rs10761620	10	64057202	G	A	58.590	-0.003	0.000	1.90E-14	-0.066	0.050	0.189
rs10772561	12	12572548	C	T	27.866	0.002	0.000	1.30E-07	0.108	0.051	0.033
rs10774664	12	113263878	C	T	28.779	0.003	0.001	8.10E-08	-0.084	0.079	0.290
rs1079418	6	166047034	G	A	37.013	-0.003	0.000	1.20E-09	-0.038	0.054	0.473
rs10818007	9	100498968	C	G	23.466	0.004	0.001	1.30E-06	-0.092	0.114	0.420
rs10859679	12	94556678	C	A	31.170	0.003	0.001	2.40E-08	-0.055	0.068	0.420
rs1088897	17	8876494	G	A	27.840	0.003	0.001	1.30E-07	0.027	0.053	0.604
rs10917470	1	19845206	A	G	38.192	0.003	0.000	6.40E-10	-0.015	0.048	0.749
rs10930013	2	162070325	A	G	35.637	0.003	0.000	2.40E-09	0.002	0.048	0.962
rs10937477	3	191048462	T	C	27.994	0.003	0.001	1.20E-07	0.132	0.055	0.016
rs10940534	5	56583527	G	T	23.110	-0.002	0.000	1.50E-06	-0.097	0.054	0.071
rs10956412	8	129162497	C	A	50.706	-0.004	0.001	1.10E-12	-0.041	0.072	0.564
rs10974452	9	4315250	C	G	22.213	0.004	0.001	2.40E-06	-0.057	0.111	0.607

rs11064860	12	110489208	T	C	27.990	-0.004	0.001	1.20E-07	0.009	0.075	0.901
rs11073337	15	38847763	C	A	69.817	0.004	0.001	6.50E-17	-0.019	0.053	0.716
rs11177053	12	68499237	C	T	31.438	-0.003	0.000	2.10E-08	0.104	0.050	0.038
rs111915798	8	129210479	A	G	26.263	0.007	0.001	3.00E-07	0.267	0.147	0.070
rs112165453	2	204664815	T	C	31.053	0.009	0.002	2.50E-08	0.288	0.234	0.220
rs1123751	19	1183851	G	C	25.748	-0.003	0.001	3.90E-07	-0.043	0.058	0.460
rs11256448	10	6079479	G	A	44.083	0.003	0.001	3.10E-11	0.002	0.053	0.978
rs11258303	10	6405534	A	C	52.555	0.004	0.001	4.20E-13	-0.085	0.051	0.099
rs11265410	1	160430580	C	T	26.230	-0.004	0.001	3.00E-07	-0.097	0.077	0.208
rs112799045	7	2939352	A	G	28.618	0.005	0.001	8.80E-08	-0.117	0.165	0.479
rs113473633	4	103449131	G	A	46.253	-0.010	0.001	1.00E-11	-0.077	0.196	0.695
rs114077552	6	30254253	C	A	49.161	-0.012	0.002	2.40E-12	-0.155	0.167	0.353
rs114378220	5	110566360	T	C	43.294	0.006	0.001	4.70E-11	0.011	0.105	0.917
rs114611882	6	30320209	A	G	23.341	-0.006	0.001	1.40E-06	-0.220	0.129	0.088
rs114840990	6	33132935	A	T	21.135	-0.006	0.001	4.30E-06	0.163	0.175	0.354
rs1150258	1	207074905	C	T	23.163	0.002	0.000	1.50E-06	-0.038	0.048	0.431
rs11582506	1	117277321	G	A	25.037	0.002	0.000	5.60E-07	0.007	0.049	0.881
rs116229144	6	31236800	A	G	78.313	0.015	0.002	8.80E-19	-0.076	0.433	0.861
rs11675342	2	1407628	T	C	126.238	0.005	0.000	2.70E-29	0.013	0.048	0.791
rs11782370	8	23370018	T	C	29.946	-0.003	0.001	4.40E-08	0.026	0.060	0.661
rs11783023	8	141639262	T	C	40.650	-0.003	0.000	1.80E-10	0.000	0.050	0.994
rs11822813	11	93912638	G	A	35.598	-0.005	0.001	2.40E-09	-0.122	0.081	0.133
rs11901769	2	160346257	T	A	31.899	0.003	0.001	1.60E-08	-0.047	0.087	0.593
rs11902277	2	204924525	A	G	21.963	-0.005	0.001	2.80E-06	0.119	0.101	0.239
rs11926659	3	105499839	G	A	27.222	-0.003	0.001	1.80E-07	-0.079	0.067	0.238
rs11939242	4	20940352	C	A	22.802	-0.004	0.001	1.80E-06	0.069	0.091	0.452
rs12117927	1	236629134	A	C	38.099	0.003	0.000	6.70E-10	0.010	0.052	0.847
rs1217238	1	114131553	A	G	41.697	-0.003	0.000	1.10E-10	-0.100	0.053	0.058
rs12191243	6	135446826	G	C	30.740	0.003	0.001	2.90E-08	-0.044	0.054	0.420
rs12271161	11	116979911	A	G	40.733	-0.004	0.001	1.70E-10	-0.032	0.055	0.563
rs12349571	9	127093743	G	A	31.999	-0.003	0.000	1.50E-08	-0.027	0.050	0.590
rs12403944	1	9699560	A	T	25.839	0.003	0.001	3.70E-07	0.043	0.057	0.452
rs12482947	21	43852037	C	T	33.990	0.003	0.000	5.50E-09	0.018	0.048	0.715

rs12485900	3	192623779	T	C	29.164	0.002	0.000	6.60E-08	-0.050	0.050	0.318
rs1257926	14	98692996	A	G	38.776	0.003	0.000	4.80E-10	-0.033	0.048	0.489
rs12582330	12	103892941	T	G	63.481	-0.004	0.000	1.60E-15	-0.118	0.051	0.022
rs12630450	3	169480204	G	A	22.995	0.002	0.001	1.60E-06	0.136	0.053	0.010
rs12634152	3	188121019	T	C	213.512	-0.007	0.000	2.40E-48	-0.032	0.048	0.508
rs12683611	9	101812933	A	G	21.348	0.002	0.001	3.80E-06	0.151	0.062	0.014
rs12697352	5	35837234	A	G	30.977	-0.003	0.000	2.60E-08	-0.102	0.050	0.039
rs12720299	19	10468668	G	C	24.661	0.003	0.001	6.80E-07	0.097	0.070	0.168
rs12722022	6	33048564	A	G	43.184	-0.008	0.001	5.00E-11	-0.007	0.200	0.973
rs12902447	15	86027453	G	A	23.166	-0.002	0.000	1.50E-06	-0.026	0.054	0.627
rs12920568	16	9227943	C	A	25.900	0.003	0.001	3.60E-07	0.114	0.061	0.060
rs12981033	19	50197406	G	A	43.720	-0.003	0.000	3.80E-11	0.053	0.050	0.289
rs13076468	3	5022961	C	A	33.923	0.003	0.001	5.70E-09	-0.048	0.052	0.362
rs13090803	3	105934953	T	G	78.620	0.005	0.001	7.50E-19	0.076	0.064	0.240
rs13107325	4	103188709	T	C	22.463	-0.004	0.001	2.10E-06	-0.026	0.138	0.848
rs1317840	6	43806921	C	A	27.167	0.002	0.000	1.90E-07	-0.011	0.048	0.818
rs13299616	9	123594803	C	T	23.530	-0.002	0.000	1.20E-06	-0.022	0.048	0.646
rs13360007	5	156577720	G	A	30.918	0.004	0.001	2.70E-08	-0.107	0.074	0.149
rs1343986	12	103772733	C	G	23.562	-0.004	0.001	1.20E-06	-0.021	0.087	0.806
rs138028125	13	28712689	G	C	21.061	0.006	0.001	4.40E-06	-0.186	0.180	0.302
rs1381286	14	98597582	G	C	24.427	0.003	0.001	7.70E-07	0.075	0.063	0.235
rs138453996	16	67349478	A	G	43.944	0.010	0.002	3.40E-11	-0.064	0.180	0.721
rs138818878	13	43148546	G	C	31.383	0.007	0.001	2.10E-08	0.208	0.134	0.119
rs143814920	19	8625196	G	A	21.428	0.003	0.001	3.70E-06	0.038	0.081	0.643
rs145269503	6	31705126	A	G	29.801	-0.008	0.002	4.80E-08	-0.172	0.147	0.242
rs147423075	17	29284707	T	C	24.140	0.006	0.001	9.00E-07	0.093	0.145	0.521
rs1474466	6	112098264	G	A	25.692	-0.002	0.000	4.00E-07	-0.034	0.048	0.481
rs147910466	2	163522976	T	A	36.421	-0.012	0.002	1.60E-09	-0.157	0.224	0.484
rs1479559	5	76478418	T	C	21.454	-0.003	0.001	3.60E-06	0.076	0.063	0.228
rs148709337	19	21632109	G	C	21.720	0.008	0.002	3.20E-06	0.060	0.251	0.810
rs149492690	2	68553043	T	A	21.401	0.008	0.002	3.70E-06	0.157	0.293	0.591
rs1534430	2	12644736	T	C	71.938	-0.004	0.000	2.20E-17	-0.001	0.048	0.979
rs1543603	6	25413922	G	A	21.901	-0.003	0.001	2.90E-06	-0.090	0.060	0.137

rs1549142	19	18383794	T	C	53.886	0.004	0.001	2.10E-13	0.031	0.055	0.581
rs1561924	8	129569371	A	G	50.125	-0.005	0.001	1.40E-12	-0.159	0.076	0.036
rs1599795	3	119243855	A	T	48.805	0.004	0.001	2.80E-12	-0.050	0.056	0.372
rs16851789	3	105650671	C	G	23.552	0.004	0.001	1.20E-06	-0.022	0.090	0.804
rs17020139	1	108369483	A	G	227.787	0.012	0.001	1.80E-51	-0.122	0.073	0.095
rs17129794	1	67794918	C	A	32.791	0.003	0.001	1.00E-08	0.047	0.078	0.549
rs1723022	1	167405418	T	G	34.863	0.003	0.000	3.50E-09	0.021	0.056	0.706
rs1724088	6	36978745	A	G	31.690	0.003	0.000	1.80E-08	0.081	0.053	0.122
rs17300357	10	6496815	A	G	27.582	0.006	0.001	1.50E-07	-0.061	0.104	0.557
rs17306827	2	174763936	A	G	24.122	0.002	0.000	9.00E-07	-0.035	0.048	0.463
rs174599	11	61621556	C	G	43.621	-0.003	0.000	4.00E-11	0.042	0.048	0.389
rs1782648	10	81060829	A	G	29.669	0.003	0.000	5.10E-08	0.096	0.048	0.047
rs1800520	21	45709906	G	C	22.387	-0.004	0.001	2.20E-06	-0.055	0.074	0.454
rs1810396	8	133918769	G	A	65.908	-0.004	0.000	4.70E-16	0.069	0.055	0.215
rs1811711	2	228670476	G	C	27.503	0.003	0.001	1.60E-07	0.038	0.067	0.572
rs183879	6	25987441	C	G	34.856	0.003	0.001	3.50E-09	0.109	0.089	0.221
rs1872691	16	50350210	A	G	40.379	-0.004	0.001	2.10E-10	-0.029	0.061	0.640
rs1884352	1	8537289	A	G	26.754	-0.002	0.000	2.30E-07	-0.032	0.051	0.537
rs190912457	19	20406686	C	A	21.409	-0.008	0.002	3.70E-06	0.166	0.205	0.420
rs1926193	10	90760606	C	T	23.003	-0.002	0.000	1.60E-06	-0.068	0.048	0.159
rs1985378	22	39678312	G	A	29.311	0.002	0.000	6.20E-08	0.011	0.050	0.834
rs2029751	1	200698286	C	A	41.526	0.004	0.001	1.20E-10	0.068	0.131	0.606
rs2053979	11	47439444	G	A	27.214	0.002	0.000	1.80E-07	0.005	0.055	0.922
rs2111485	2	163110536	G	A	69.140	0.004	0.000	9.20E-17	0.102	0.049	0.037
rs212411	6	159468565	A	G	27.966	0.002	0.000	1.20E-07	0.023	0.048	0.636
rs2130357	6	27886830	T	C	24.328	-0.003	0.001	8.10E-07	-0.028	0.056	0.621
rs2160316	2	202885167	C	T	25.055	0.002	0.000	5.60E-07	-0.010	0.048	0.835
rs221781	7	100295908	G	A	39.915	0.004	0.001	2.70E-10	0.037	0.071	0.603
rs2218245	13	70596378	A	C	21.298	-0.003	0.001	3.90E-06	-0.031	0.066	0.638
rs2234167	1	2494330	A	G	42.839	0.004	0.001	5.90E-11	0.068	0.074	0.355
rs2248605	13	24831467	G	A	29.738	-0.003	0.000	4.90E-08	0.033	0.052	0.533
rs2271973	3	100949668	A	C	22.617	0.003	0.001	2.00E-06	-0.020	0.060	0.739
rs2279396	17	80536908	C	A	22.235	-0.002	0.000	2.40E-06	-0.009	0.049	0.850

rs2280446	12	53451952	T	C	21.479	-0.003	0.001	3.60E-06	-0.026	0.060	0.659
rs2284169	6	30172385	C	A	82.812	-0.006	0.001	9.00E-20	-0.080	0.077	0.297
rs229540	22	37591290	G	T	127.764	0.005	0.000	1.30E-29	-0.044	0.048	0.368
rs2333567	4	177730455	C	T	25.469	0.004	0.001	4.50E-07	-0.078	0.082	0.341
rs2392239	1	101735917	T	C	21.879	-0.002	0.000	2.90E-06	-0.033	0.049	0.502
rs2412975	22	30540590	C	T	42.952	-0.003	0.000	5.60E-11	-0.034	0.048	0.477
rs2415317	14	36609678	A	G	26.612	-0.002	0.000	2.50E-07	-0.035	0.049	0.477
rs244672	5	133419283	T	C	53.299	-0.005	0.001	2.90E-13	0.049	0.059	0.407
rs2473808	1	19638883	C	T	43.413	-0.003	0.000	4.40E-11	0.013	0.049	0.792
rs2476601	1	114377568	G	A	770.620	-0.020	0.001	1.30E-169	-0.121	0.073	0.099
rs2481974	1	28517379	A	C	21.828	-0.002	0.000	3.00E-06	0.047	0.051	0.360
rs2607013	6	31820400	T	C	158.681	0.007	0.001	2.20E-36	-0.056	0.067	0.397
rs2687938	13	50754246	T	C	23.698	0.002	0.000	1.10E-06	0.096	0.048	0.045
rs2744944	6	34658080	C	A	29.168	-0.002	0.000	6.60E-08	-0.107	0.050	0.032
rs28157	5	102595837	T	G	47.803	-0.003	0.000	4.70E-12	-0.088	0.051	0.085
rs2823272	21	16798586	A	T	48.105	-0.003	0.000	4.00E-12	0.005	0.053	0.922
rs28375404	6	32614917	T	C	94.830	-0.011	0.001	2.10E-22	-0.417	0.179	0.020
rs28450181	4	87819369	G	A	34.868	0.003	0.001	3.50E-09	0.047	0.066	0.476
rs2859072	6	32703313	A	G	71.448	-0.004	0.001	2.80E-17	-0.038	0.054	0.479
rs28594633	6	32525486	A	G	409.455	0.011	0.001	4.80E-91	-0.821	0.465	0.078
rs3006986	1	114562181	G	C	22.986	0.003	0.001	1.60E-06	-0.034	0.078	0.660
rs3087243	2	204738919	A	G	370.140	-0.009	0.000	1.70E-82	-0.035	0.050	0.480
rs3093665	6	31545391	C	A	37.391	0.009	0.002	9.70E-10	-0.179	0.265	0.498
rs3184504	12	111884608	C	T	525.303	-0.010	0.000	3.00E-116	-0.109	0.050	0.030
rs337637	4	38604470	A	G	27.461	-0.002	0.000	1.60E-07	0.064	0.049	0.192
rs34221525	5	68798118	A	G	23.620	0.004	0.001	1.20E-06	-0.098	0.139	0.479
rs34477738	9	5447227	G	A	47.999	0.004	0.001	4.30E-12	0.020	0.064	0.757
rs34509786	3	12305916	G	T	56.354	0.004	0.001	6.10E-14	0.020	0.092	0.829
rs34536443	19	10463118	C	G	50.958	-0.008	0.001	9.40E-13	-0.192	0.137	0.160
rs34678053	14	106146147	A	G	57.118	-0.004	0.001	4.10E-14	0.038	0.066	0.559
rs347153	3	32472324	C	A	21.504	-0.002	0.000	3.50E-06	-0.048	0.050	0.338
rs34916533	13	33074674	A	C	22.568	-0.002	0.000	2.00E-06	-0.035	0.082	0.672
rs35021449	6	32530583	T	C	278.090	0.014	0.001	2.00E-62	0.393	0.157	0.012

rs35074907	19	10600418	A	G	32.813	0.009	0.002	1.00E-08	-0.094	0.236	0.691
rs35601864	11	117539660	C	T	21.542	-0.004	0.001	3.50E-06	0.082	0.081	0.317
rs35677470	3	58183636	A	G	28.570	0.004	0.001	9.00E-08	0.070	0.099	0.476
rs35703946	16	86021505	A	G	26.477	-0.003	0.001	2.70E-07	-0.023	0.067	0.735
rs367023	2	8450123	G	A	39.218	-0.003	0.000	3.80E-10	-0.003	0.050	0.952
rs3735477	7	44714512	C	T	25.313	0.002	0.000	4.90E-07	-0.014	0.049	0.782
rs3775291	4	187004074	T	C	69.132	-0.004	0.000	9.20E-17	-0.121	0.051	0.018
rs3784099	14	68749927	A	G	42.864	-0.003	0.000	5.90E-11	-0.003	0.053	0.960
rs3807307	7	128579202	C	T	35.176	0.003	0.000	3.00E-09	0.159	0.048	0.001
rs3850765	10	124139910	C	T	48.478	0.003	0.000	3.30E-12	-0.027	0.050	0.587
rs3862663	11	60727478	A	G	23.814	0.006	0.001	1.10E-06	0.282	0.164	0.085
rs41287542	6	109295120	G	A	25.453	0.005	0.001	4.50E-07	-0.045	0.104	0.664
rs4320727	1	25351581	A	G	39.343	0.003	0.000	3.60E-10	0.048	0.049	0.332
rs4409785	11	95311422	C	T	134.472	0.007	0.001	4.30E-31	-0.005	0.065	0.939
rs4444866	4	40307533	T	C	44.233	-0.003	0.000	2.90E-11	-0.016	0.055	0.772
rs4660154	1	235356301	G	A	21.496	-0.002	0.000	3.50E-06	0.023	0.048	0.635
rs4748400	10	17734360	G	C	26.642	-0.004	0.001	2.40E-07	0.030	0.079	0.703
rs4794063	17	45804494	T	C	61.770	0.004	0.001	3.90E-15	0.056	0.054	0.304
rs479777	11	64107477	C	T	40.934	-0.003	0.000	1.60E-10	-0.063	0.050	0.202
rs4804433	19	7240776	T	G	35.900	-0.003	0.001	2.10E-09	-0.049	0.054	0.367
rs4820437	22	41761290	C	T	29.798	-0.003	0.001	4.80E-08	-0.029	0.054	0.598
rs4824117	22	50895133	G	A	33.801	-0.003	0.000	6.10E-09	-0.001	0.051	0.982
rs4835536	4	149662857	T	G	139.035	-0.006	0.001	4.30E-32	0.020	0.068	0.774
rs484959	1	110366083	C	T	39.445	0.003	0.000	3.40E-10	-0.023	0.049	0.639
rs4885151	13	74697856	G	T	24.561	0.002	0.000	7.20E-07	0.181	0.055	0.001
rs4922066	8	19456270	T	C	21.092	-0.002	0.000	4.40E-06	0.021	0.054	0.691
rs55792153	5	138854203	C	A	24.675	0.003	0.001	6.80E-07	0.134	0.098	0.173
rs55926131	12	115085267	T	C	24.813	0.002	0.000	6.30E-07	0.032	0.048	0.502
rs56249713	18	67533332	C	T	42.208	-0.003	0.000	8.20E-11	-0.076	0.049	0.120
rs56400413	7	77340821	A	T	31.188	0.003	0.000	2.30E-08	-0.013	0.054	0.809
rs573741	1	65455477	A	C	25.547	0.004	0.001	4.30E-07	0.134	0.080	0.092
rs5746009	1	12249568	C	A	29.179	-0.004	0.001	6.60E-08	-0.040	0.083	0.629
rs57465888	6	109593038	G	A	20.964	-0.005	0.001	4.70E-06	0.150	0.137	0.274

rs57871924	3	46282278	A	T	24.021	0.004	0.001	9.50E-07	0.011	0.080	0.894
rs57938373	3	39336038	T	C	43.605	0.004	0.001	4.00E-11	0.082	0.066	0.212
rs58620180	20	62209313	A	G	23.027	0.008	0.002	1.60E-06	-0.017	0.115	0.881
rs5865	2	98373006	T	C	43.255	-0.003	0.000	4.80E-11	-0.073	0.051	0.154
rs59036109	13	97990910	A	C	21.500	-0.002	0.000	3.50E-06	0.058	0.054	0.285
rs59100653	1	21984969	A	G	21.776	0.004	0.001	3.10E-06	0.053	0.083	0.521
rs59183580	11	95425470	G	A	27.428	0.003	0.001	1.60E-07	0.119	0.076	0.119
rs60160146	9	110637549	T	C	29.208	-0.003	0.001	6.50E-08	-0.136	0.072	0.058
rs60600003	7	37382465	G	T	35.984	0.004	0.001	2.00E-09	0.128	0.083	0.122
rs6071959	20	38794945	A	T	23.938	-0.002	0.000	9.90E-07	-0.011	0.055	0.839
rs6111715	20	17860022	C	G	43.248	-0.004	0.001	4.80E-11	-0.008	0.067	0.900
rs6125457	20	47378799	A	G	24.055	0.002	0.000	9.40E-07	0.003	0.050	0.955
rs61759532	17	7240391	T	C	57.922	0.004	0.001	2.70E-14	0.180	0.061	0.003
rs61776678	1	38377021	A	G	40.909	-0.003	0.000	1.60E-10	-0.004	0.048	0.938
rs61778693	1	38651781	T	G	34.526	-0.003	0.001	4.20E-09	0.002	0.057	0.977
rs61907718	11	128172470	A	G	26.056	0.003	0.001	3.30E-07	0.034	0.056	0.543
rs61971960	13	114862857	A	G	21.235	0.004	0.001	4.10E-06	-0.022	0.110	0.844
rs62052470	16	85904222	A	G	21.705	-0.004	0.001	3.20E-06	-0.079	0.105	0.454
rs62182865	2	191434180	C	T	26.731	0.003	0.001	2.30E-07	0.000	0.070	0.997
rs6441938	3	46045619	C	G	24.255	0.003	0.001	8.40E-07	-0.022	0.059	0.707
rs6452444	5	71685736	C	T	32.656	0.003	0.001	1.10E-08	0.085	0.059	0.149
rs6478264	9	100323465	G	A	26.154	0.005	0.001	3.20E-07	-0.170	0.130	0.190
rs6505765	18	12782849	G	C	51.283	0.003	0.000	8.00E-13	0.064	0.050	0.197
rs6517444	21	39344121	G	A	24.345	0.002	0.000	8.10E-07	-0.003	0.050	0.958
rs6602315	10	8547572	C	A	23.205	-0.003	0.001	1.50E-06	0.066	0.075	0.384
rs6602392	10	6078079	A	C	24.392	0.004	0.001	7.90E-07	0.057	0.079	0.471
rs6603785	1	1186502	T	A	32.903	-0.004	0.001	9.70E-09	-0.016	0.066	0.813
rs66749983	13	43063831	T	A	59.823	0.004	0.000	1.00E-14	0.086	0.051	0.094
rs6681271	1	157666644	C	T	26.064	0.002	0.000	3.30E-07	-0.060	0.048	0.207
rs671565	1	24042811	A	G	26.500	-0.002	0.000	2.60E-07	-0.071	0.048	0.144
rs6739788	2	55862355	T	A	32.867	0.006	0.001	9.90E-09	0.007	0.119	0.955
rs678456	1	160464548	C	T	24.180	-0.004	0.001	8.80E-07	0.102	0.082	0.212
rs67927699	2	61187415	C	G	21.219	0.002	0.000	4.10E-06	-0.044	0.051	0.389

rs6798068	3	108162362	A	G	35.034	0.003	0.000	3.20E-09	-0.017	0.055	0.760
rs68189149	17	73820021	T	C	25.882	0.003	0.001	3.60E-07	0.001	0.071	0.984
rs6819295	4	103355489	A	G	21.547	0.002	0.001	3.50E-06	-0.051	0.058	0.381
rs6833591	4	123546282	G	A	37.930	-0.003	0.000	7.30E-10	0.020	0.054	0.715
rs683763	10	89807680	T	G	25.923	0.002	0.000	3.60E-07	0.048	0.049	0.334
rs6915638	6	30722949	T	A	64.353	0.009	0.001	1.00E-15	0.013	0.179	0.941
rs693939	18	23650082	T	C	24.089	-0.002	0.000	9.20E-07	-0.067	0.054	0.216
rs6992869	8	61395832	C	T	40.021	0.003	0.000	2.50E-10	-0.030	0.048	0.530
rs7005834	8	134214204	T	C	45.929	-0.003	0.000	1.20E-11	-0.105	0.054	0.052
rs701292	7	83543341	T	C	22.547	0.002	0.000	2.10E-06	-0.060	0.048	0.209
rs7090530	10	6110875	A	C	84.582	0.004	0.000	3.70E-20	-0.006	0.050	0.908
rs71508903	10	63779871	T	C	137.360	0.007	0.001	1.00E-31	0.034	0.060	0.571
rs71571466	6	167342767	A	G	32.528	0.005	0.001	1.20E-08	0.149	0.107	0.162
rs72807396	10	90605565	A	G	22.643	0.007	0.001	2.00E-06	-0.234	0.154	0.128
rs72850698	2	145417530	C	G	21.839	0.004	0.001	3.00E-06	-0.099	0.094	0.289
rs72934864	2	177639327	C	T	23.889	-0.003	0.001	1.00E-06	0.053	0.061	0.390
rs7297415	12	112661104	A	G	25.908	-0.004	0.001	3.60E-07	-0.134	0.083	0.104
rs73068668	19	55763262	A	G	29.654	0.005	0.001	5.20E-08	-0.004	0.092	0.967
rs731151	17	40279903	A	G	49.110	0.004	0.001	2.40E-12	0.067	0.060	0.265
rs73119796	12	65090056	A	G	23.787	0.005	0.001	1.10E-06	-0.010	0.091	0.915
rs7314285	12	111522026	G	T	23.337	-0.004	0.001	1.40E-06	0.112	0.101	0.266
rs739091	22	40317126	A	C	23.810	-0.002	0.000	1.10E-06	-0.074	0.049	0.125
rs7417283	1	108303686	T	C	40.199	0.004	0.001	2.30E-10	-0.124	0.060	0.040
rs7441808	4	26090375	G	A	65.867	0.004	0.000	4.80E-16	0.033	0.055	0.551
rs74435468	1	65455166	C	G	27.643	0.005	0.001	1.50E-07	0.016	0.089	0.859
rs75459234	6	89947312	A	C	21.127	0.007	0.002	4.30E-06	-0.277	0.237	0.242
rs7575113	2	181885295	C	G	26.073	0.002	0.000	3.30E-07	-0.028	0.053	0.593
rs7581413	2	145058878	A	T	23.029	-0.002	0.000	1.60E-06	-0.022	0.049	0.656
rs7582694	2	191970120	G	C	163.912	-0.007	0.001	1.60E-37	-0.141	0.056	0.012
rs7583027	2	62544391	C	A	39.473	0.003	0.000	3.30E-10	-0.014	0.051	0.780
rs75891606	6	502106	G	A	21.700	0.005	0.001	3.20E-06	0.562	0.168	0.001
rs7596240	2	242444173	G	A	33.794	0.003	0.000	6.10E-09	0.032	0.056	0.572
rs761357	6	135902599	T	A	39.412	0.003	0.000	3.40E-10	-0.018	0.050	0.717

rs7620353	3	150947555	T	C	23.915	-0.003	0.001	1.00E-06	-0.006	0.065	0.928
rs76280316	13	28640486	T	C	20.899	-0.005	0.001	4.80E-06	0.044	0.106	0.679
rs7649344	3	37006396	C	T	34.603	-0.003	0.000	4.00E-09	-0.167	0.050	0.001
rs76930710	12	68434459	C	T	30.056	-0.006	0.001	4.20E-08	-0.184	0.145	0.205
rs76946320	2	12485667	C	T	22.116	0.006	0.001	2.60E-06	0.189	0.156	0.225
rs7705526	5	1285974	A	C	36.678	-0.003	0.000	1.40E-09	0.055	0.051	0.280
rs7710070	5	10596269	T	G	20.994	-0.002	0.000	4.60E-06	-0.042	0.049	0.387
rs772920	12	56390364	G	C	58.601	0.004	0.000	1.90E-14	-0.122	0.052	0.018
rs77357117	2	162757307	A	G	24.000	0.005	0.001	9.60E-07	-0.019	0.099	0.844
rs7746336	6	31270987	T	C	26.823	0.004	0.001	2.20E-07	-0.088	0.104	0.398
rs7762909	6	31321578	G	A	159.729	0.006	0.000	1.30E-36	0.115	0.050	0.023
rs78041260	3	188008959	A	G	28.220	0.004	0.001	1.10E-07	-0.088	0.083	0.285
rs7822344	8	117418375	T	C	21.115	0.003	0.001	4.30E-06	0.012	0.057	0.833
rs78328982	3	121734641	A	G	44.269	0.006	0.001	2.90E-11	-0.029	0.120	0.807
rs7849424	9	100876668	C	T	29.865	0.002	0.000	4.60E-08	0.025	0.049	0.608
rs7850258	9	100549013	G	A	443.462	0.010	0.000	1.90E-98	0.022	0.052	0.668
rs79192893	8	29852754	T	C	21.373	-0.005	0.001	3.80E-06	-0.033	0.129	0.800
rs79281532	6	239806	G	A	22.494	-0.005	0.001	2.10E-06	-0.140	0.104	0.178
rs7936397	11	577534	A	G	34.844	-0.003	0.000	3.60E-09	-0.010	0.059	0.869
rs7937786	11	332857	A	C	29.168	-0.002	0.000	6.60E-08	-0.055	0.052	0.292
rs79490353	13	28623048	C	T	53.770	0.010	0.001	2.30E-13	0.098	0.186	0.597
rs7955910	12	122654382	T	G	22.335	0.002	0.000	2.30E-06	-0.025	0.049	0.609
rs79708723	6	148533113	A	G	34.184	0.007	0.001	5.00E-09	-0.063	0.168	0.708
rs8047543	16	10956188	G	C	23.358	-0.002	0.001	1.30E-06	-0.071	0.052	0.175
rs8054578	16	79316815	G	A	41.392	-0.003	0.001	1.20E-10	0.044	0.059	0.455
rs8093850	18	77178302	G	A	34.245	0.003	0.000	4.90E-09	-0.041	0.050	0.418
rs838144	19	49250239	T	C	26.960	-0.002	0.000	2.10E-07	-0.058	0.050	0.248
rs8743	16	11852354	C	A	23.772	-0.002	0.000	1.10E-06	0.014	0.049	0.780
rs897586	8	128193294	A	G	56.706	-0.003	0.000	5.10E-14	0.050	0.050	0.319
rs916963	7	26146351	A	G	25.120	-0.003	0.001	5.40E-07	-0.102	0.060	0.089
rs926103	1	156784982	C	T	28.894	-0.003	0.000	7.60E-08	0.014	0.050	0.785
rs9272426	6	32605189	G	A	764.693	0.012	0.000	2.60E-168	0.416	0.077	0.000
rs9277627	6	33081979	C	G	258.406	0.010	0.001	3.80E-58	-0.050	0.063	0.426

rs9288593	2	225756496	G	C	24.710	-0.002	0.000	6.70E-07	0.031	0.053	0.559
rs9291444	4	10713674	T	C	71.146	0.004	0.000	3.30E-17	0.001	0.047	0.985
rs9296009	6	32114515	T	A	92.070	0.005	0.001	8.40E-22	0.228	0.062	0.000
rs9296079	6	33072539	T	G	68.222	-0.011	0.001	1.50E-16	0.095	0.121	0.432
rs9347170	6	167405187	T	C	143.603	-0.006	0.000	4.30E-33	0.041	0.049	0.408
rs9357120	6	31230717	A	T	336.077	-0.009	0.000	4.60E-75	-0.038	0.053	0.481
rs9358913	6	26239404	G	A	25.005	-0.003	0.001	5.70E-07	0.004	0.052	0.944
rs9380522	6	35537964	T	C	43.490	0.003	0.000	4.30E-11	0.118	0.057	0.037
rs9386512	6	106599710	G	A	21.898	0.004	0.001	2.90E-06	0.046	0.081	0.574
rs9497965	6	148521292	T	C	74.586	0.004	0.000	5.80E-18	-0.044	0.049	0.371
rs9507287	13	24786577	C	T	179.303	-0.006	0.000	6.90E-41	0.036	0.051	0.478
rs9521838	13	111206226	A	G	29.471	-0.003	0.001	5.70E-08	-0.056	0.058	0.330
rs952579	6	21884440	A	G	22.481	0.003	0.001	2.10E-06	0.101	0.062	0.107
rs9557168	13	99807350	A	G	30.824	-0.003	0.001	2.80E-08	-0.015	0.104	0.887
rs9619183	22	31688583	T	G	26.406	-0.005	0.001	2.80E-07	-0.013	0.114	0.907
rs969478	21	34766467	T	G	23.026	0.003	0.001	1.60E-06	0.027	0.063	0.662
rs9697210	9	131468740	A	G	34.201	-0.004	0.001	5.00E-09	0.102	0.077	0.185
rs970987	9	21585265	A	C	68.962	-0.004	0.000	1.00E-16	-0.021	0.050	0.681
rs979543	15	84352564	A	C	22.985	0.003	0.001	1.60E-06	0.060	0.059	0.309
rs9831283	3	149008625	A	G	27.629	0.006	0.001	1.50E-07	-0.103	0.095	0.277
rs9921917	16	57374710	T	C	23.519	-0.004	0.001	1.20E-06	0.012	0.090	0.893
**Hyperthyroidism**											
rs10087240	8	129012574	T	C	34.986	0.001	0.000	3.30E-09	0.065	0.048	0.172
rs10195156	2	168445853	C	T	21.290	0.001	0.000	3.90E-06	-0.048	0.059	0.414
rs11008355	10	31412561	C	G	24.904	0.001	0.000	6.00E-07	0.000	0.058	0.998
rs112335954	6	32534115	A	T	119.739	0.003	0.000	7.20E-28	-0.393	0.855	0.646
rs11642757	16	79753467	A	G	27.555	0.001	0.000	1.50E-07	-0.006	0.050	0.898
rs11671004	19	35987994	T	C	27.024	0.001	0.000	2.00E-07	-0.058	0.056	0.294
rs11683617	2	216593693	T	C	21.888	0.001	0.000	2.90E-06	0.047	0.058	0.419
rs11724048	4	7672785	T	G	22.380	0.001	0.000	2.20E-06	0.013	0.059	0.822
rs11729055	4	10709726	T	C	21.395	0.001	0.000	3.70E-06	-0.002	0.050	0.966
rs11736377	4	40301264	T	C	27.837	-0.001	0.000	1.30E-07	-0.055	0.055	0.312
rs11885501	2	10471666	G	A	23.301	0.001	0.000	1.40E-06	0.068	0.050	0.175

rs12741781	1	243428152	G	T	34.502	0.001	0.000	4.30E-09	0.078	0.052	0.133
rs13250295	8	134212652	T	C	31.636	-0.001	0.000	1.90E-08	-0.107	0.054	0.047
rs1559810	3	188124354	A	C	30.758	0.001	0.000	2.90E-08	0.056	0.048	0.243
rs17651741	15	38869666	A	G	21.627	0.001	0.000	3.30E-06	-0.010	0.061	0.868
rs17664732	8	32437118	C	T	23.273	0.001	0.000	1.40E-06	0.050	0.052	0.336
rs1794279	6	32667595	T	G	606.488	0.007	0.000	6.50E-134	0.844	0.139	0.000
rs185320691	6	32490292	C	G	49.949	-0.002	0.000	1.60E-12	0.306	0.153	0.046
rs2074466	6	29408313	A	C	25.108	-0.001	0.000	5.40E-07	-0.156	0.068	0.022
rs2160215	14	81461472	C	T	200.417	0.003	0.000	1.70E-45	0.078	0.051	0.125
rs2394186	6	29816421	G	A	43.947	-0.002	0.000	3.40E-11	-0.078	0.064	0.221
rs2523590	6	31327064	C	T	176.307	-0.003	0.000	3.10E-40	-0.103	0.054	0.058
rs2523998	6	29904865	G	T	21.344	-0.001	0.000	3.80E-06	-0.109	0.098	0.268
rs28752803	6	31292134	T	C	119.857	0.003	0.000	6.80E-28	-0.312	0.163	0.055
rs3087243	2	204738919	A	G	101.401	-0.002	0.000	7.50E-24	-0.035	0.050	0.480
rs3113288	7	68532146	G	A	21.722	0.001	0.000	3.20E-06	-0.050	0.048	0.294
rs3129294	6	33084671	C	A	51.643	-0.001	0.000	6.70E-13	-0.080	0.052	0.129
rs409602	5	156608284	A	T	29.866	0.001	0.000	4.60E-08	-0.088	0.075	0.240
rs4409785	11	95311422	C	T	37.480	0.001	0.000	9.20E-10	-0.005	0.065	0.939
rs444210	6	167390242	G	A	25.076	0.001	0.000	5.50E-07	-0.026	0.047	0.581
rs56079671	7	139466379	A	G	21.445	-0.001	0.000	3.60E-06	0.060	0.066	0.362
rs59129932	19	4838056	G	A	27.861	-0.001	0.000	1.30E-07	0.093	0.049	0.060
rs6427397	1	157705725	T	C	24.154	0.001	0.000	8.90E-07	-0.081	0.048	0.094
rs6432090	2	10445622	G	A	20.972	-0.001	0.000	4.70E-06	0.028	0.051	0.579
rs6679677	1	114303808	A	C	68.239	0.002	0.000	1.40E-16	0.128	0.073	0.081
rs6906566	6	31097820	T	C	85.245	-0.002	0.000	2.60E-20	-0.060	0.069	0.383
rs6911639	6	32978178	C	T	23.572	-0.001	0.000	1.20E-06	0.034	0.060	0.575
rs7090530	10	6110875	A	C	34.383	0.001	0.000	4.50E-09	-0.006	0.050	0.908
rs7127307	11	128187383	C	T	24.980	0.001	0.000	5.80E-07	0.048	0.048	0.318
rs71508903	10	63779871	T	C	26.012	0.001	0.000	3.40E-07	0.034	0.060	0.571
rs71542456	6	32631807	G	A	226.816	0.004	0.000	2.90E-51	-0.481	0.325	0.139
rs72723620	14	75995389	A	G	22.190	0.001	0.000	2.50E-06	0.086	0.083	0.300
rs72928038	6	90976768	A	G	22.390	0.001	0.000	2.20E-06	0.281	0.073	0.000
rs820048	3	27997228	C	A	24.056	0.001	0.000	9.40E-07	0.100	0.088	0.260

rs9894461	17	26485069	T	C	21.119	0.001	0.000	4.30E-06	-0.051	0.063	0.414
**HT**											
rs10424978	19	4837557	A	C	23.998	-0.082	0.017	8.97E-07	0.111	0.049	0.023
rs10483157	22	30345792	G	A	25.615	0.166	0.033	4.14E-07	0.234	0.103	0.022
rs10786679	10	104315667	A	G	22.758	-0.087	0.018	1.87E-06	-0.021	0.053	0.687
rs10880930	12	46574248	T	C	21.110	-0.085	0.019	4.26E-06	0.073	0.053	0.168
rs111358861	4	106712204	G	A	21.632	1.820	0.391	3.30E-06	0.393	0.559	0.482
rs1115587	10	64038901	A	G	27.278	-0.096	0.018	1.82E-07	-0.093	0.053	0.080
rs11211645	2	1412581	G	A	28.530	0.089	0.017	9.33E-08	0.004	0.049	0.937
rs113075322	8	119857113	G	C	24.509	-0.141	0.028	7.18E-07	0.001	0.094	0.991
rs113277625	6	32474340	C	A	28.650	0.390	0.073	8.73E-08	0.519	0.182	0.004
rs11611029	12	56385579	T	C	32.517	-0.096	0.017	1.16E-08	0.081	0.049	0.096
rs117167029	2	215411930	C	G	24.344	2.989	0.606	8.05E-07	0.275	1.185	0.817
rs117369326	22	41865334	A	G	21.454	0.235	0.051	3.55E-06	-0.068	0.138	0.621
rs118075239	13	43458697	C	T	21.564	0.385	0.083	3.45E-06	0.147	0.226	0.514
rs11822254	11	86073315	G	A	21.418	-0.080	0.017	3.52E-06	-0.038	0.050	0.449
rs11889341	2	191943742	T	C	42.489	0.125	0.019	6.64E-11	0.141	0.056	0.012
rs12517193	5	76008682	A	G	25.244	0.082	0.016	4.90E-07	0.080	0.048	0.095
rs1317983	6	43806335	C	T	27.317	0.095	0.018	1.65E-07	-0.045	0.052	0.385
rs13249705	8	141649551	G	A	25.712	-0.093	0.018	3.78E-07	0.021	0.054	0.695
rs140111921	20	31895623	A	G	23.366	-0.163	0.034	1.34E-06	0.021	0.107	0.843
rs146346485	6	32263873	A	G	34.942	-0.253	0.043	3.34E-09	-0.060	0.131	0.648
rs150619443	10	93808024	C	G	25.252	0.440	0.088	5.08E-07	0.286	0.235	0.224
rs1534430	2	12644736	T	C	28.509	-0.088	0.017	9.40E-08	-0.001	0.048	0.979
rs1611499	6	29886646	T	C	22.323	-0.323	0.068	2.27E-06	-0.096	0.110	0.383
rs17490285	14	72279703	G	A	24.028	-0.185	0.038	9.26E-07	-0.082	0.096	0.394
rs17675149	12	63009635	A	G	26.709	0.240	0.046	2.31E-07	0.003	0.139	0.983
rs17687621	9	100721555	T	C	23.163	-0.105	0.022	1.47E-06	0.022	0.067	0.749
rs1887414	6	109292736	C	T	27.142	0.117	0.022	1.97E-07	-0.021	0.067	0.759
rs2072109	6	30070275	G	C	33.205	-0.187	0.032	8.59E-09	-0.013	0.088	0.883
rs2111485	2	163110536	G	A	21.281	0.078	0.017	4.19E-06	0.102	0.049	0.037
rs229533	22	37587111	C	A	23.934	0.082	0.017	9.26E-07	-0.045	0.048	0.351
rs2347446	22	29144893	C	T	22.549	0.083	0.018	2.15E-06	-0.013	0.051	0.803

rs2476601	1	114377568	G	A	199.848	-0.331	0.023	3.49E-45	-0.121	0.073	0.099
rs2802509	13	40966599	C	T	20.875	0.080	0.017	4.67E-06	0.061	0.051	0.228
rs28367132	4	149639865	A	G	28.016	-0.136	0.026	1.21E-07	0.004	0.069	0.954
rs28450758	1	247512163	A	C	21.214	0.141	0.031	4.17E-06	0.155	0.091	0.087
rs3128915	6	29863243	T	C	28.494	0.385	0.072	9.51E-08	0.227	0.179	0.204
rs3131627	6	31415065	G	A	21.833	0.079	0.017	3.14E-06	0.104	0.049	0.032
rs3184504	12	111884608	C	T	109.576	-0.179	0.017	1.40E-25	-0.109	0.050	0.030
rs34536443	19	10463118	C	G	26.562	-0.255	0.049	2.47E-07	-0.192	0.137	0.160
rs34545402	5	41881226	A	C	20.858	0.075	0.016	4.82E-06	0.000	0.048	0.996
rs34636506	2	204692216	G	A	58.488	-0.135	0.018	1.92E-14	-0.027	0.050	0.592
rs41553715	6	31324509	A	T	37.622	1.000	0.163	8.51E-10	0.749	0.365	0.040
rs56175143	1	114599755	A	G	32.375	0.220	0.039	1.33E-08	0.204	0.139	0.141
rs61830417	10	11102913	G	A	21.489	0.274	0.059	3.59E-06	0.115	0.174	0.510
rs62076103	17	45888374	G	A	27.999	-0.185	0.035	1.23E-07	-0.039	0.101	0.698
rs6501550	17	70497869	T	C	23.292	-0.181	0.037	1.42E-06	0.047	0.113	0.678
rs73165154	7	155360251	G	A	21.422	-0.153	0.033	3.76E-06	-0.026	0.093	0.777
rs73192661	3	188128794	T	C	36.574	-0.102	0.017	1.45E-09	-0.042	0.048	0.390
rs7335748	13	89319468	G	A	22.228	0.377	0.080	2.41E-06	0.372	0.209	0.075
rs757024	22	30471679	C	G	32.732	-0.094	0.017	1.08E-08	-0.026	0.048	0.585
rs77331047	2	220135635	T	A	23.665	-0.176	0.036	1.11E-06	-0.171	0.108	0.112
rs7861040	9	127044135	C	G	24.073	0.084	0.017	9.08E-07	0.022	0.050	0.655
rs79219789	11	69065730	C	G	21.948	-0.155	0.033	2.84E-06	-0.119	0.103	0.246
rs80173139	1	108365138	G	A	75.797	0.213	0.025	3.01E-18	-0.124	0.073	0.089
rs8084503	18	71061287	T	C	27.079	0.138	0.027	1.97E-07	-0.022	0.076	0.776
rs847803	2	64982415	G	A	24.459	0.091	0.018	7.91E-07	-0.011	0.053	0.839
rs9264794	6	31270304	G	A	23.337	0.163	0.034	1.38E-06	-0.073	0.116	0.531
rs9266658	6	31347644	A	G	62.191	-0.159	0.020	2.68E-15	-0.113	0.059	0.055
rs9271365	6	32586794	G	T	173.773	0.253	0.019	1.44E-39	0.283	0.053	0.000
rs9277910	6	33124052	T	G	43.739	0.137	0.021	3.59E-11	-0.047	0.060	0.436
rs9497965	6	148521292	T	C	22.883	0.082	0.017	1.71E-06	-0.044	0.049	0.371
**Thyroid cancer**											
rs10909839	1	2708430	A	G	30.082	-0.186	0.034	3.16E-08	-0.028	0.027	0.295
rs1111463	6	31269926	C	A	410.063	-1.377	0.068	2.21E-101	0.081	0.032	0.012

rs113198082	16	3881494	C	T	20.712	0.282	0.062	2.92E-06	-0.024	0.048	0.617
rs114484678	6	32215057	C	T	29.652	-0.474	0.087	1.18E-08	-0.027	0.065	0.676
rs11632488	15	80267501	G	A	22.135	0.165	0.035	1.47E-06	-0.001	0.028	0.977
rs12956324	18	67537270	A	C	25.422	0.176	0.035	3.32E-07	-0.032	0.025	0.204
rs13119723	4	123218313	G	A	37.876	-0.271	0.044	2.22E-10	0.034	0.033	0.295
rs138763718	15	36686378	T	A	22.920	0.603	0.126	3.53E-07	0.086	0.087	0.324
rs139010734	6	31974014	T	C	574.410	3.355	0.140	1.98E-154	0.069	0.093	0.457
rs139458638	7	21872295	A	G	20.607	0.481	0.106	1.68E-06	-0.019	0.090	0.832
rs140323264	6	31973462	C	T	62.971	0.619	0.078	1.34E-16	-0.093	0.078	0.229
rs145832854	22	25310129	A	G	27.526	-0.624	0.119	2.58E-08	0.010	0.089	0.912
rs145931087	1	192038977	C	T	20.855	0.365	0.080	2.29E-06	0.037	0.078	0.631
rs150464045	14	89834364	C	T	19.406	0.573	0.130	2.56E-06	-0.143	0.097	0.138
rs1893592	21	43855067	C	A	25.534	-0.202	0.040	1.90E-07	0.038	0.027	0.162
rs228616	4	103579691	A	G	23.226	-0.140	0.029	1.25E-06	-0.022	0.025	0.389
rs231389	2	204634730	T	C	32.808	-0.206	0.036	4.42E-09	-0.016	0.029	0.575
rs2815037	6	39242453	G	A	29.142	-0.178	0.033	6.02E-08	0.012	0.027	0.661
rs3095227	6	31491000	G	A	43.005	0.230	0.035	3.58E-11	0.043	0.028	0.126
rs3117012	6	33095684	G	A	23.072	-0.144	0.030	1.18E-06	0.032	0.025	0.209
rs3128931	6	32971708	A	G	41.731	-0.239	0.037	2.67E-11	0.037	0.028	0.200
rs3131781	6	30937732	G	A	835.569	1.070	0.037	1.00E-200	-0.017	0.035	0.631
rs313839	19	47221557	G	C	29.959	-0.279	0.051	2.12E-08	-0.040	0.033	0.229
rs3184504	12	111884608	C	T	38.639	-0.186	0.030	5.05E-10	-0.023	0.025	0.355
rs34645399	6	32589169	G	A	252.396	0.747	0.047	1.63E-59	-0.054	0.058	0.347
rs36023390	3	71523093	T	C	26.120	-0.184	0.036	2.16E-07	0.004	0.026	0.871
rs4147359	10	6108439	A	G	52.188	0.217	0.030	4.06E-13	-0.039	0.026	0.138
rs428947	6	32195786	A	G	64.068	-0.632	0.079	5.62E-17	-0.002	0.046	0.977
rs444697	6	33575009	A	G	27.196	0.177	0.034	1.07E-07	0.014	0.029	0.631
rs453098	6	31691657	A	G	27.875	-0.338	0.064	4.71E-08	-0.012	0.050	0.813
rs4548024	6	138165744	C	T	27.706	-0.226	0.043	9.63E-08	0.030	0.029	0.302
rs4817988	21	40468838	A	G	58.957	-0.315	0.041	4.20E-15	-0.019	0.028	0.490
rs492602	19	49206417	G	A	20.372	0.135	0.030	3.72E-06	-0.019	0.025	0.437
rs59377618	6	32788137	C	T	36.739	-0.303	0.050	4.39E-10	0.059	0.039	0.129
rs61954179	13	40794504	T	C	25.308	0.262	0.052	2.64E-07	-0.092	0.044	0.035

rs62398260	6	31356234	A	C	61.215	-0.446	0.057	1.03E-15	0.015	0.046	0.741
rs663743	11	64107735	A	G	27.580	-0.168	0.032	8.42E-08	-0.006	0.026	0.813
rs687308	6	32567256	T	C	162.346	-0.624	0.049	2.44E-39	0.019	0.031	0.537
rs725613	16	11169683	G	T	36.011	-0.198	0.033	5.50E-10	0.024	0.026	0.355
rs72837826	2	111933001	T	G	35.484	0.304	0.051	1.26E-09	-0.036	0.041	0.380
rs73045256	19	41811072	G	A	22.650	0.338	0.071	7.13E-07	0.025	0.056	0.661
rs74655177	16	69098821	T	C	21.107	0.299	0.065	2.30E-06	-0.058	0.055	0.288
rs75030813	3	154810318	C	T	21.312	0.503	0.109	1.20E-06	-0.090	0.083	0.275
rs7750271	6	91036225	G	A	24.652	0.213	0.043	3.06E-07	-0.024	0.032	0.447
rs7758790	6	31552850	C	T	133.403	-0.439	0.038	2.51E-32	0.054	0.030	0.071
rs79940565	11	63560994	C	T	27.321	0.763	0.146	2.00E-08	0.099	0.110	0.372
rs9469323	6	32996802	T	C	35.852	0.222	0.037	1.50E-09	-0.049	0.033	0.138
rs9528775	13	65078624	T	C	21.138	0.166	0.036	2.66E-06	-0.012	0.026	0.646
rs9553523	13	25630049	A	G	23.363	-0.222	0.046	6.21E-07	-0.027	0.035	0.437
rs9858213	3	49731861	T	G	83.737	0.284	0.031	2.43E-20	0.042	0.027	0.115

SNP	Chr	Position	Effect Allele	Other Allele	F	Association with exposure			Association with PSC		
						Beta	SE	*P*	Beta	SE	*P*
**Hypothyroidism**											
rs10021756	4	40285931	T	C	23.600	0.006	0.001	1.20E-06	0.130	0.093	0.141
rs10032998	4	80957826	A	G	22.219	-0.002	0.000	2.40E-06	0.010	0.032	0.746
rs10036386	5	76543603	T	C	50.908	0.003	0.000	9.70E-13	-0.043	0.037	0.231
rs10077159	5	142822029	A	T	29.564	-0.003	0.000	5.40E-08	-0.013	0.039	0.741
rs10087240	8	129012574	T	C	27.847	0.002	0.000	1.30E-07	0.010	0.030	0.744
rs10147094	14	99727591	G	A	21.814	0.002	0.000	3.00E-06	0.032	0.035	0.346
rs1032129	8	119977337	C	A	39.530	-0.003	0.000	3.20E-10	-0.044	0.033	0.182
rs10424978	19	4837557	A	C	95.380	-0.004	0.000	1.60E-22	0.043	0.035	0.208
rs10440761	5	108784915	G	A	22.365	0.008	0.002	2.30E-06	-0.082	0.141	0.534
rs1050976	6	408079	T	C	53.308	0.003	0.000	2.90E-13	0.120	0.034	0.000
rs10737048	10	6197459	G	A	23.130	0.006	0.001	1.50E-06	0.212	0.106	0.035
rs10742340	11	35318394	C	T	79.585	0.004	0.000	4.60E-19	-0.001	0.031	0.971

rs10772561	12	12572548	C	T	27.866	0.002	0.000	1.30E-07	0.031	0.031	0.320
rs1079418	6	166060601	G	A	37.013	-0.003	0.000	1.20E-09	0.024	0.034	0.471
rs10818007	9	100498968	C	G	23.466	0.004	0.001	1.30E-06	0.057	0.073	0.417
rs10859679	12	94556678	C	A	31.170	0.003	0.001	2.40E-08	0.083	0.043	0.046
rs1088897	17	8876494	G	A	27.840	0.003	0.001	1.30E-07	-0.065	0.042	0.115
rs10917470	1	19845206	A	G	38.192	0.003	0.000	6.40E-10	0.093	0.029	0.001
rs10930013	2	162070325	A	G	35.637	0.003	0.000	2.40E-09	0.004	0.031	0.908
rs10937477	3	191048462	T	C	27.994	0.003	0.001	1.20E-07	0.051	0.033	0.121
rs10940534	5	56583527	G	T	23.110	-0.002	0.000	1.50E-06	-0.046	0.033	0.164
rs10956412	8	129162497	C	A	50.706	-0.004	0.001	1.10E-12	-0.066	0.040	0.093
rs10974452	9	4315250	C	G	22.213	0.004	0.001	2.40E-06	0.031	0.058	0.581
rs11064860	12	110489208	T	C	27.990	-0.004	0.001	1.20E-07	-0.103	0.045	0.020
rs11073337	15	38847763	C	A	69.817	0.004	0.001	6.50E-17	-0.013	0.036	0.721
rs11177053	12	68499237	C	T	31.438	-0.003	0.000	2.10E-08	0.016	0.030	0.586
rs111915798	8	129210479	A	G	26.263	0.007	0.001	3.00E-07	-0.219	0.095	0.016
rs112165453	2	204664815	T	C	31.053	0.009	0.002	2.50E-08	0.150	0.123	0.195
rs11258303	10	6405534	A	C	52.555	0.004	0.001	4.20E-13	-0.024	0.040	0.554
rs114840990	6	33132935	A	T	21.135	-0.006	0.001	4.30E-06	0.026	0.091	0.768
rs1150258	1	207074905	C	T	23.163	0.002	0.000	1.50E-06	-0.011	0.030	0.716
rs11582506	1	117277777	G	A	25.037	0.002	0.000	5.60E-07	0.075	0.029	0.009
rs116669695	6	32969745	G	A	22.803	0.004	0.001	1.80E-06	-0.023	0.055	0.675
rs11675342	2	1407628	T	C	126.238	0.005	0.000	2.70E-29	0.030	0.031	0.333
rs11692215	2	100746573	T	C	24.001	-0.003	0.001	9.60E-07	-0.015	0.034	0.648
rs117731905	11	18426721	A	G	24.361	-0.008	0.002	8.00E-07	-0.035	0.114	0.741
rs11783023	8	141642392	T	C	40.650	-0.003	0.000	1.80E-10	-0.008	0.038	0.840
rs11822813	11	93912638	G	A	35.598	-0.005	0.001	2.40E-09	-0.072	0.064	0.246
rs11901769	2	160363669	T	A	31.899	0.003	0.001	1.60E-08	-0.024	0.034	0.467
rs11902277	2	204924525	A	G	21.963	-0.005	0.001	2.80E-06	0.059	0.067	0.366
rs11926659	3	105499839	G	A	27.222	-0.003	0.001	1.80E-07	0.054	0.044	0.215
rs11939242	4	20940352	C	A	22.802	-0.004	0.001	1.80E-06	0.050	0.054	0.343
rs1217238	1	114131553	A	G	41.697	-0.003	0.000	1.10E-10	0.041	0.032	0.195
rs12191243	6	135446826	G	C	30.740	0.003	0.001	2.90E-08	0.040	0.035	0.236
rs12271161	11	116979911	A	G	40.733	-0.004	0.001	1.70E-10	-0.071	0.037	0.048

rs12485900	3	192625190	T	C	29.164	0.002	0.000	6.60E-08	-0.052	0.030	0.076
rs12683611	9	101816460	A	G	21.348	0.002	0.001	3.80E-06	0.011	0.037	0.765
rs12697352	5	35837234	A	G	30.977	-0.003	0.000	2.60E-08	-0.046	0.033	0.156
rs12720299	19	10468668	G	C	24.661	0.003	0.001	6.80E-07	0.035	0.048	0.452
rs12722022	6	33048564	A	G	43.184	-0.008	0.001	5.00E-11	-0.524	0.114	0.000
rs12902447	15	86027453	G	A	23.166	-0.002	0.000	1.50E-06	0.011	0.032	0.733
rs12920568	16	9227943	C	A	25.900	0.003	0.001	3.60E-07	0.037	0.038	0.313
rs12981033	19	50197406	G	A	43.720	-0.003	0.000	3.80E-11	0.087	0.036	0.013
rs13076468	3	5022961	C	A	33.923	0.003	0.001	5.70E-09	-0.011	0.041	0.785
rs13090803	3	105937848	T	G	78.620	0.005	0.001	7.50E-19	-0.067	0.037	0.069
rs13299616	9	123594803	C	T	23.530	-0.002	0.000	1.20E-06	-0.048	0.032	0.125
rs13360007	5	156577720	G	A	30.918	0.004	0.001	2.70E-08	-0.016	0.044	0.717
rs1343986	12	103772733	C	G	23.562	-0.004	0.001	1.20E-06	0.052	0.061	0.378
rs1381286	14	98597582	G	C	24.427	0.003	0.001	7.70E-07	0.079	0.038	0.033
rs145269503	6	31705126	A	G	29.801	-0.008	0.002	4.80E-08	-0.421	0.113	0.000
rs1474466	6	112098264	G	A	25.692	-0.002	0.000	4.00E-07	0.053	0.035	0.123
rs1479559	5	76479833	T	C	21.454	-0.003	0.001	3.60E-06	0.061	0.051	0.219
rs149492690	2	68553043	T	A	21.401	0.008	0.002	3.70E-06	-0.031	0.130	0.796
rs1534430	2	12644736	T	C	71.938	-0.004	0.000	2.20E-17	-0.020	0.032	0.521
rs1543603	6	25413922	G	A	21.901	-0.003	0.001	2.90E-06	-0.243	0.039	0.000
rs1549142	19	18387993	T	C	53.886	0.004	0.001	2.10E-13	-0.070	0.040	0.078
rs1561924	8	129569371	A	G	50.125	-0.005	0.001	1.40E-12	-0.023	0.052	0.654
rs1599795	3	119243855	A	T	48.805	0.004	0.001	2.80E-12	-0.089	0.041	0.028
rs17020139	1	108369483	A	G	227.787	0.012	0.001	1.80E-51	0.039	0.052	0.437
rs17129794	1	67794918	C	A	32.791	0.003	0.001	1.00E-08	-0.037	0.079	0.625
rs1723022	1	167408670	T	G	34.863	0.003	0.000	3.50E-09	-0.063	0.036	0.072
rs17306827	2	174763936	A	G	24.122	0.002	0.000	9.00E-07	0.016	0.030	0.587
rs174599	11	61621556	C	G	43.621	-0.003	0.000	4.00E-11	0.027	0.035	0.447
rs1782648	10	81065201	A	G	29.669	0.003	0.000	5.10E-08	-0.017	0.036	0.636
rs1810396	8	133918769	G	A	65.908	-0.004	0.000	4.70E-16	-0.033	0.032	0.292
rs1884352	1	8537289	A	G	26.754	-0.002	0.000	2.30E-07	-0.083	0.032	0.008
rs1926193	10	90760606	C	T	23.003	-0.002	0.000	1.60E-06	0.005	0.035	0.893
rs1985378	22	39678312	G	A	29.311	0.002	0.000	6.20E-08	0.007	0.035	0.838

rs2029751	1	200698286	C	A	41.526	0.004	0.001	1.20E-10	0.044	0.047	0.337
rs2053979	11	47439444	G	A	27.214	0.002	0.000	1.80E-07	0.011	0.031	0.728
rs2111485	2	163110536	G	A	69.140	0.004	0.000	9.20E-17	-0.085	0.034	0.011
rs212411	6	159468565	A	G	27.966	0.002	0.000	1.20E-07	0.099	0.035	0.004
rs2130357	6	27886830	T	C	24.328	-0.003	0.001	8.10E-07	-0.154	0.036	0.000
rs2160316	2	202885167	C	T	25.055	0.002	0.000	5.60E-07	0.030	0.035	0.382
rs221781	7	100295908	G	A	39.915	0.004	0.001	2.70E-10	0.074	0.049	0.124
rs2218245	13	70596378	A	C	21.298	-0.003	0.001	3.90E-06	0.020	0.037	0.588
rs2234167	1	2494330	A	G	42.839	0.004	0.001	5.90E-11	0.014	0.044	0.743
rs2271973	3	100979256	A	C	22.617	0.003	0.001	2.00E-06	-0.013	0.041	0.738
rs2284169	6	30172385	C	A	82.812	-0.006	0.001	9.00E-20	-0.270	0.041	0.000
rs229540	22	37591318	G	T	127.764	0.005	0.000	1.30E-29	0.058	0.029	0.043
rs2333567	4	177730455	C	T	25.469	0.004	0.001	4.50E-07	-0.135	0.045	0.002
rs2392239	1	101735917	T	C	21.879	-0.002	0.000	2.90E-06	0.052	0.033	0.109
rs2412975	22	30540590	C	T	42.952	-0.003	0.000	5.60E-11	-0.022	0.031	0.469
rs2415317	14	36609678	A	G	26.612	-0.002	0.000	2.50E-07	-0.041	0.030	0.166
rs244672	5	133419283	T	C	53.299	-0.005	0.001	2.90E-13	-0.067	0.042	0.107
rs2473808	1	19638883	C	T	43.413	-0.003	0.000	4.40E-11	0.000	0.062	0.994
rs2476601	1	114377568	G	A	770.620	-0.020	0.001	1.30E-169	-0.026	0.094	0.780
rs2481974	1	28517379	A	C	21.828	-0.002	0.000	3.00E-06	-0.076	0.031	0.013
rs2607013	6	31820400	T	C	158.681	0.007	0.001	2.20E-36	-0.227	0.047	0.000
rs2687938	13	50764482	T	C	23.698	0.002	0.000	1.10E-06	-0.057	0.030	0.054
rs2744944	6	34658080	C	A	29.168	-0.002	0.000	6.60E-08	0.015	0.036	0.676
rs28157	5	102597292	T	G	47.803	-0.003	0.000	4.70E-12	0.011	0.032	0.731
rs2823272	21	16799673	A	T	48.105	-0.003	0.000	4.00E-12	-0.031	0.033	0.335
rs28375404	6	32628098	T	C	94.830	-0.011	0.001	2.10E-22	-0.428	0.084	0.000
rs28450181	4	87819369	G	A	34.868	0.003	0.001	3.50E-09	0.017	0.039	0.655
rs2859072	6	32703313	A	G	71.448	-0.004	0.001	2.80E-17	-0.316	0.040	0.000
rs28594633	6	32538682	A	G	409.455	0.011	0.001	4.80E-91	-0.234	0.135	0.065
rs3006986	1	114562181	G	C	22.986	0.003	0.001	1.60E-06	-0.157	0.095	0.083
rs3087243	2	204738919	A	G	370.140	-0.009	0.000	1.70E-82	-0.112	0.030	0.000
rs3093665	6	31545391	C	A	37.391	0.009	0.002	9.70E-10	-0.415	0.119	0.000
rs3184504	12	111884608	C	T	525.303	-0.010	0.000	3.00E-116	-0.186	0.030	0.000

rs337637	4	38604470	A	G	27.461	-0.002	0.000	1.60E-07	-0.083	0.033	0.010
rs34509786	3	12305916	G	T	56.354	0.004	0.001	6.10E-14	-0.049	0.040	0.211
rs347153	3	32472324	C	A	21.504	-0.002	0.000	3.50E-06	-0.014	0.032	0.656
rs34916533	13	33074674	A	C	22.568	-0.002	0.000	2.00E-06	0.035	0.030	0.241
rs367023	2	8451498	G	A	39.218	-0.003	0.000	3.80E-10	0.069	0.033	0.037
rs3735477	7	44714512	C	T	25.313	0.002	0.000	4.90E-07	-0.036	0.035	0.293
rs3784099	14	68749927	A	G	42.864	-0.003	0.000	5.90E-11	-0.023	0.033	0.489
rs3807307	7	128579202	C	T	35.176	0.003	0.000	3.00E-09	0.089	0.034	0.007
rs3850765	10	124139910	C	T	48.478	0.003	0.000	3.30E-12	-0.001	0.032	0.972
rs3862663	11	60753960	A	G	23.814	0.006	0.001	1.10E-06	0.165	0.079	0.031
rs41287542	6	109387021	G	A	25.453	0.005	0.001	4.50E-07	-0.166	0.090	0.054
rs4320727	1	25351581	A	G	39.343	0.003	0.000	3.60E-10	-0.028	0.037	0.446
rs4409785	11	95311422	C	T	134.472	0.007	0.001	4.30E-31	0.032	0.044	0.459
rs4660154	1	235356301	G	A	21.496	-0.002	0.000	3.50E-06	-0.029	0.030	0.344
rs4748400	10	17734360	G	C	26.642	-0.004	0.001	2.40E-07	0.061	0.049	0.203
rs4794063	17	45804494	T	C	61.770	0.004	0.001	3.90E-15	-0.008	0.039	0.832
rs479777	11	64107735	C	T	40.934	-0.003	0.000	1.60E-10	-0.168	0.032	0.000
rs4804433	19	7240776	T	G	35.900	-0.003	0.001	2.10E-09	0.061	0.042	0.134
rs4820437	22	41761290	C	T	29.798	-0.003	0.001	4.80E-08	0.067	0.037	0.065
rs4835536	4	149662857	T	G	139.035	-0.006	0.001	4.30E-32	-0.015	0.037	0.680
rs484959	1	110366083	C	T	39.445	0.003	0.000	3.40E-10	0.011	0.034	0.750
rs4885151	13	74697856	G	T	24.561	0.002	0.000	7.20E-07	0.022	0.032	0.487
rs4922066	8	19456270	T	C	21.092	-0.002	0.000	4.40E-06	-0.005	0.040	0.891
rs55792153	5	138854203	C	A	24.675	0.003	0.001	6.80E-07	-0.038	0.038	0.315
rs55926131	12	115085267	T	C	24.813	0.002	0.000	6.30E-07	-0.078	0.034	0.020
rs56400413	7	77340821	A	T	31.188	0.003	0.000	2.30E-08	0.009	0.035	0.802
rs57465888	6	109593038	G	A	20.964	-0.005	0.001	4.70E-06	-0.120	0.083	0.133
rs57871924	3	46282278	A	T	24.021	0.004	0.001	9.50E-07	-0.099	0.061	0.096
rs57938373	3	39336038	T	C	43.605	0.004	0.001	4.00E-11	-0.001	0.041	0.990
rs58620180	20	62259394	A	G	23.027	0.008	0.002	1.60E-06	0.130	0.101	0.175
rs5865	2	98373006	T	C	43.255	-0.003	0.000	4.80E-11	-0.065	0.030	0.030
rs59036109	13	97990910	A	C	21.500	-0.002	0.000	3.50E-06	-0.054	0.033	0.100
rs59100653	1	21984969	A	G	21.776	0.004	0.001	3.10E-06	0.049	0.065	0.444

rs59183580	11	95425470	G	A	27.428	0.003	0.001	1.60E-07	-0.072	0.052	0.149
rs60600003	7	37382465	G	T	35.984	0.004	0.001	2.00E-09	0.007	0.050	0.894
rs6125457	20	47378799	A	G	24.055	0.002	0.000	9.40E-07	0.050	0.032	0.116
rs61776678	1	38377021	A	G	40.909	-0.003	0.000	1.60E-10	0.016	0.034	0.631
rs61778693	1	38651781	T	G	34.526	-0.003	0.001	4.20E-09	0.014	0.035	0.691
rs61907718	11	128172470	A	G	26.056	0.003	0.001	3.30E-07	-0.107	0.036	0.003
rs62052470	16	85904832	A	G	21.705	-0.004	0.001	3.20E-06	0.036	0.062	0.542
rs62182865	2	191468987	C	T	26.731	0.003	0.001	2.30E-07	-0.072	0.047	0.118
rs6441938	3	46045619	C	G	24.255	0.003	0.001	8.40E-07	0.011	0.037	0.768
rs6452444	5	71685736	C	T	32.656	0.003	0.001	1.10E-08	-0.059	0.034	0.076
rs6505765	18	12782849	G	C	51.283	0.003	0.000	8.00E-13	0.092	0.032	0.004
rs6517444	21	39344121	G	A	24.345	0.002	0.000	8.10E-07	0.048	0.032	0.131
rs6602315	10	8547572	C	A	23.205	-0.003	0.001	1.50E-06	-0.012	0.051	0.804
rs6602392	10	6078079	A	C	24.392	0.004	0.001	7.90E-07	0.079	0.054	0.134
rs66749983	13	43063831	T	A	59.823	0.004	0.000	1.00E-14	0.010	0.032	0.762
rs6681271	1	157666644	C	T	26.064	0.002	0.000	3.30E-07	0.026	0.031	0.404
rs671565	1	24042811	A	G	26.500	-0.002	0.000	2.60E-07	-0.077	0.030	0.010
rs6739788	2	55862355	T	A	32.867	0.006	0.001	9.90E-09	-0.116	0.069	0.082
rs678456	1	160464548	C	T	24.180	-0.004	0.001	8.80E-07	-0.034	0.052	0.497
rs67927699	2	61187415	C	G	21.219	0.002	0.000	4.10E-06	0.135	0.035	0.000
rs6798068	3	108162362	A	G	35.034	0.003	0.000	3.20E-09	0.028	0.033	0.389
rs68189149	17	73820021	T	C	25.882	0.003	0.001	3.60E-07	-0.031	0.049	0.524
rs6819295	4	103358641	A	G	21.547	0.002	0.001	3.50E-06	0.090	0.039	0.018
rs6833591	4	123546282	G	A	37.930	-0.003	0.000	7.30E-10	-0.191	0.032	0.000
rs683763	10	89807680	T	G	25.923	0.002	0.000	3.60E-07	0.036	0.032	0.247
rs6915638	6	30722949	T	A	64.353	0.009	0.001	1.00E-15	-0.209	0.082	0.008
rs693939	18	23650082	T	C	24.089	-0.002	0.000	9.20E-07	-0.031	0.039	0.430
rs6992869	8	61395832	C	T	40.021	0.003	0.000	2.50E-10	-0.016	0.032	0.624
rs7005834	8	134214204	T	C	45.929	-0.003	0.000	1.20E-11	-0.006	0.034	0.853
rs701292	7	83543341	T	C	22.547	0.002	0.000	2.10E-06	-0.033	0.035	0.333
rs7090530	10	6110875	A	C	84.582	0.004	0.000	3.70E-20	0.198	0.032	0.000
rs71508903	10	63781824	T	C	137.360	0.007	0.001	1.00E-31	0.041	0.043	0.335
rs72807396	10	90605565	A	G	22.643	0.007	0.001	2.00E-06	0.257	0.104	0.009

rs72850698	2	145417530	C	G	21.839	0.004	0.001	3.00E-06	-0.046	0.056	0.400
rs7297415	12	112661104	A	G	25.908	-0.004	0.001	3.60E-07	-0.018	0.050	0.719
rs731151	17	40279903	A	G	49.110	0.004	0.001	2.40E-12	0.010	0.038	0.798
rs7314285	12	111522026	G	T	23.337	-0.004	0.001	1.40E-06	0.040	0.070	0.556
rs739091	22	40317126	A	C	23.810	-0.002	0.000	1.10E-06	0.029	0.035	0.409
rs7417283	1	108303686	T	C	40.199	0.004	0.001	2.30E-10	0.026	0.038	0.491
rs7441808	4	26090375	G	A	65.867	0.004	0.000	4.80E-16	0.016	0.032	0.607
rs74435468	1	65455166	C	G	27.643	0.005	0.001	1.50E-07	0.138	0.056	0.011
rs7575113	2	181885295	C	G	26.073	0.002	0.000	3.30E-07	0.063	0.033	0.053
rs7581413	2	145058878	A	T	23.029	-0.002	0.000	1.60E-06	-0.054	0.035	0.125
rs7582694	2	191970120	G	C	163.912	-0.007	0.001	1.60E-37	-0.041	0.036	0.249
rs7596240	2	242444173	G	A	33.794	0.003	0.000	6.10E-09	0.048	0.034	0.152
rs761357	6	135909796	T	A	39.412	0.003	0.000	3.40E-10	-0.014	0.030	0.647
rs7620353	3	150947555	T	C	23.915	-0.003	0.001	1.00E-06	-0.044	0.046	0.330
rs7649344	3	37011026	C	T	34.603	-0.003	0.000	4.00E-09	-0.048	0.030	0.106
rs76930710	12	68434459	C	T	30.056	-0.006	0.001	4.20E-08	0.061	0.078	0.413
rs76946320	2	12485667	C	T	22.116	0.006	0.001	2.60E-06	-0.031	0.086	0.709
rs7710070	5	10596269	T	G	20.994	-0.002	0.000	4.60E-06	0.053	0.030	0.078
rs772920	12	56390364	G	C	58.601	0.004	0.000	1.90E-14	0.003	0.037	0.943
rs7746336	6	31272554	T	C	26.823	0.004	0.001	2.20E-07	-0.213	0.058	0.000
rs7822344	8	117418375	T	C	21.115	0.003	0.001	4.30E-06	-0.010	0.035	0.781
rs78328982	3	121734641	A	G	44.269	0.006	0.001	2.90E-11	0.070	0.071	0.305
rs7850258	9	100549013	G	A	443.462	0.010	0.000	1.90E-98	-0.056	0.033	0.086
rs7936397	11	577534	A	G	34.844	-0.003	0.000	3.60E-09	-0.023	0.039	0.554
rs7955910	12	122668326	T	G	22.335	0.002	0.000	2.30E-06	-0.036	0.036	0.303
rs79708723	6	148533113	A	G	34.184	0.007	0.001	5.00E-09	0.142	0.085	0.082
rs8047543	16	10956188	G	C	23.358	-0.002	0.001	1.30E-06	-0.006	0.035	0.863
rs8054578	16	79316815	G	A	41.392	-0.003	0.001	1.20E-10	0.046	0.036	0.184
rs897586	8	128193294	A	G	56.706	-0.003	0.000	5.10E-14	-0.099	0.036	0.005
rs916963	7	26146351	A	G	25.120	-0.003	0.001	5.40E-07	0.115	0.037	0.002
rs926103	1	156784982	C	T	28.894	-0.003	0.000	7.60E-08	0.050	0.036	0.164
rs9277627	6	33081979	C	G	258.406	0.010	0.001	3.80E-58	-0.080	0.041	0.047
rs9288593	2	225756496	G	C	24.710	-0.002	0.000	6.70E-07	-0.016	0.037	0.663

rs9291444	4	10713674	T	C	71.146	0.004	0.000	3.30E-17	0.142	0.029	0.000
rs9296079	6	33080173	T	G	68.222	-0.011	0.001	1.50E-16	0.128	0.086	0.120
rs9357120	6	31230717	A	T	336.077	-0.009	0.000	4.60E-75	-0.366	0.034	0.000
rs9358913	6	26239404	G	A	25.005	-0.003	0.001	5.70E-07	-0.117	0.038	0.002
rs9380522	6	35537964	T	C	43.490	0.003	0.000	4.30E-11	-0.016	0.039	0.670
rs9497965	6	148521292	T	C	74.586	0.004	0.000	5.80E-18	-0.001	0.035	0.977
rs9521838	13	111206226	A	G	29.471	-0.003	0.001	5.70E-08	0.030	0.069	0.654
rs952579	6	21884440	A	G	22.481	0.003	0.001	2.10E-06	0.068	0.047	0.142
rs9557168	13	99807350	A	G	30.824	-0.003	0.001	2.80E-08	-0.107	0.039	0.006
rs9619183	22	31844270	T	G	26.406	-0.005	0.001	2.80E-07	-0.031	0.068	0.627
rs9697210	9	131468740	A	G	34.201	-0.004	0.001	5.00E-09	-0.062	0.048	0.187
rs970987	9	21587447	A	C	68.962	-0.004	0.000	1.00E-16	0.044	0.038	0.231
rs979543	15	84352564	A	C	22.985	0.003	0.001	1.60E-06	-0.003	0.038	0.928
rs9831283	3	149008625	A	G	27.629	0.006	0.001	1.50E-07	-0.083	0.090	0.334
rs9921917	16	57379465	T	C	23.519	-0.004	0.001	1.20E-06	0.028	0.069	0.679
**Hyperthyroidism**											
rs10087240	8	129012574	T	C	34.986	0.001	0.000	3.30E-09	0.010	0.030	0.744
rs112335954	6	32539700	A	T	119.739	0.003	0.000	7.20E-28	-0.339	0.158	0.020
rs12741781	1	243433654	G	T	34.502	0.001	0.000	4.30E-09	-0.015	0.031	0.628
rs13250295	8	134212652	T	C	31.636	-0.001	0.000	1.90E-08	-0.031	0.039	0.403
rs1559810	3	188124354	A	C	30.758	0.001	0.000	2.90E-08	0.023	0.030	0.438
rs1794279	6	32667595	T	G	606.488	0.007	0.000	6.50E-134	1.092	0.044	0.000
rs2160215	14	81461472	C	T	200.417	0.003	0.000	1.70E-45	0.031	0.032	0.306
rs2394186	6	29816421	G	A	43.947	-0.002	0.000	3.40E-11	-0.076	0.047	0.097
rs2523590	6	31327064	C	T	176.307	-0.003	0.000	3.10E-40	-0.418	0.035	0.000
rs28752803	6	31300249	T	C	119.857	0.003	0.000	6.80E-28	0.222	0.036	0.000
rs3087243	2	204738919	A	G	101.401	-0.002	0.000	7.50E-24	-0.112	0.030	0.000
rs3129294	6	33084671	C	A	51.643	-0.001	0.000	6.70E-13	-0.151	0.030	0.000
rs409602	5	156608284	A	T	29.866	0.001	0.000	4.60E-08	-0.031	0.043	0.474
rs4409785	11	95311422	C	T	37.480	0.001	0.000	9.20E-10	0.032	0.044	0.459
rs6679677	1	114377568	A	C	68.239	0.002	0.000	1.40E-16	0.026	0.094	0.780
rs6906566	6	31097820	T	C	85.245	-0.002	0.000	2.60E-20	-0.315	0.043	0.000
rs7090530	10	6110875	A	C	34.383	0.001	0.000	4.50E-09	0.198	0.032	0.000

HT

rs10424978	19	4837557	A	C	23.998	-0.082	0.017	8.97E-07	0.043	0.035	0.208
rs10880930	12	46574248	T	C	21.110	-0.085	0.019	4.26E-06	0.021	0.037	0.577
rs1115587	10	64038901	A	G	27.278	-0.096	0.018	1.82E-07	0.017	0.030	0.583
rs11211645	2	1417199	G	A	28.530	0.089	0.017	9.33E-08	0.018	0.031	0.560
rs118075239	13	43458697	C	T	21.564	0.385	0.083	3.45E-06	0.171	0.159	0.245
rs11822254	11	86073315	G	A	21.418	-0.080	0.017	3.52E-06	0.013	0.037	0.715
rs11889341	2	191943742	T	C	42.489	0.125	0.019	6.64E-11	0.040	0.036	0.267
rs12517193	5	76008682	A	G	25.244	0.082	0.016	4.90E-07	-0.054	0.034	0.104
rs1317983	6	43806335	C	T	27.317	0.095	0.018	1.65E-07	0.026	0.037	0.475
rs13249705	8	141652465	G	A	25.712	-0.093	0.018	3.78E-07	0.027	0.044	0.544
rs146346485	6	32263873	A	G	34.942	-0.253	0.043	3.34E-09	-0.343	0.126	0.004
rs150619443	10	93808024	C	G	25.252	0.440	0.088	5.08E-07	-0.364	0.198	0.044
rs1534430	2	12644736	T	C	28.509	-0.088	0.017	9.40E-08	-0.020	0.032	0.521
rs1611499	6	29908111	T	C	22.323	-0.323	0.068	2.27E-06	-0.265	0.061	0.000
rs17490285	14	72279703	G	A	24.028	-0.185	0.038	9.26E-07	-0.018	0.046	0.695
rs17675149	12	63009635	A	G	26.709	0.240	0.046	2.31E-07	-0.019	0.102	0.848
rs17687621	9	100721555	T	C	23.163	-0.105	0.022	1.47E-06	0.051	0.050	0.296
rs1887414	6	109292736	C	T	27.142	0.117	0.022	1.97E-07	-0.171	0.046	0.000
rs2072109	6	30070275	G	C	33.205	-0.187	0.032	8.59E-09	-0.218	0.053	0.000
rs2111485	2	163110536	G	A	21.281	0.078	0.017	4.19E-06	-0.085	0.034	0.011
rs229533	22	37591318	C	A	23.934	0.082	0.017	9.26E-07	0.058	0.029	0.043
rs2347446	22	29144893	C	T	22.549	0.083	0.018	2.15E-06	0.031	0.031	0.327
rs2476601	1	114377568	G	A	199.848	-0.331	0.023	3.49E-45	-0.026	0.094	0.780
rs2802509	13	40966599	C	T	20.875	0.080	0.017	4.67E-06	-0.009	0.032	0.770
rs28367132	4	149639865	A	G	28.016	-0.136	0.026	1.21E-07	-0.003	0.038	0.926
rs3131627	6	31418124	G	A	21.833	0.079	0.017	3.14E-06	0.319	0.029	0.000
rs3184504	12	111884608	C	T	109.576	-0.179	0.017	1.40E-25	-0.186	0.030	0.000
rs34545402	5	41881226	A	C	20.858	0.075	0.016	4.82E-06	-0.015	0.034	0.656
rs34636506	2	204694263	G	A	58.488	-0.135	0.018	1.92E-14	-0.112	0.030	0.000
rs61830417	10	11102913	G	A	21.489	0.274	0.059	3.59E-06	0.005	0.138	0.971
rs62076103	17	45888374	G	A	27.999	-0.185	0.035	1.23E-07	-0.015	0.073	0.838
rs6448432	4	26098810	A	G	22.816	0.090	0.019	1.75E-06	0.011	0.032	0.736

rs7030280	9	100537577	T	C	134.272	0.205	0.018	6.44E-31	-0.055	0.033	0.094
rs73192661	3	188128794	T	C	36.574	-0.102	0.017	1.45E-09	0.024	0.034	0.481
rs7335748	13	89319468	G	A	22.228	0.377	0.080	2.41E-06	-0.143	0.116	0.191
rs7861040	9	127044135	C	G	24.073	0.084	0.017	9.08E-07	-0.006	0.032	0.850
rs79219789	11	69065730	C	G	21.948	-0.155	0.033	2.84E-06	0.000	0.091	0.998
rs80173139	1	108365138	G	A	75.797	0.213	0.025	3.01E-18	0.039	0.052	0.438
rs8084503	18	71061287	T	C	27.079	0.138	0.027	1.97E-07	0.029	0.054	0.585
rs847803	2	64982415	G	A	24.459	0.091	0.018	7.91E-07	0.033	0.033	0.308
rs9266658	6	31347644	A	G	62.191	-0.159	0.020	2.68E-15	-0.206	0.040	0.000
rs9497965	6	148521292	T	C	22.883	0.082	0.017	1.71E-06	-0.001	0.035	0.977
**Thyroid cancer**											
rs10948917	6	55274927	G	C	20.882	-0.184	0.040	4.92E-06	-0.070	0.033	0.033
rs11251457	10	2761680	A	G	22.371	-0.485	0.103	2.27E-06	0.099	0.082	0.210
rs116332743	4	157038116	C	T	23.283	-0.489	0.101	1.40E-06	-0.040	0.049	0.404
rs146668343	4	113640879	T	A	24.618	0.815	0.164	7.02E-07	-0.083	0.081	0.294
rs16857609	2	218296508	T	C	54.243	0.303	0.041	1.81E-13	0.023	0.035	0.505
rs559534	8	8596181	T	C	27.756	-0.243	0.046	1.39E-07	0.016	0.031	0.611
rs72743467	15	67451410	C	A	25.108	0.280	0.056	5.41E-07	0.054	0.046	0.221
rs75423089	12	102456819	T	C	21.472	0.521	0.113	3.61E-06	-0.105	0.135	0.407
rs925489	9	100550455	T	C	151.388	-0.559	0.045	8.77E-35	-0.055	0.033	0.091
rs9642727	8	32414032	C	A	35.099	0.235	0.040	3.25E-09	0.007	0.032	0.835

SNP	Chr	Position	Effect Allele	Other Allele	F	Association with exposure			Association with PBC		
						Beta	SE	*P*	Beta	SE	*P*
**Hypothyroidism**											
rs10036386	5	76543603	T	C	50.908	0.003	0.000	9.70E-13	0.017	0.022	0.445
rs1032129	8	119951900	C	A	39.530	-0.003	0.000	3.20E-10	-0.053	0.022	0.017
rs1050976	6	408079	T	C	53.308	0.003	0.000	2.90E-13	0.061	0.022	0.005
rs10742340	11	35317712	C	T	79.585	0.004	0.000	4.60E-19	0.024	0.022	0.291
rs1079418	6	166047034	G	A	37.013	-0.003	0.000	1.20E-09	0.022	0.023	0.359
rs10859679	12	94556678	C	A	31.170	0.003	0.001	2.40E-08	-0.055	0.031	0.075

rs10917470	1	19845206	A	G	38.192	0.003	0.000	6.40E-10	-0.001	0.021	0.948
rs10956412	8	129165198	C	A	50.706	-0.004	0.001	1.10E-12	0.071	0.030	0.016
rs11073337	15	38847763	C	A	69.817	0.004	0.001	6.50E-17	0.078	0.024	0.001
rs11177053	12	68499237	C	T	31.438	-0.003	0.000	2.10E-08	-0.067	0.023	0.003
rs112165453	2	204664815	T	C	31.053	0.009	0.002	2.50E-08	-0.057	0.122	0.639
rs11256448	10	6079479	G	A	44.083	0.003	0.001	3.10E-11	-0.045	0.026	0.084
rs114077552	6	30254253	C	A	49.161	-0.012	0.002	2.40E-12	-0.138	0.112	0.220
rs116229144	6	31236800	A	G	78.313	0.015	0.002	8.80E-19	0.319	0.125	0.011
rs11675342	2	1407628	T	C	126.238	0.005	0.000	2.70E-29	0.023	0.022	0.297
rs11783023	8	141639262	T	C	40.650	-0.003	0.000	1.80E-10	0.018	0.025	0.476
rs11822813	11	93912638	G	A	35.598	-0.005	0.001	2.40E-09	-0.041	0.041	0.320
rs1217238	1	114134025	A	G	41.697	-0.003	0.000	1.10E-10	-0.019	0.025	0.450
rs12271161	11	116979911	A	G	40.733	-0.004	0.001	1.70E-10	-0.037	0.027	0.172
rs12349571	9	127093743	G	A	31.999	-0.003	0.000	1.50E-08	0.063	0.023	0.005
rs1257926	14	98692996	A	G	38.776	0.003	0.000	4.80E-10	-0.006	0.022	0.771
rs12634152	3	188124354	T	C	213.512	-0.007	0.000	2.40E-48	-0.027	0.025	0.276
rs12697352	5	35874575	A	G	30.977	-0.003	0.000	2.60E-08	-0.222	0.025	0.000
rs12722022	6	33048564	A	G	43.184	-0.008	0.001	5.00E-11	0.215	0.057	0.000
rs12981033	19	50197406	G	A	43.720	-0.003	0.000	3.80E-11	-0.004	0.020	0.851
rs13090803	3	105937848	T	G	78.620	0.005	0.001	7.50E-19	0.008	0.018	0.652
rs13360007	5	156577720	G	A	30.918	0.004	0.001	2.70E-08	0.041	0.031	0.184
rs138453996	16	67349478	A	G	43.944	0.010	0.002	3.40E-11	0.002	0.123	0.989
rs145269503	6	31705126	A	G	29.801	-0.008	0.002	4.80E-08	-0.012	0.068	0.865
rs1534430	2	12644736	T	C	71.938	-0.004	0.000	2.20E-17	-0.034	0.021	0.094
rs1561924	8	129569371	A	G	50.125	-0.005	0.001	1.40E-12	-0.028	0.032	0.382
rs17020139	1	108369483	A	G	227.787	0.012	0.001	1.80E-51	-0.057	0.037	0.121
rs17129794	1	67825399	C	A	32.791	0.003	0.001	1.00E-08	0.433	0.026	0.000
rs1723022	1	167408670	T	G	34.863	0.003	0.000	3.50E-09	0.005	0.024	0.842
rs2029751	1	200730635	C	A	41.526	0.004	0.001	1.20E-10	0.050	0.032	0.120
rs2111485	2	163110536	G	A	69.140	0.004	0.000	9.20E-17	-0.080	0.022	0.000
rs221781	7	100312724	G	A	39.915	0.004	0.001	2.70E-10	0.032	0.037	0.391
rs2284169	6	30172385	C	A	82.812	-0.006	0.001	9.00E-20	-0.049	0.030	0.100
rs229540	22	37591290	G	T	127.764	0.005	0.000	1.30E-29	0.040	0.021	0.065

rs2412975	22	30540590	C	T	42.952	-0.003	0.000	5.60E-11	-0.061	0.022	0.005
rs244672	5	133419283	T	C	53.299	-0.005	0.001	2.90E-13	-0.023	0.032	0.463
rs2473808	1	19638883	C	T	43.413	-0.003	0.000	4.40E-11	-0.006	0.024	0.797
rs2476601	1	114377568	G	A	770.620	-0.020	0.001	1.30E-169	-0.120	0.035	0.001
rs28157	5	102595837	T	G	47.803	-0.003	0.000	4.70E-12	-0.110	0.024	0.000
rs28450181	4	87819369	G	A	34.868	0.003	0.001	3.50E-09	0.017	0.029	0.556
rs3087243	2	204738919	A	G	370.140	-0.009	0.000	1.70E-82	-0.086	0.022	0.000
rs367023	2	8450123	G	A	39.218	-0.003	0.000	3.80E-10	-0.106	0.024	0.000
rs3775291	4	187004074	T	C	69.132	-0.004	0.000	9.20E-17	0.002	0.026	0.923
rs3784099	14	68749927	A	G	42.864	-0.003	0.000	5.90E-11	-0.203	0.024	0.000
rs3807307	7	128579202	C	T	35.176	0.003	0.000	3.00E-09	0.259	0.022	0.000
rs3850765	10	124139910	C	T	48.478	0.003	0.000	3.30E-12	0.017	0.023	0.440
rs4409785	11	95311422	C	T	134.472	0.007	0.001	4.30E-31	-0.005	0.029	0.868
rs4794063	17	45804494	T	C	61.770	0.004	0.001	3.90E-15	0.045	0.025	0.068
rs479777	11	64107477	C	T	40.934	-0.003	0.000	1.60E-10	-0.144	0.024	0.000
rs4820437	22	41761290	C	T	29.798	-0.003	0.001	4.80E-08	0.107	0.026	0.000
rs4835536	4	149662857	T	G	139.035	-0.006	0.001	4.30E-32	-0.003	0.026	0.916
rs484959	1	110366083	C	T	39.445	0.003	0.000	3.40E-10	-0.021	0.022	0.361
rs57938373	3	39336038	T	C	43.605	0.004	0.001	4.00E-11	0.075	0.029	0.009
rs60600003	7	37382465	G	T	35.984	0.004	0.001	2.00E-09	0.253	0.035	0.000
rs6452444	5	71685736	C	T	32.656	0.003	0.001	1.10E-08	-0.001	0.018	0.967
rs6798068	3	108162362	A	G	35.034	0.003	0.000	3.20E-09	-0.025	0.021	0.247
rs6833591	4	123546282	G	A	37.930	-0.003	0.000	7.30E-10	0.012	0.023	0.610
rs6992869	8	61436559	C	T	40.021	0.003	0.000	2.50E-10	0.064	0.022	0.004
rs7005834	8	134214204	T	C	45.929	-0.003	0.000	1.20E-11	0.027	0.024	0.261
rs7090530	10	6110875	A	C	84.582	0.004	0.000	3.70E-20	-0.035	0.022	0.114
rs71508903	10	63789018	T	C	137.360	0.007	0.001	1.00E-31	0.077	0.025	0.002
rs731151	17	40279903	A	G	49.110	0.004	0.001	2.40E-12	0.009	0.033	0.779
rs7417283	1	108329926	T	C	40.199	0.004	0.001	2.30E-10	-0.016	0.029	0.572
rs7441808	4	26090375	G	A	65.867	0.004	0.000	4.80E-16	0.025	0.024	0.304
rs7583027	2	62544391	C	A	39.473	0.003	0.000	3.30E-10	-0.036	0.022	0.105
rs7649344	3	37006396	C	T	34.603	-0.003	0.000	4.00E-09	0.009	0.021	0.680
rs76930710	12	68434459	C	T	30.056	-0.006	0.001	4.20E-08	-0.167	0.069	0.015

rs7762909	6	31321578	G	A	159.729	0.006	0.000	1.30E-36	0.077	0.023	0.001
rs78328982	3	121734641	A	G	44.269	0.006	0.001	2.90E-11	-0.084	0.051	0.096
rs7850258	9	100549013	G	A	443.462	0.010	0.000	1.90E-98	0.022	0.023	0.346
rs7936397	11	612355	A	G	34.844	-0.003	0.000	3.60E-09	-0.110	0.025	0.000
rs79708723	6	148533113	A	G	34.184	0.007	0.001	5.00E-09	0.007	0.067	0.922
rs8054578	16	79316815	G	A	41.392	-0.003	0.001	1.20E-10	-0.012	0.026	0.636
rs897586	8	128193294	A	G	56.706	-0.003	0.000	5.10E-14	0.062	0.022	0.005
rs9296079	6	33072539	T	G	68.222	-0.011	0.001	1.50E-16	-0.162	0.072	0.024
rs9347170	6	167405187	T	C	143.603	-0.006	0.000	4.30E-33	-0.026	0.023	0.264
rs9380522	6	35537964	T	C	43.490	0.003	0.000	4.30E-11	-0.031	0.023	0.176
rs9557168	13	99807350	A	G	30.824	-0.003	0.001	2.80E-08	-0.099	0.028	0.000
**Hyperthyroidism**											
rs10087240	8	129012574	T	C	34.986	0.001	0.000	3.30E-09	-0.023	0.022	0.300
rs10195156	2	168461673	C	T	21.290	0.001	0.000	3.90E-06	-0.002	0.025	0.926
rs11642757	16	79753467	A	G	27.555	0.001	0.000	1.50E-07	-0.060	0.025	0.015
rs11736377	4	40301264	T	C	27.837	-0.001	0.000	1.30E-07	0.009	0.023	0.694
rs12741781	1	243458502	G	T	34.502	0.001	0.000	4.30E-09	0.023	0.020	0.255
rs13250295	8	134212652	T	C	31.636	-0.001	0.000	1.90E-08	0.021	0.024	0.380
rs1559810	3	188124354	A	C	30.758	0.001	0.000	2.90E-08	0.027	0.025	0.276
rs17651741	15	38899088	A	G	21.627	0.001	0.000	3.30E-06	0.115	0.026	0.000
rs17664732	8	32437118	C	T	23.273	0.001	0.000	1.40E-06	-0.039	0.025	0.122
rs1794279	6	32667595	T	G	606.488	0.007	0.000	6.50E-134	-0.056	0.037	0.128
rs2074466	6	29408313	A	C	25.108	-0.001	0.000	5.40E-07	0.020	0.028	0.472
rs2160215	14	81461472	C	T	200.417	0.003	0.000	1.70E-45	-0.019	0.023	0.410
rs2394186	6	29816421	G	A	43.947	-0.002	0.000	3.40E-11	-0.016	0.032	0.620
rs2523590	6	31327064	C	T	176.307	-0.003	0.000	3.10E-40	-0.031	0.023	0.186
rs2523998	6	29914548	G	T	21.344	-0.001	0.000	3.80E-06	0.014	0.030	0.648
rs3087243	2	204738919	A	G	101.401	-0.002	0.000	7.50E-24	-0.086	0.022	0.000
rs3129294	6	33084671	C	A	51.643	-0.001	0.000	6.70E-13	0.277	0.022	0.000
rs4409785	11	95311422	C	T	37.480	0.001	0.000	9.20E-10	-0.005	0.029	0.868
rs444210	6	167390242	G	A	25.076	0.001	0.000	5.50E-07	0.043	0.022	0.046
rs56079671	7	139466379	A	G	21.445	-0.001	0.000	3.60E-06	-0.054	0.044	0.225
rs6427397	1	157734907	T	C	24.154	0.001	0.000	8.90E-07	-0.094	0.022	0.000

rs6679677	1	114303808	A	C	68.239	0.002	0.000	1.40E-16	0.113	0.036	0.002
rs6906566	6	31097820	T	C	85.245	-0.002	0.000	2.60E-20	-0.011	0.031	0.717
rs6911639	6	32978178	C	T	23.572	-0.001	0.000	1.20E-06	0.038	0.025	0.130
rs7090530	10	6110875	A	C	34.383	0.001	0.000	4.50E-09	-0.035	0.022	0.114
rs71508903	10	63789018	T	C	26.012	0.001	0.000	3.40E-07	0.077	0.025	0.002
**HT**											
rs10880930	12	46574248	T	C	21.110	-0.085	0.019	4.26E-06	0.001	0.026	0.973
rs111358861	4	106712204	G	A	21.632	1.820	0.391	3.30E-06	-0.159	0.208	0.444
rs11211645	2	1412581	G	A	28.530	0.089	0.017	9.33E-08	0.019	0.022	0.391
rs118075239	13	43458697	C	T	21.564	0.385	0.083	3.45E-06	-0.087	0.097	0.369
rs11822254	11	86073315	G	A	21.418	-0.080	0.017	3.52E-06	-0.034	0.023	0.129
rs11889341	2	191943742	T	C	42.489	0.125	0.019	6.64E-11	0.238	0.025	0.000
rs12517193	5	76008682	A	G	25.244	0.082	0.016	4.90E-07	-0.006	0.017	0.719
rs1317983	6	43814190	C	T	27.317	0.095	0.018	1.65E-07	0.009	0.025	0.718
rs13249705	8	141649551	G	A	25.712	-0.093	0.018	3.78E-07	0.005	0.028	0.866
rs146346485	6	32263873	A	G	34.942	-0.253	0.043	3.34E-09	0.181	0.084	0.031
rs1534430	2	12644736	T	C	28.509	-0.088	0.017	9.40E-08	-0.034	0.021	0.094
rs1611499	6	29910419	T	C	22.323	-0.323	0.068	2.27E-06	0.024	0.035	0.489
rs17490285	14	72279703	G	A	24.028	-0.185	0.038	9.26E-07	0.052	0.035	0.132
rs17675149	12	63009635	A	G	26.709	0.240	0.046	2.31E-07	-0.044	0.080	0.586
rs17687621	9	100721871	T	C	23.163	-0.105	0.022	1.47E-06	0.010	0.038	0.784
rs1887414	6	109292736	C	T	27.142	0.117	0.022	1.97E-07	-0.011	0.029	0.710
rs2111485	2	163110536	G	A	21.281	0.078	0.017	4.19E-06	-0.080	0.022	0.000
rs229533	22	37587111	C	A	23.934	0.082	0.017	9.26E-07	0.040	0.021	0.062
rs2347446	22	29160314	C	T	22.549	0.083	0.018	2.15E-06	0.035	0.021	0.100
rs2476601	1	114377568	G	A	199.848	-0.331	0.023	3.49E-45	-0.120	0.035	0.001
rs2802509	13	40966599	C	T	20.875	0.080	0.017	4.67E-06	0.021	0.026	0.416
rs28367132	4	149639865	A	G	28.016	-0.136	0.026	1.21E-07	-0.003	0.029	0.903
rs3131627	6	31415065	G	A	21.833	0.079	0.017	3.14E-06	0.041	0.021	0.053
rs3184504	12	111910219	C	T	109.576	-0.179	0.017	1.40E-25	-0.191	0.021	0.000
rs34636506	2	204692216	G	A	58.488	-0.135	0.018	1.92E-14	-0.096	0.022	0.000
rs62076103	17	45888374	G	A	27.999	-0.185	0.035	1.23E-07	-0.148	0.054	0.007
rs6448432	4	26098810	A	G	22.816	0.090	0.019	1.75E-06	0.028	0.024	0.238

rs7030280	9	100537577	T	C	134.272	0.205	0.018	6.44E-31	0.029	0.024	0.221
rs72814592	10	72501497	C	T	24.303	-0.344	0.070	8.12E-07	0.065	0.058	0.265
rs7335748	13	89319468	G	A	22.228	0.377	0.080	2.41E-06	0.033	0.080	0.685
rs80173139	1	108365138	G	A	75.797	0.213	0.025	3.01E-18	-0.055	0.037	0.136
rs8084503	18	71061287	T	C	27.079	0.138	0.027	1.97E-07	0.032	0.034	0.354
rs9266658	6	31347644	A	G	62.191	-0.159	0.020	2.68E-15	0.056	0.031	0.073
rs9277910	6	33124052	T	G	43.739	0.137	0.021	3.59E-11	-0.100	0.028	0.000
**Thyroid cancer**											
rs116332743	4	157038116	C	T	23.283	-0.489	0.101	1.40E-06	-0.038	0.035	0.285
rs12112897	7	78537885	A	G	23.150	0.378	0.079	1.50E-06	0.043	0.037	0.254
rs16857609	2	218296508	T	C	54.243	0.303	0.041	1.81E-13	0.015	0.019	0.445
rs559534	8	8596181	T	C	27.756	-0.243	0.046	1.39E-07	-0.009	0.018	0.606
rs75423089	12	102579574	T	C	21.472	0.521	0.113	3.61E-06	-0.014	0.103	0.896
rs925489	9	100546600	T	C	151.388	-0.559	0.045	8.77E-35	0.022	0.023	0.344
rs9642727	8	32414032	C	A	35.099	0.235	0.040	3.25E-09	0.006	0.019	0.761

SNP: single nucleotide polymorphism; AIH: Autoimmune Hepatitis; PBC: Primary Biliary Cholangitis; PSC: Primary Sclerosing Cholangitis; CHC: chronic hepatitis C; HT: Hashimoto's thyroiditis; SE: standard error;
