# Supplementary material for: Exploring causal links between autoimmune liver diseases, chronic hepatitis C, and thyroid disorders: Evidence from NHANES and GWAS studies
Source: Medicine (Baltimore). 2025 Aug 29;104(35):e44112. doi: 10.1097/MD.0000000000044112 (PMC12401383; doi:10.1097/MD.0000000000044112)
Supplement: Supplementary file 2 [file medi-104-e44112-s002.pdf]

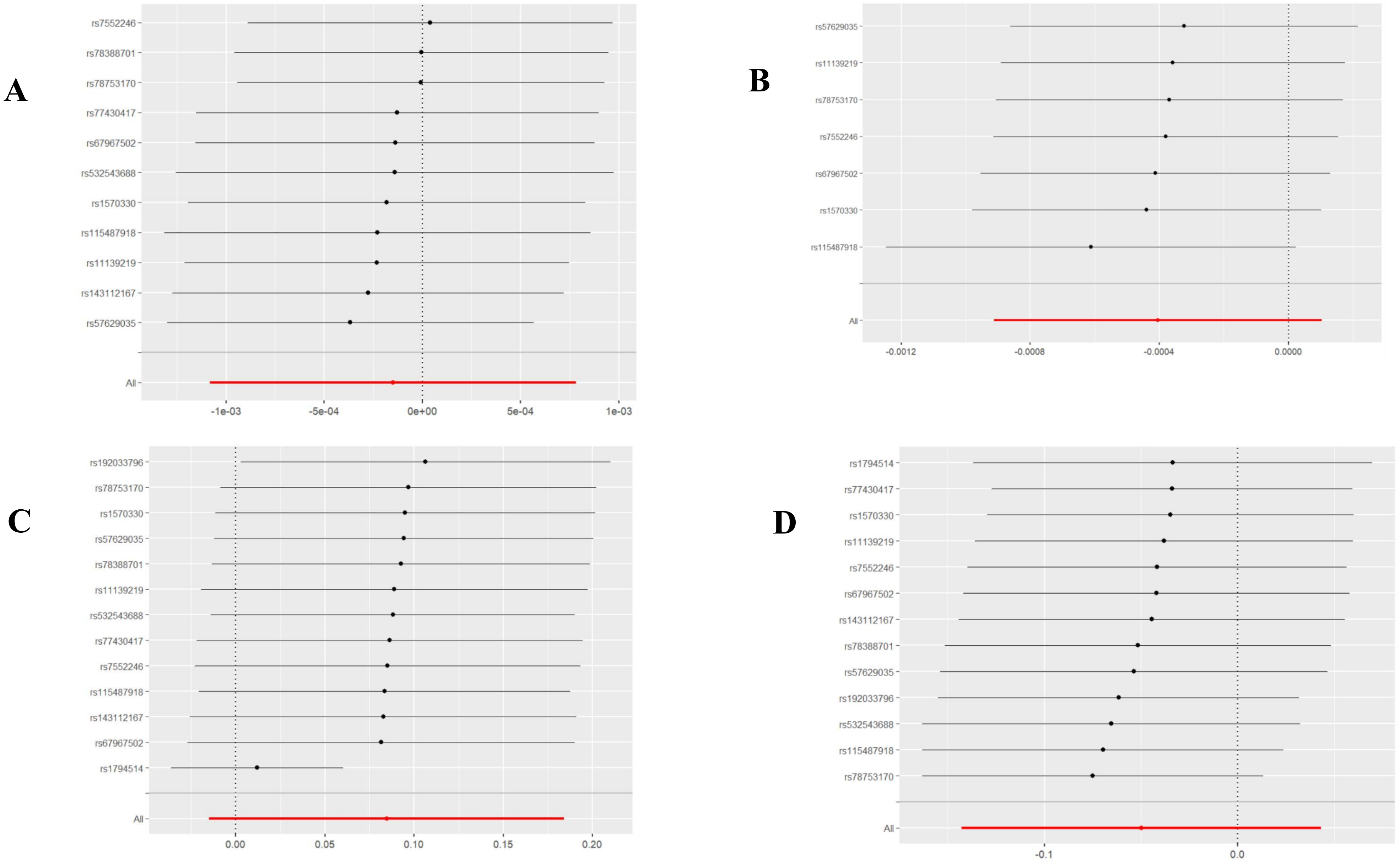

Figure S1. Leave-one-out analysis for the effect of AIH and thyroid diseases. (A) Hypothyroidism, (B) Hyperthyroidism, (C) Hashimoto' thyroiditis and (D) Thyroid cancer

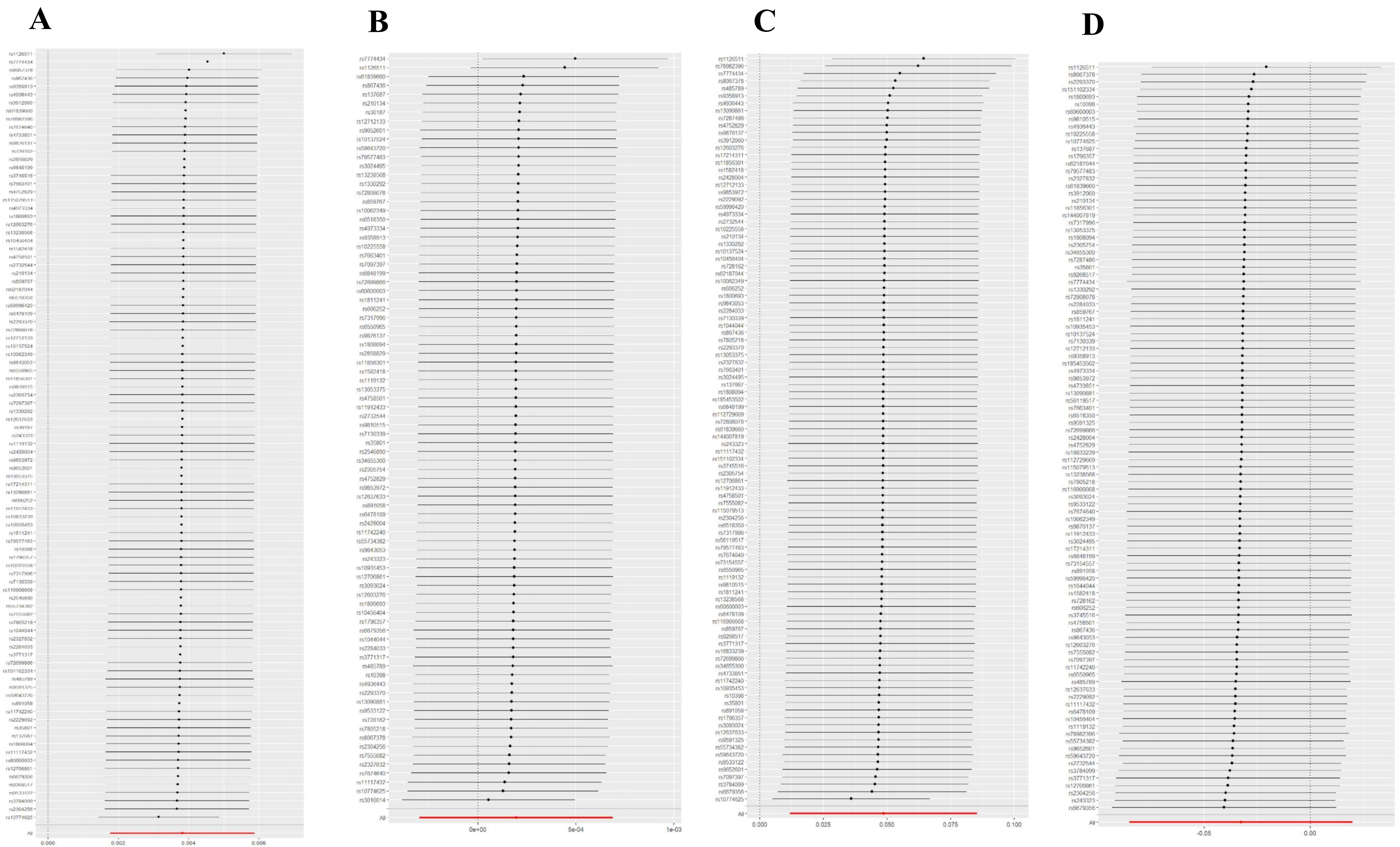

Figure S2. Leave-one-out analysis for the effect of PBC and thyroid diseases. (A) Hypothyroidism, (B) Hyperthyroidism, (C) Hashimoto' thyroiditis and (D) Thyroid cancer

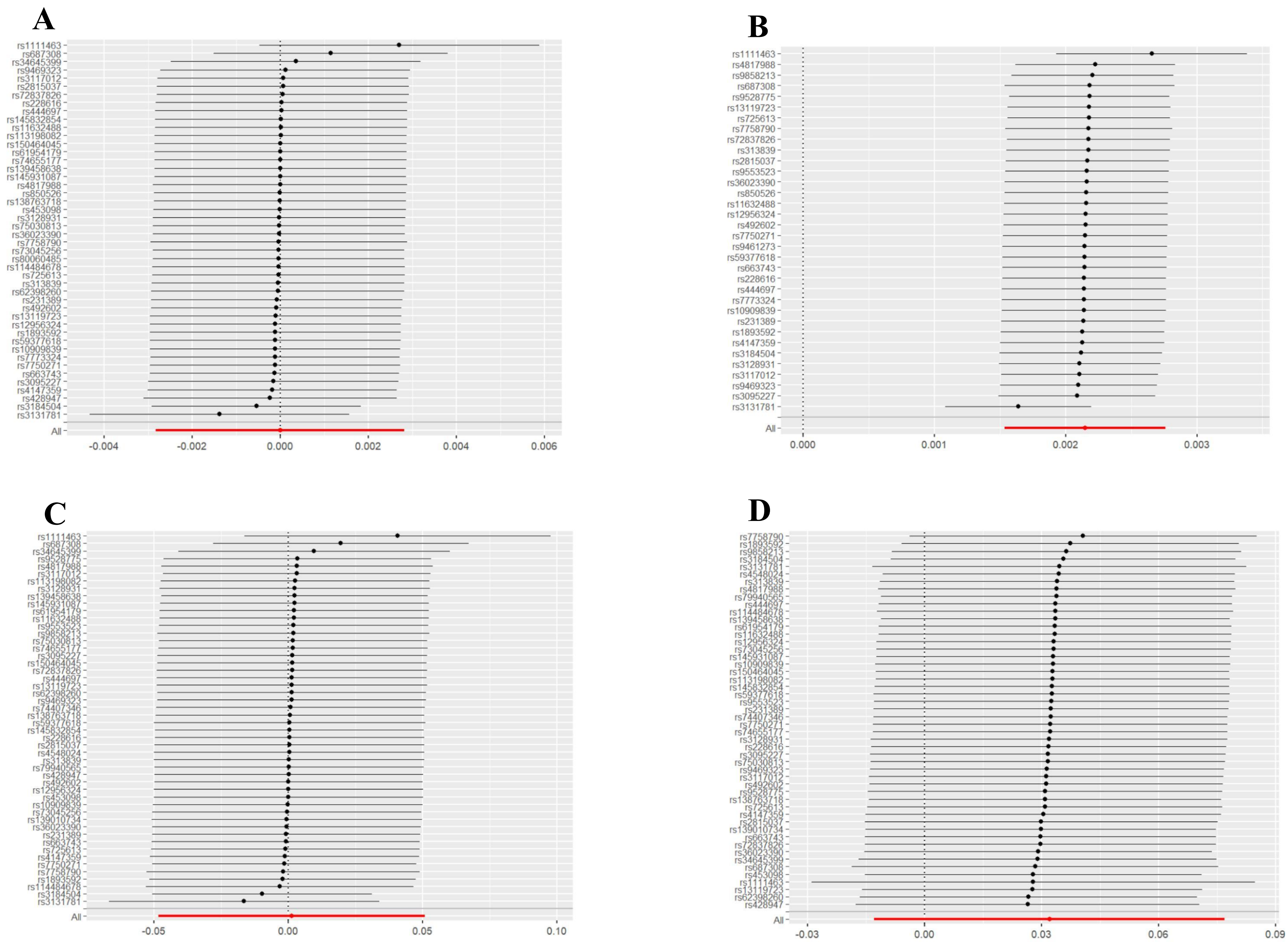

Figure S3. Leave-one-out analysis for the effect of PSC and thyroid diseases. (A) Hypothyroidism, (B) Hyperthyroidism, (C) Hashimoto’thyroiditis and (D) Thyroid cancer

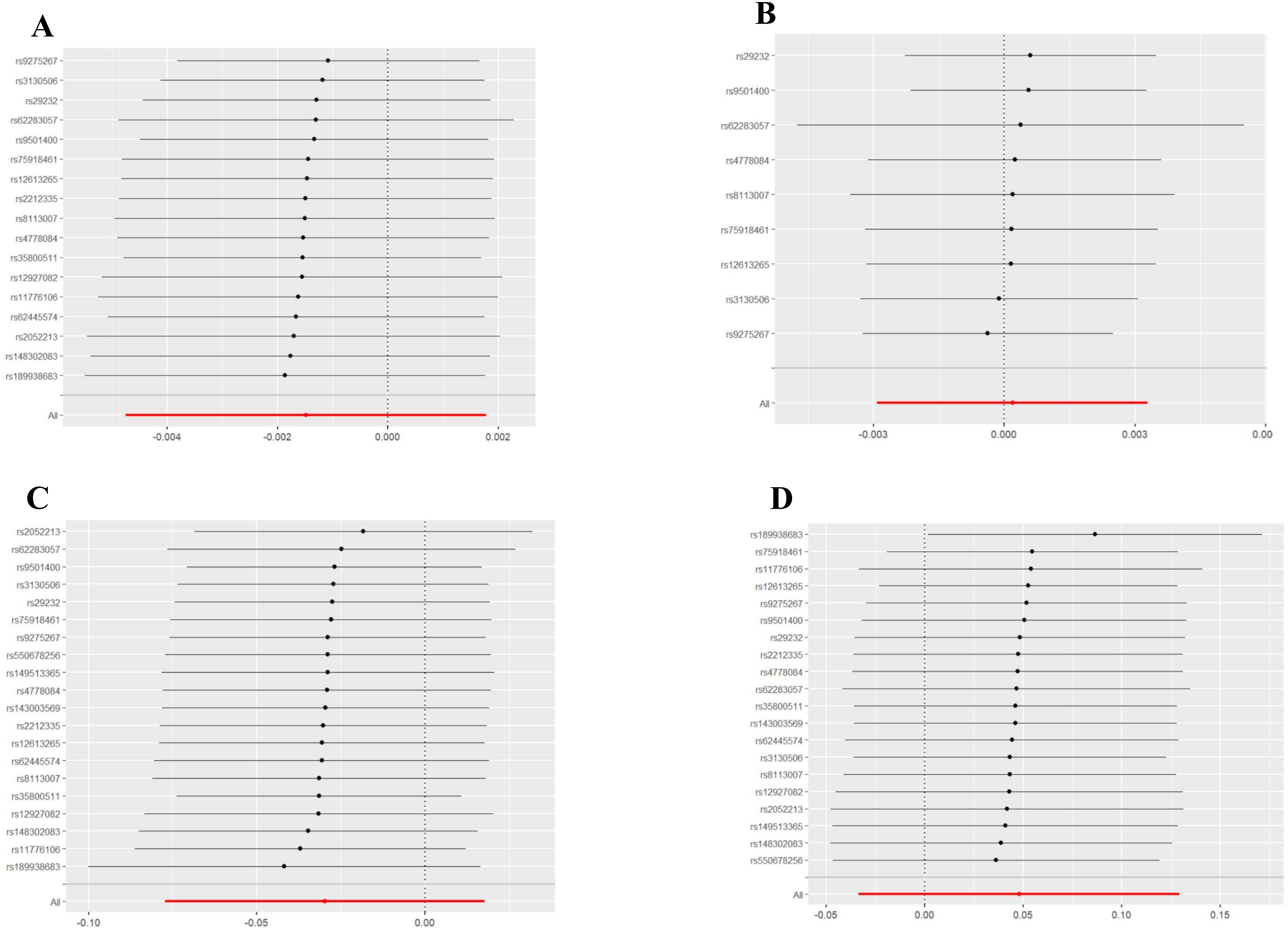

Figure S4. Leave-one-out analysis for the effect of Chronic hepatitis C infection and thyroid diseases. (A) Hypothyroidism, (B) Hyperthyroidism, (C) Hashimoto' thyroiditis and (D) Thyroid cancer

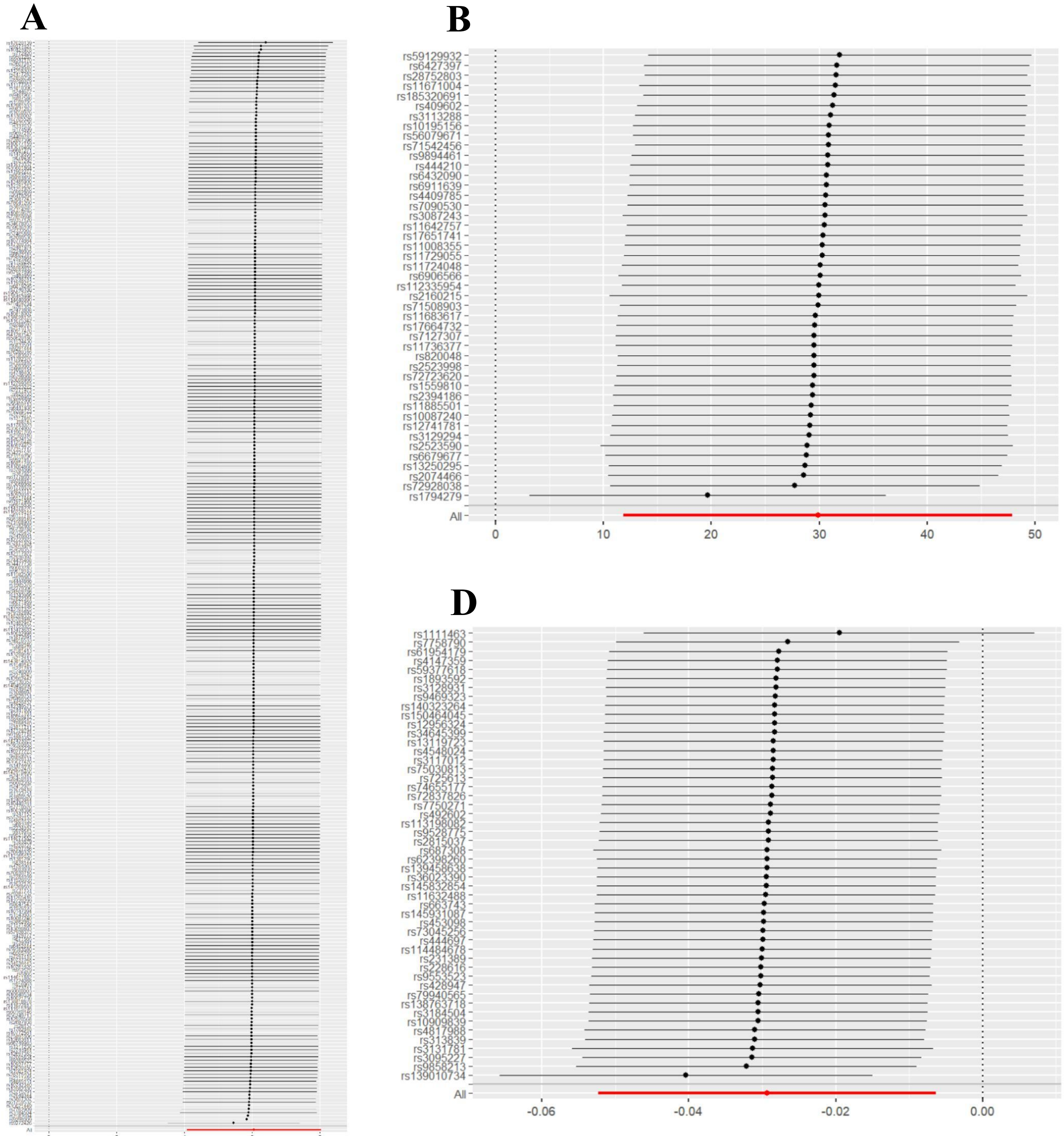

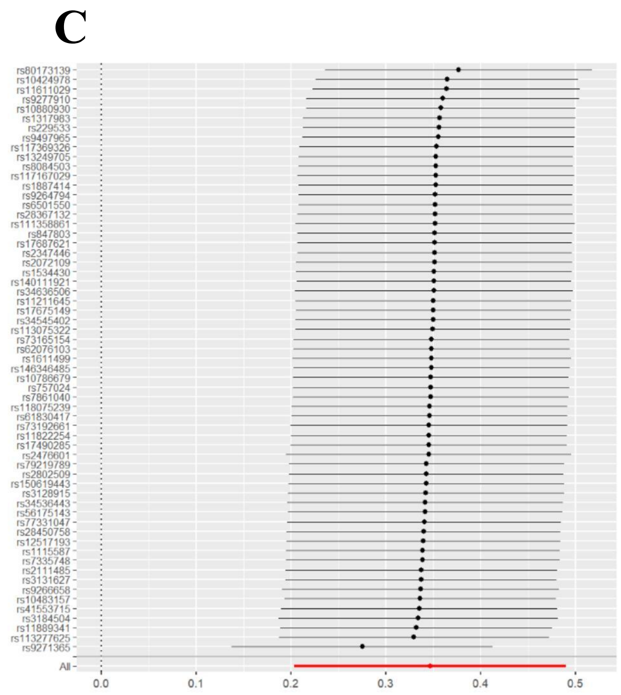

Figure S5. Leave-one-out analysis for the effect of thyroid diseases andAIH. (A) Hypothyroidism, (B) Hyperthyroidism, (C) Hashimoto' thyroiditis and (D) Thyroid cancer

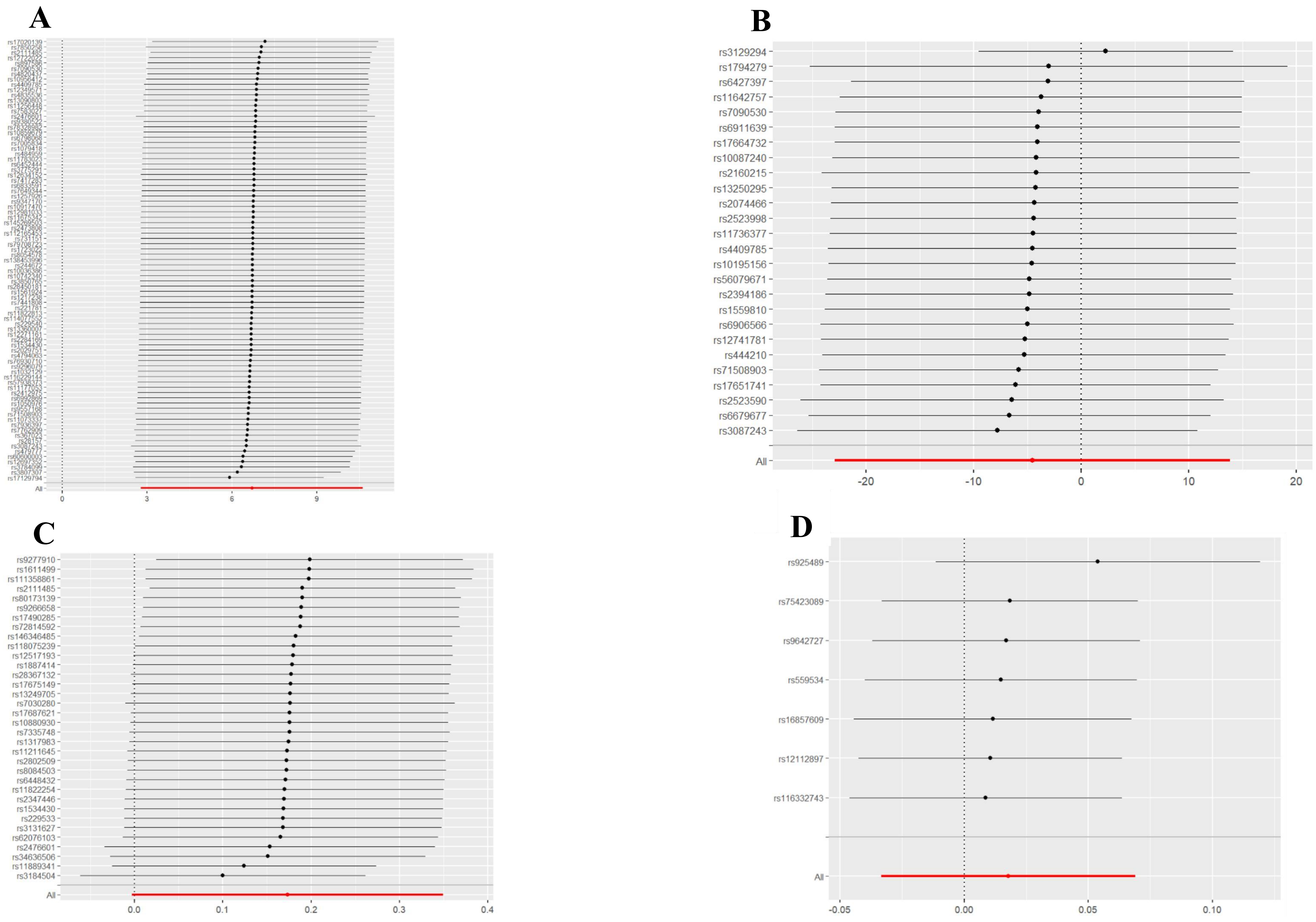

Figure S6. Leave-one-out analysis for the effect of thyroid diseases and PBC. (A) Hypothyroidism, (B) Hyperthyroidism, (C) Hashimoto'thyroiditis and (D) Thyroid cancer

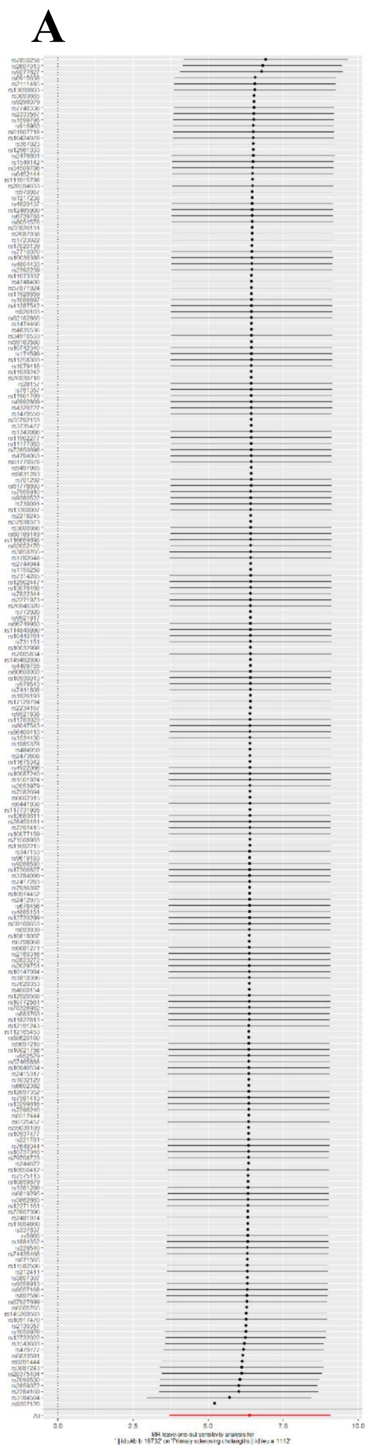

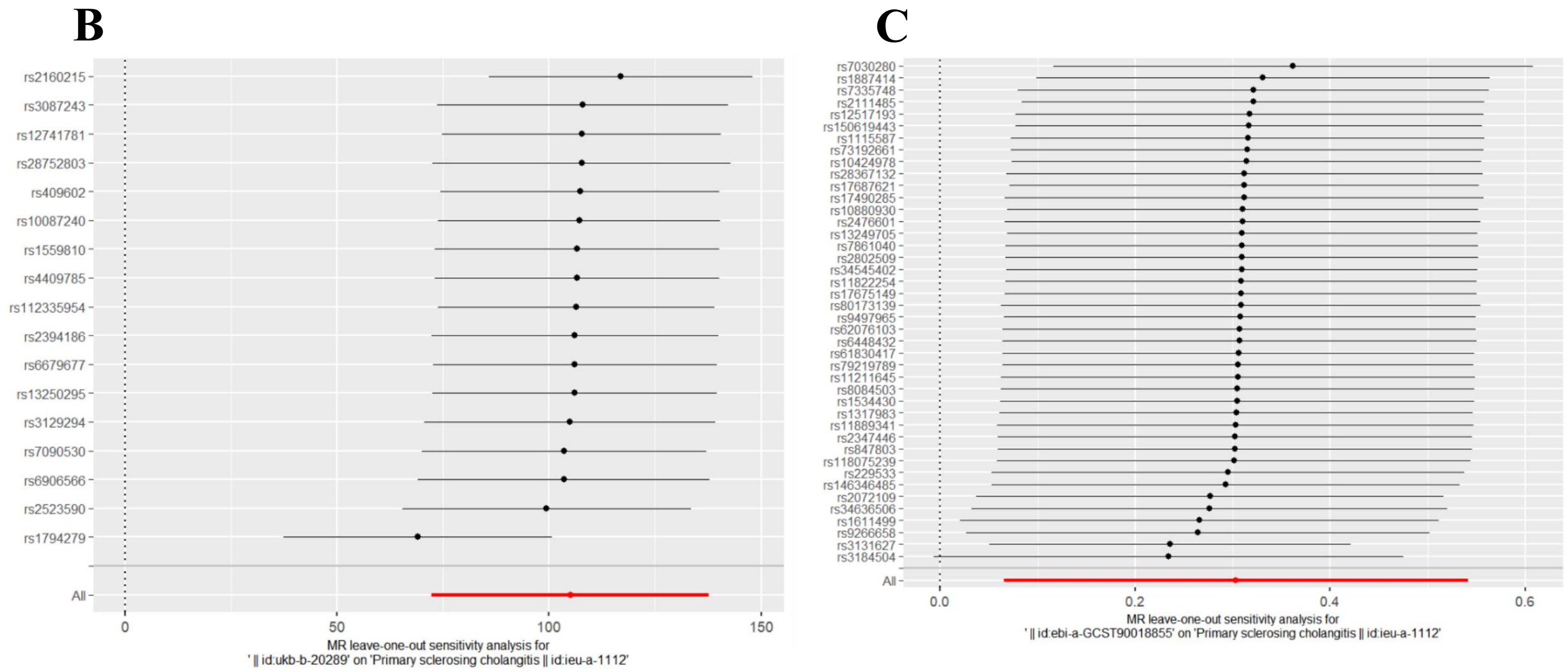

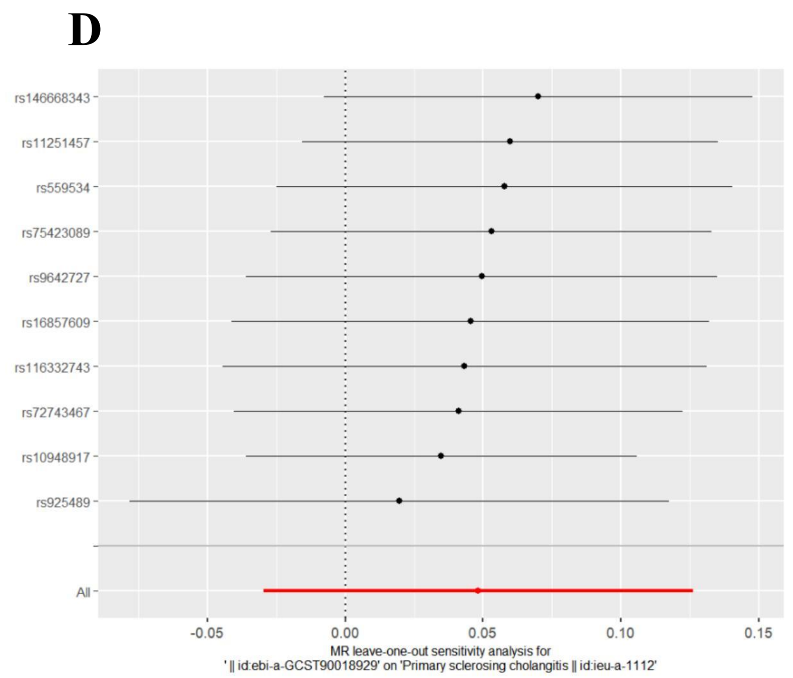

Figure S7. Leave-one-out analysis for the effect of thyroid diseases and PSC. (A) Hypothyroidism, (B) Hyperthyroidism, (C) Hashimoto' thyroiditis and (D) Thyroid cancer

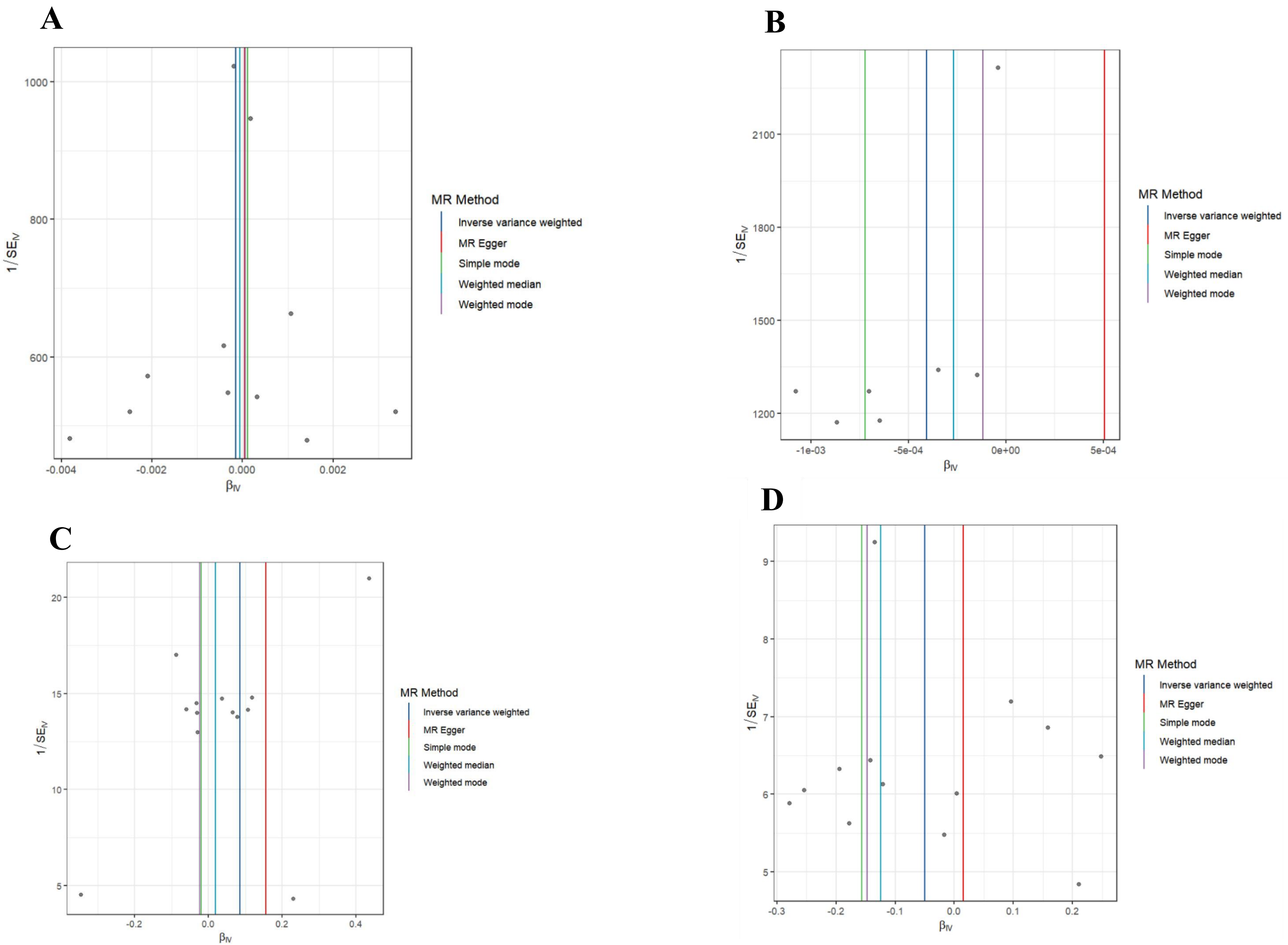

Figure S8. Funnel plot to assess the robustness (AIH and thyroid disease). (A) Hypothyroidism, (B) Hyperthyroidism, (C) Hashimoto' thyroiditis and (D) Thyroid cancer. MR: mendelian randomization; IVW: inverse variance weighted; SE: standard.

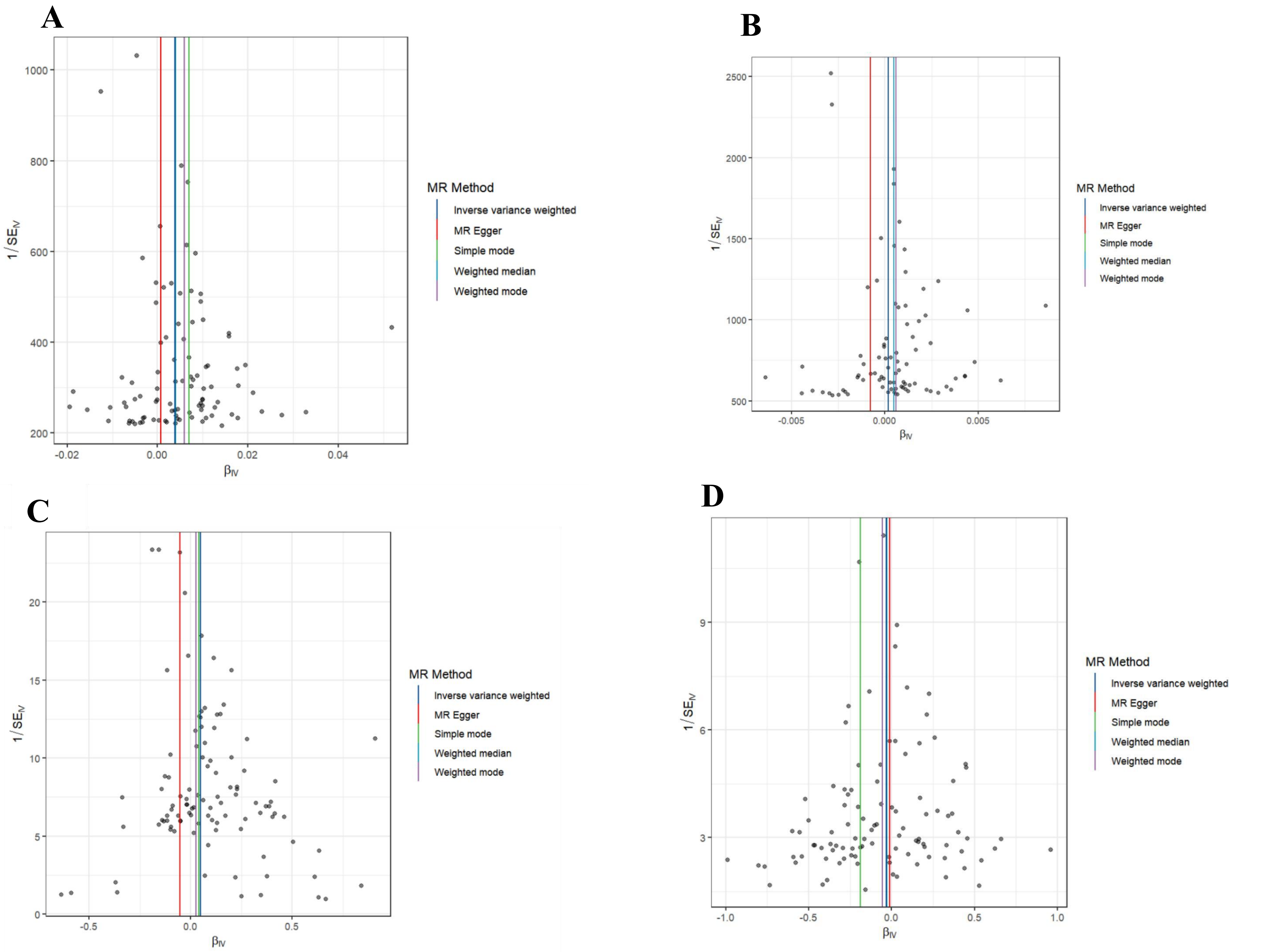

Figure S9. Funnel plot to assess the robustness (PBC and thyroid disease). (A) Hypothyroidism, (B) Hyperthyroidism, (C) Hashimoto' thyroiditis and (D) Thyroid cancer. MR: mendelian randomization; IVW: inverse variance weighted; SE: standard.

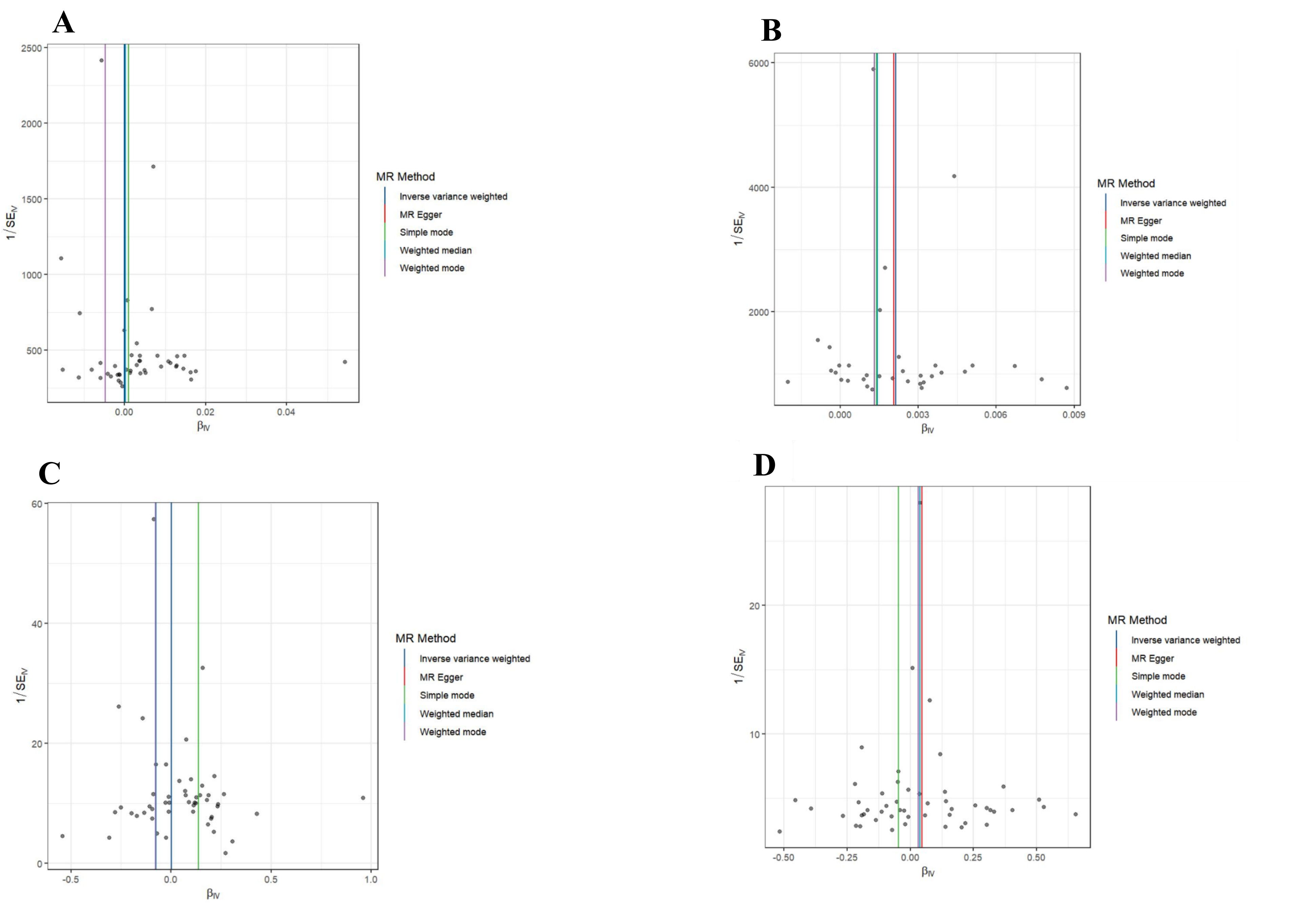

Figure S10. Funnel plot to assess the robustness (PSC and thyroid disease). (A) Hypothyroidism, (B) Hyperthyroidism, (C) Hashimoto' thyroiditis and (D) Thyroid cancer. MR: mendelian randomization; IVW: inverse variance weighted; SE: standard.

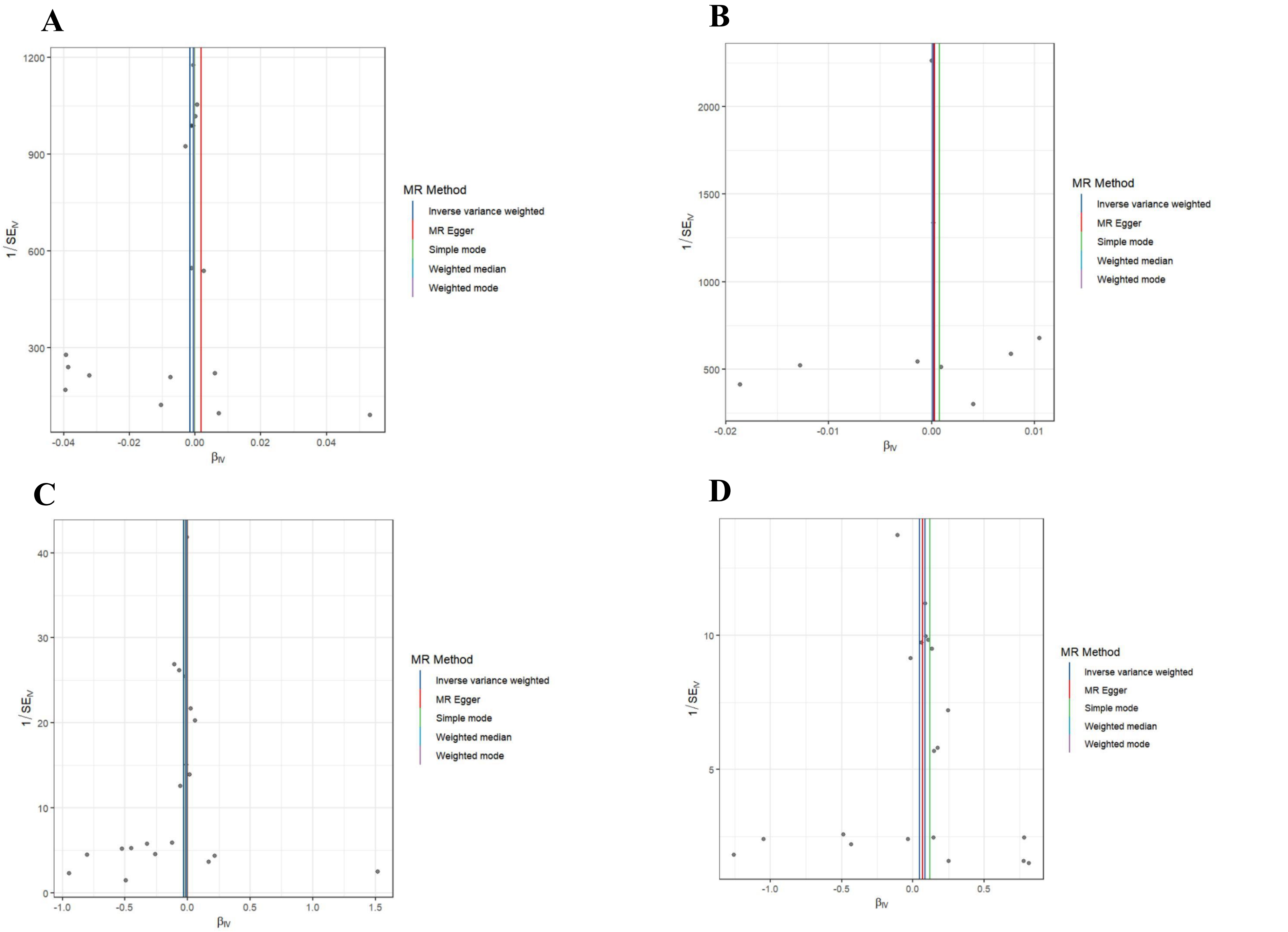

Figure S11. Funnel plot to assess the robustness (Chronic hepatitis C infection and thyroid disease). (A) Hypothyroidism, (B) Hyperthyroidism, (C) Hashimoto' thyroiditis and (D) Thyroid cancer. MR: mendelian randomization; IVW: inverse variance weighted; SE: standard.

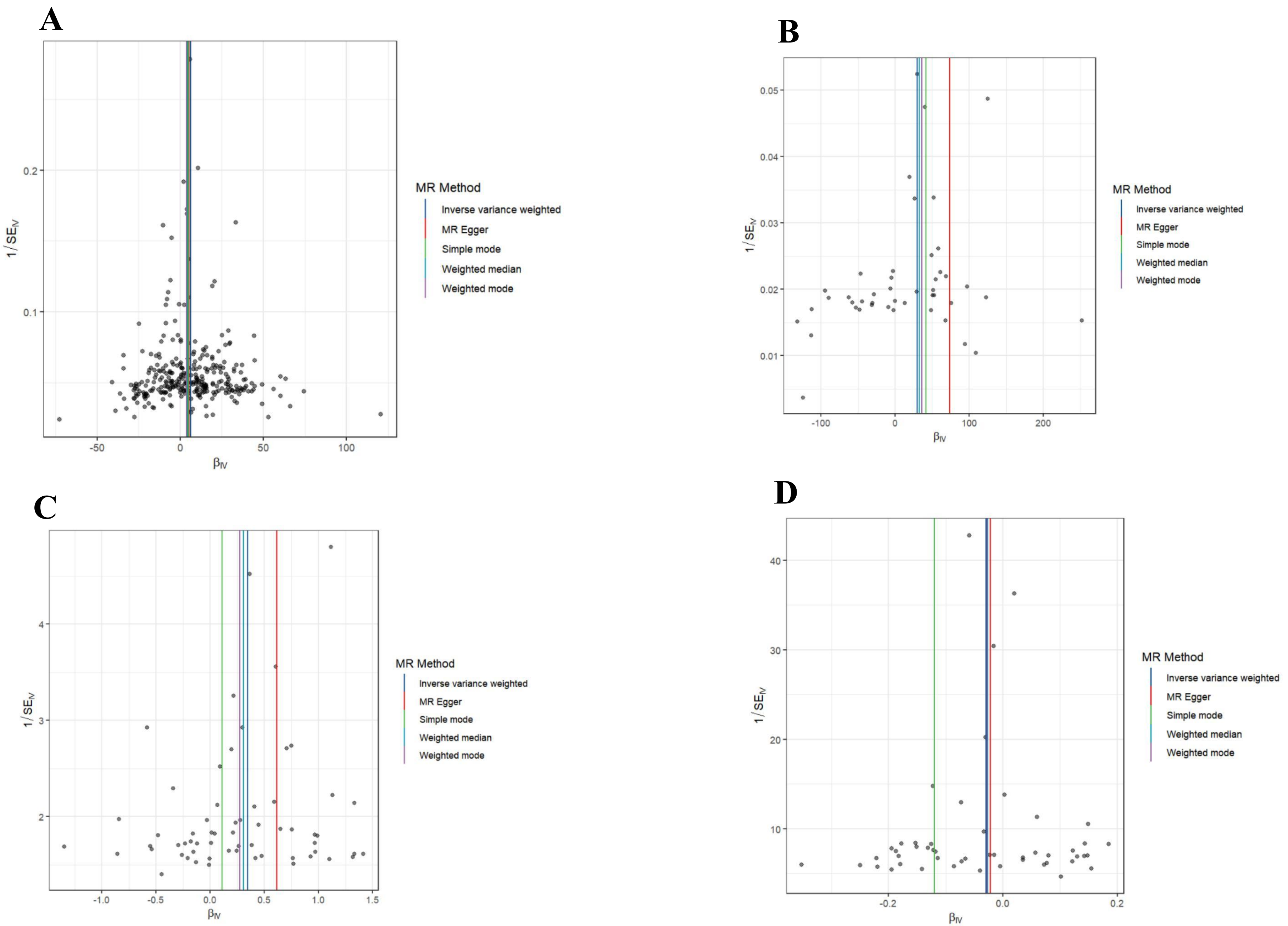

Figure S12. Funnel plot to assess the robustness (thyroid disease and AIH). (A) Hypothyroidism, (B) Hyperthyroidism, (C) Hashimoto' thyroiditis and (D) Thyroid cancer. MR: mendelian randomization; IVW: inverse variance weighted; SE: standard.

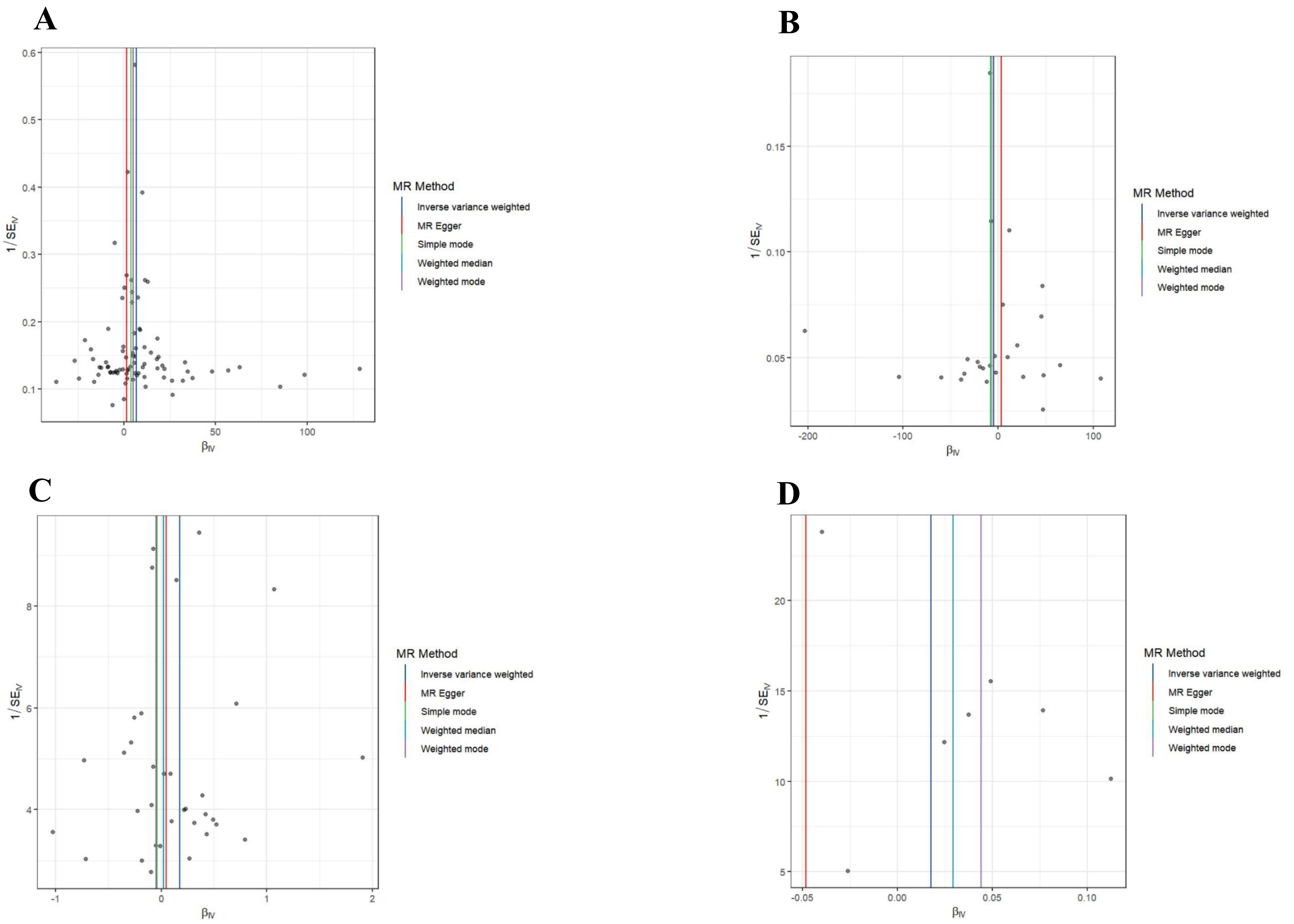

Figure S13. Funnel plot to assess the robustness (thyroid disease and PBC). (A) Hypothyroidism, (B) Hyperthyroidism, (C) Hashimoto'thyroiditis and (D) Thyroid cancer. MR: mendelian randomization; IVW: inverse variance weighted; SE: standard.

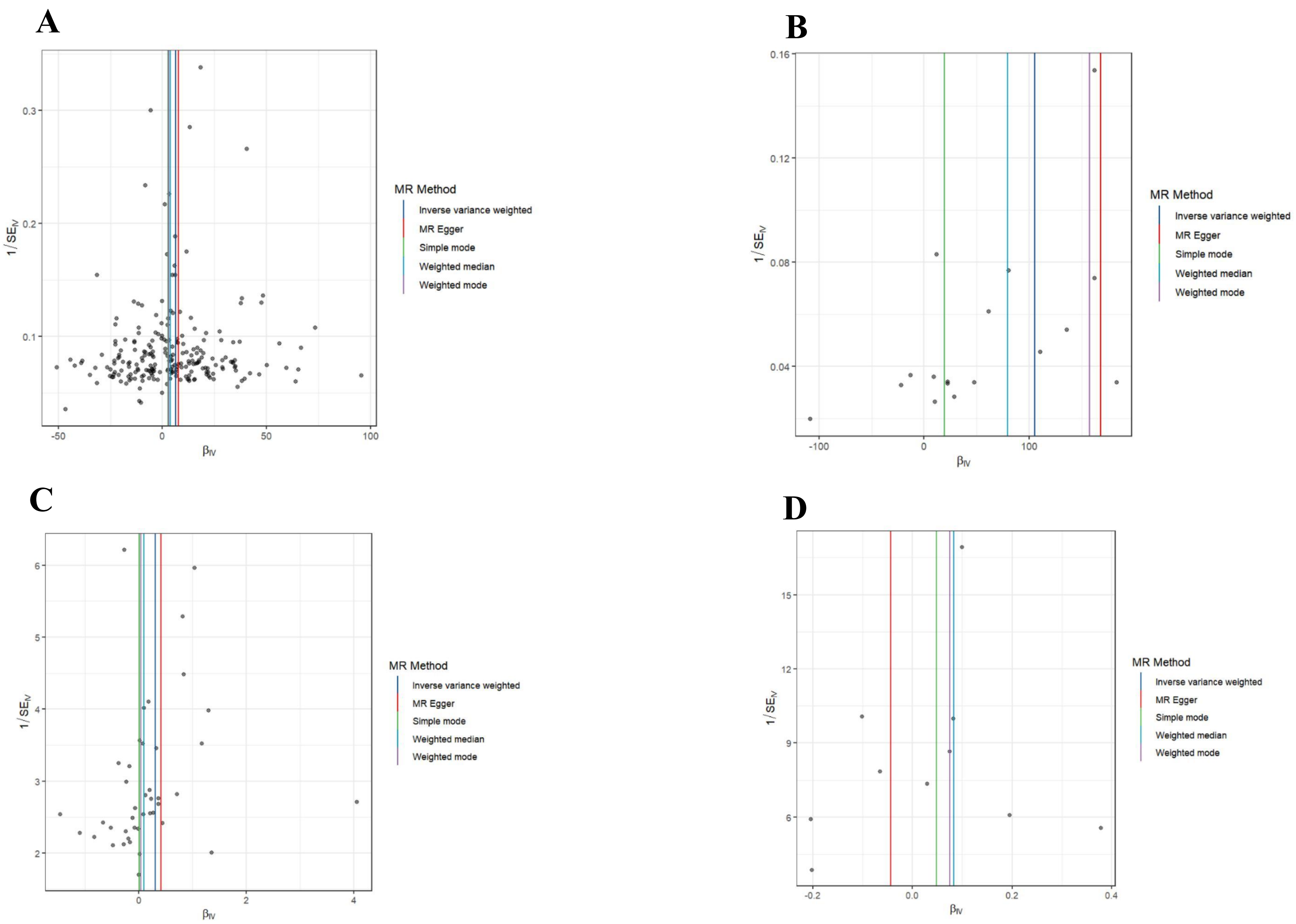

Figure S14. Funnel plot to assess the robustness (thyroid disease and PSC). (A) Hypothyroidism, (B) Hyperthyroidism, (C) Hashimoto' thyroiditis and (D) Thyroid cancer. MR: mendelian randomization; IVW: inverse variance weighted; SE: standard.

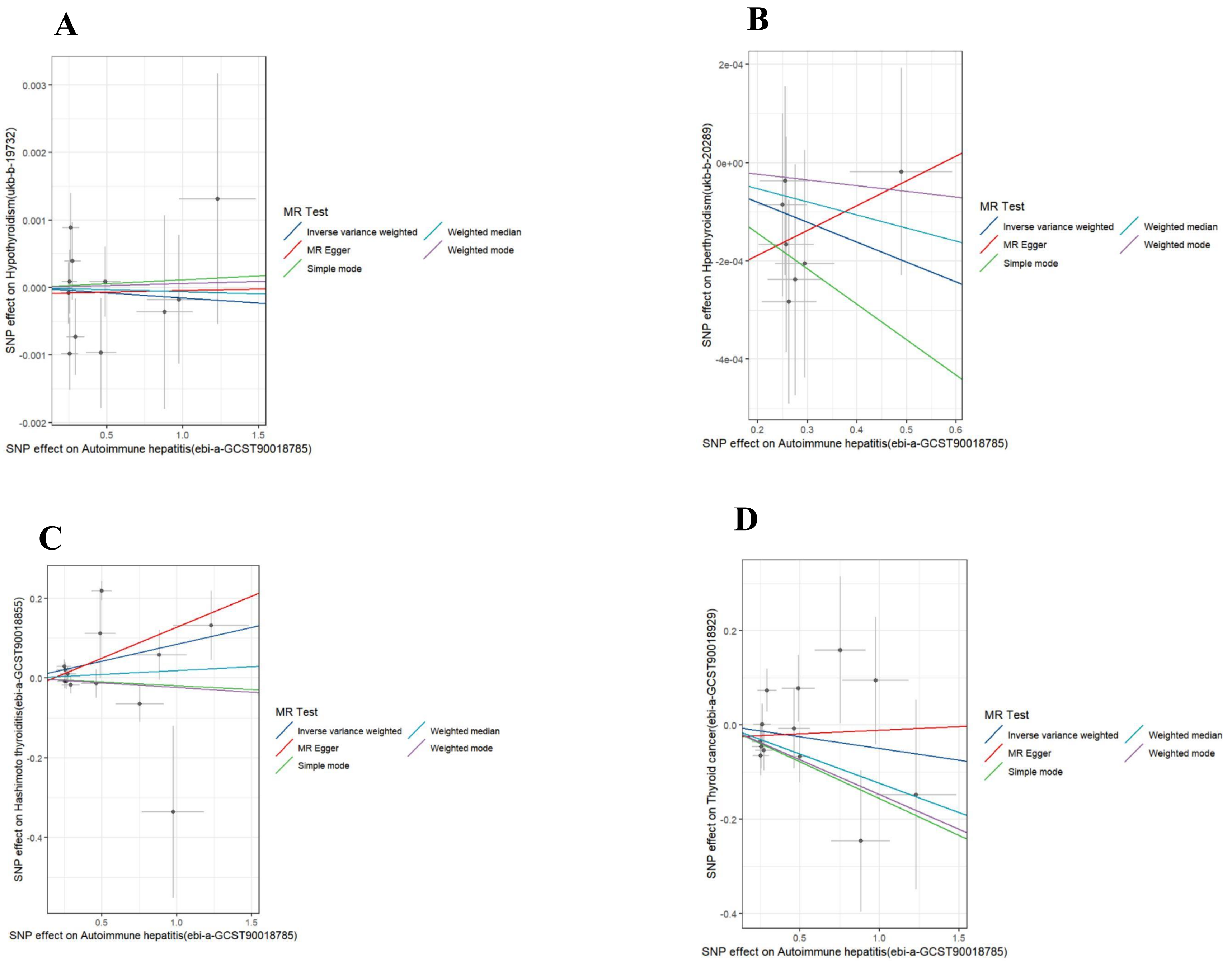

Figure S15. Scatter plot of the association of AIH on thyroid diseases. (A) Hypothyroidism, (B) Hyperthyroidism, (C) Hashimoto' thyroiditis and (D) Thyroid cancer. MR: mendelian randomization.

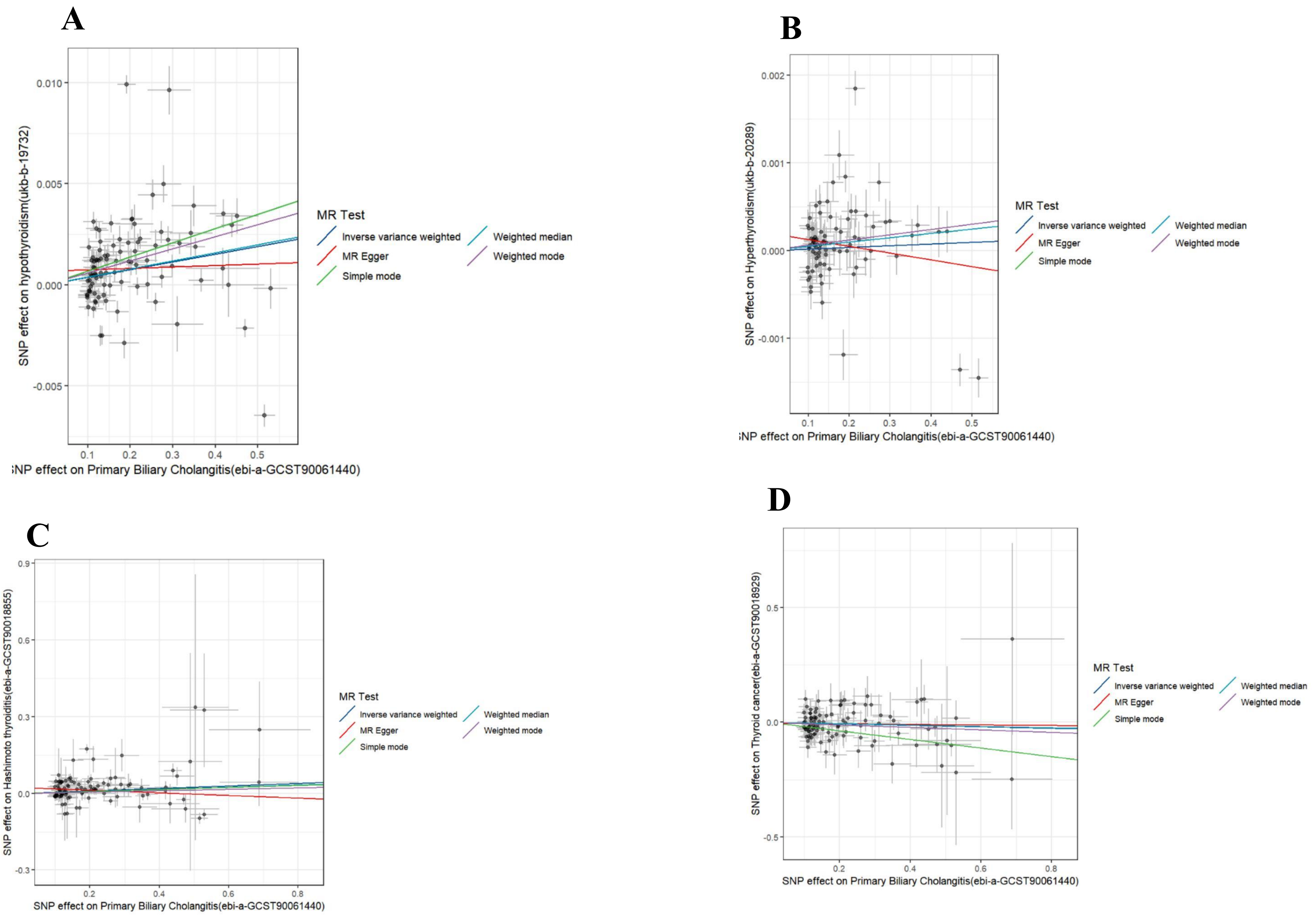

Figure S16. Scatter plot of the association of PBC on thyroid diseases. (A) Hypothyroidism, (B) Hyperthyroidism, (C) Hashimoto' thyroiditis and (D) Thyroid cancer. MR: mendelian randomization.

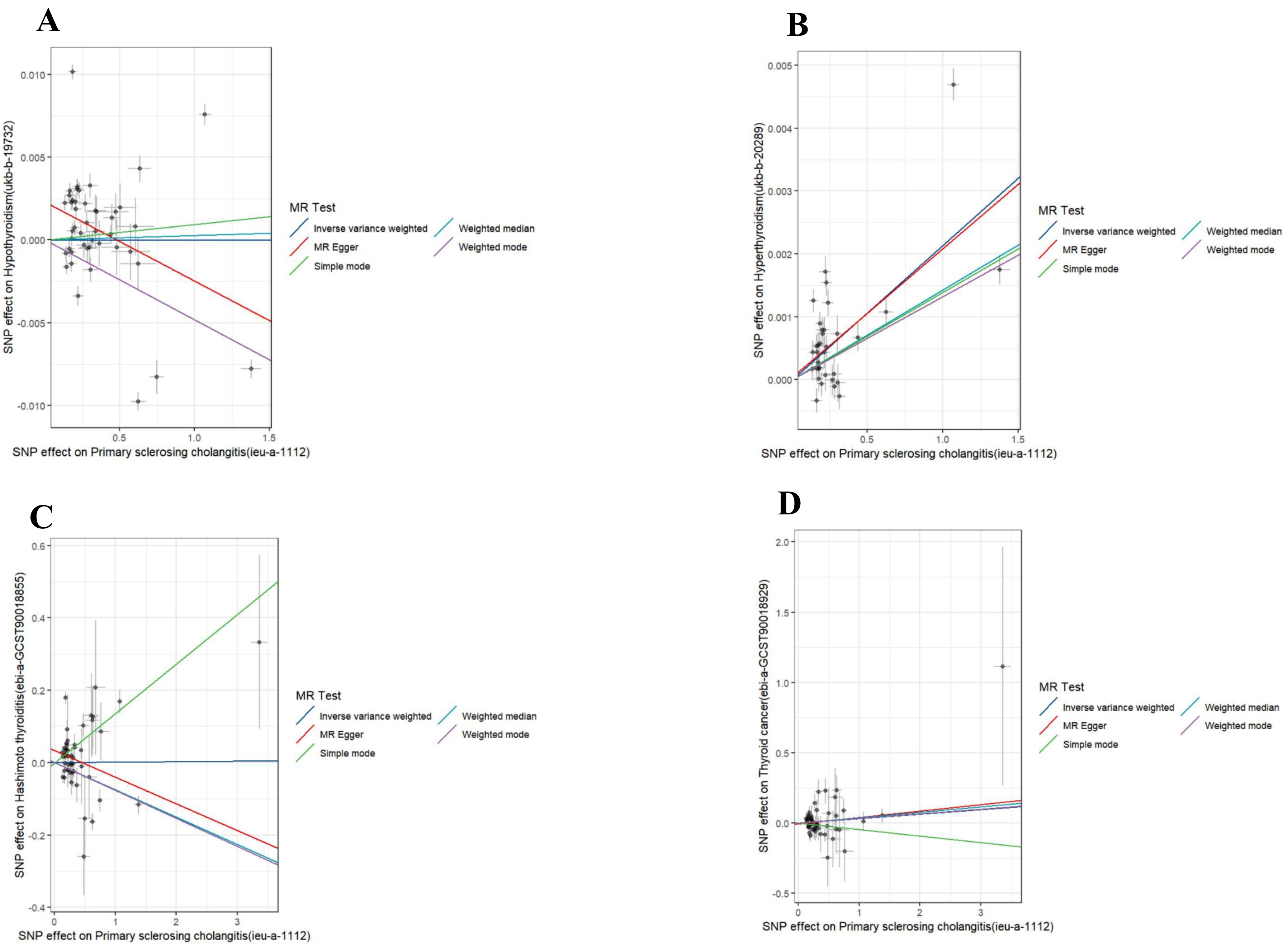

Figure S17. Scatter plot of the association of PSC on thyroid diseases. (A) Hypothyroidism, (B) Hyperthyroidism, (C) Hashimoto' thyroiditis and (D) Thyroid cancer. MR: mendelian randomization.

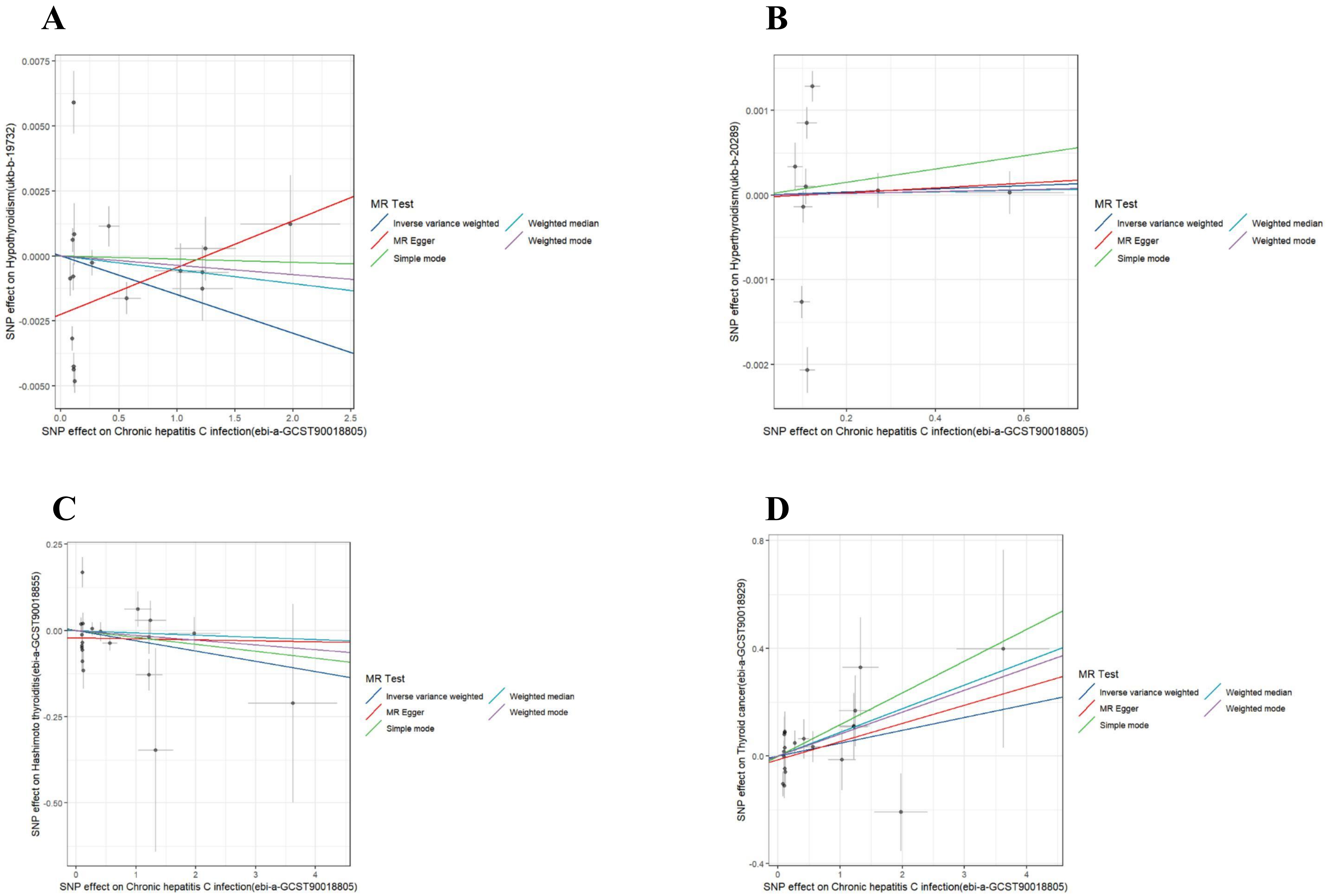

Figure S18. Scatter plot of the association of chronic hepatitis C infection on thyroid diseases. (A) Hypothyroidism, (B) Hyperthyroidism, (C) Hashimoto' thyroiditis and (D) Thyroid cancer. MR: mendelian randomization.

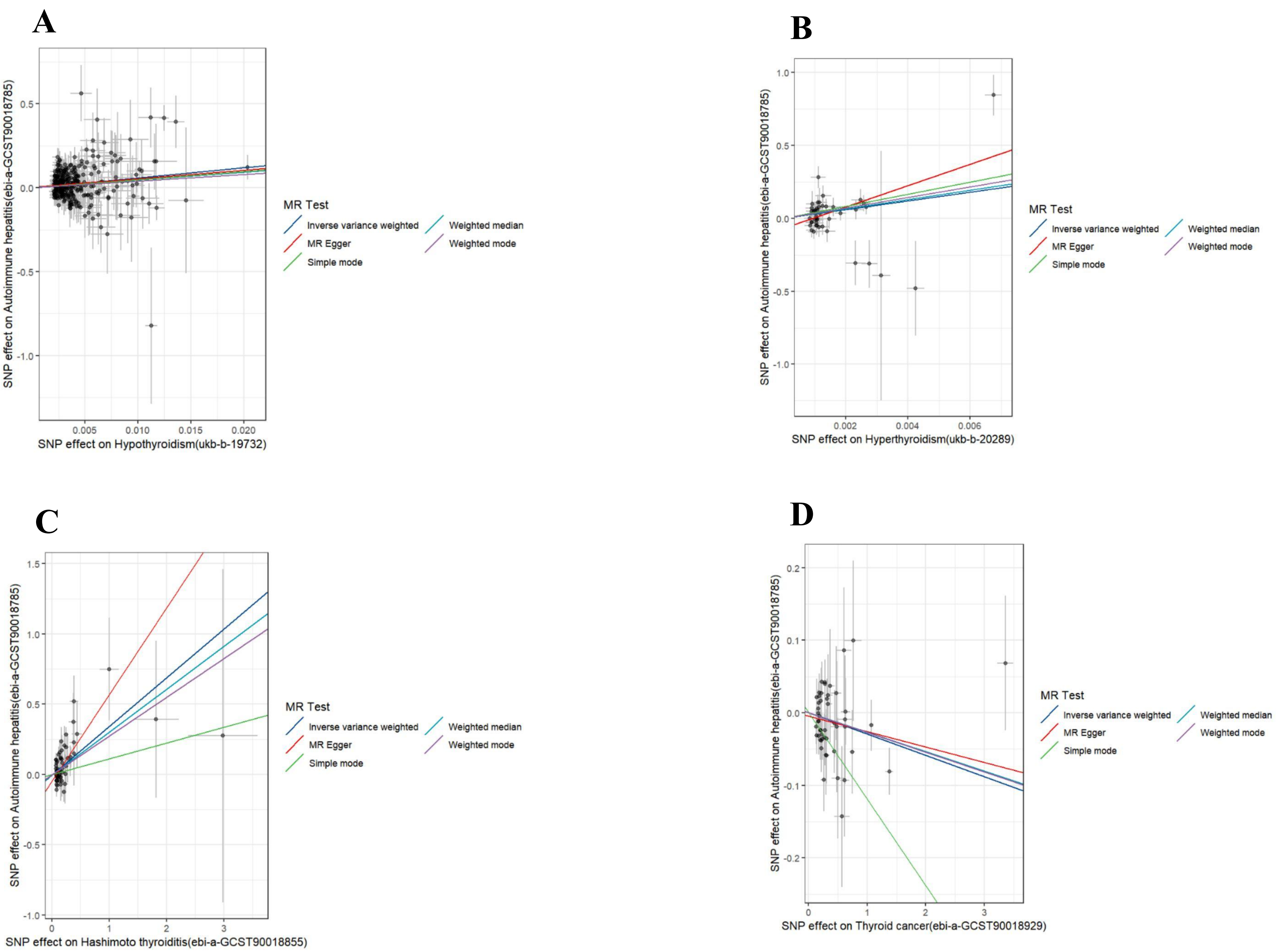

Figure S19. Scatter plot of the association of thyroid diseases on AIH. (A) Hypothyroidism, (B) Hyperthyroidism, (C) Hashimoto' thyroiditis and (D) Thyroid cancer. MR: mendelian randomization.

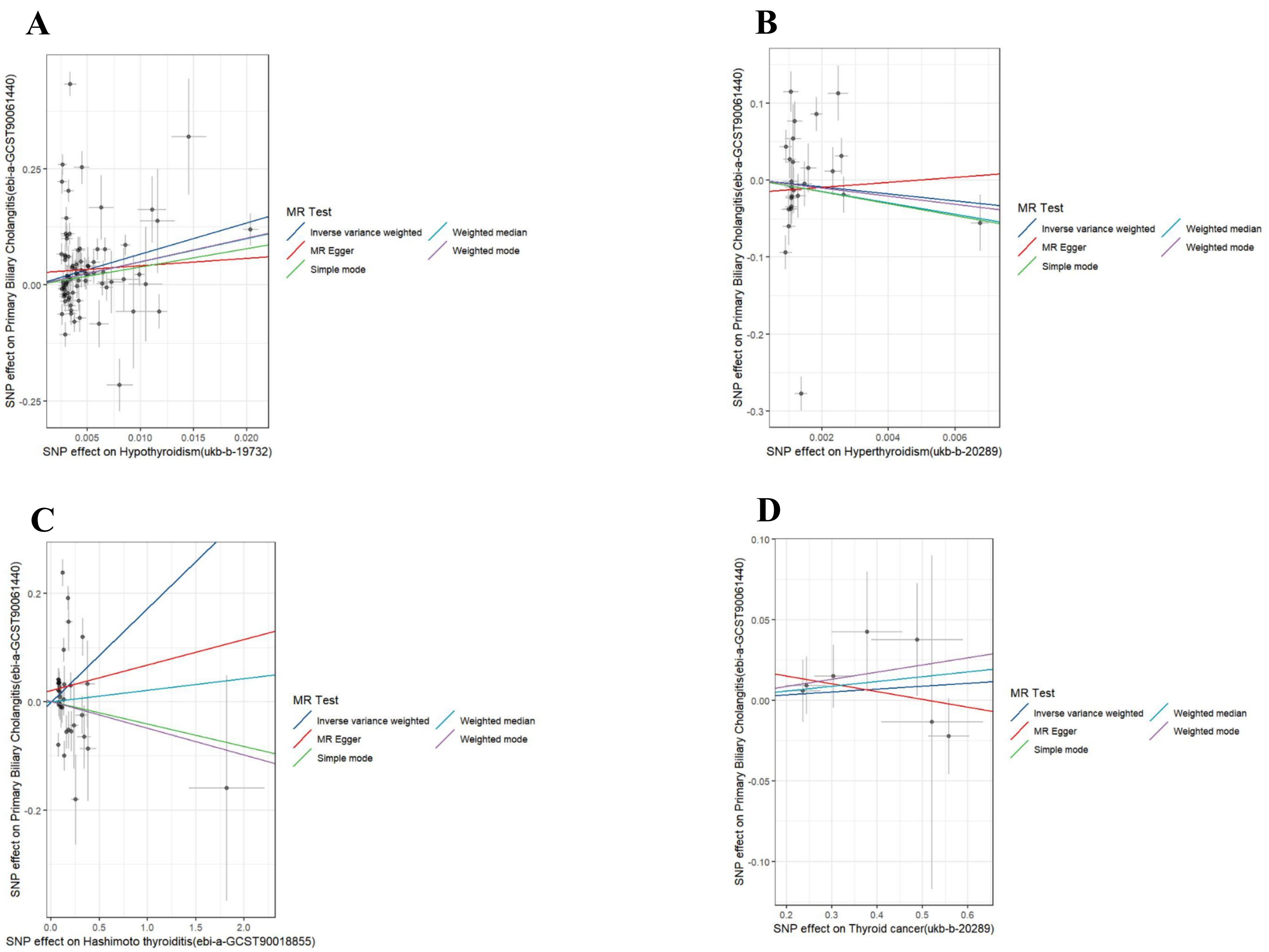

Figure S20. Scatter plot of the association of thyroid diseases on PBC. (A) Hypothyroidism, (B) Hyperthyroidism, (C) Hashimoto' thyroiditis and (D) Thyroid cancer. MR: mendelian randomization.

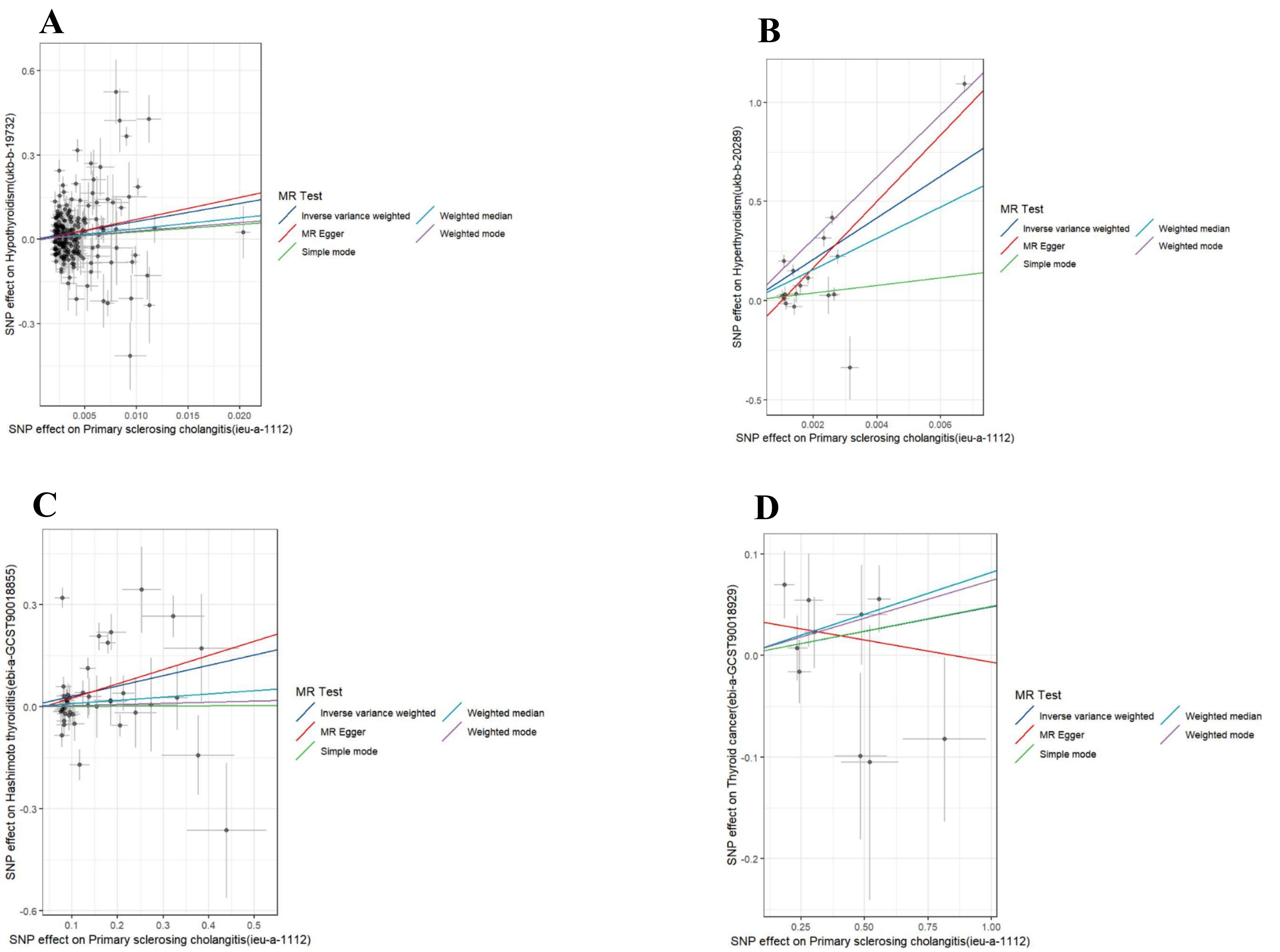

Figure S21. Scatter plot of the association of thyroid diseases on PBC. (A) Hypothyroidism, (B) Hyperthyroidism, (C) Hashimoto’ thyroiditis and (D) Thyroid cancer. MR: mendelian randomization.

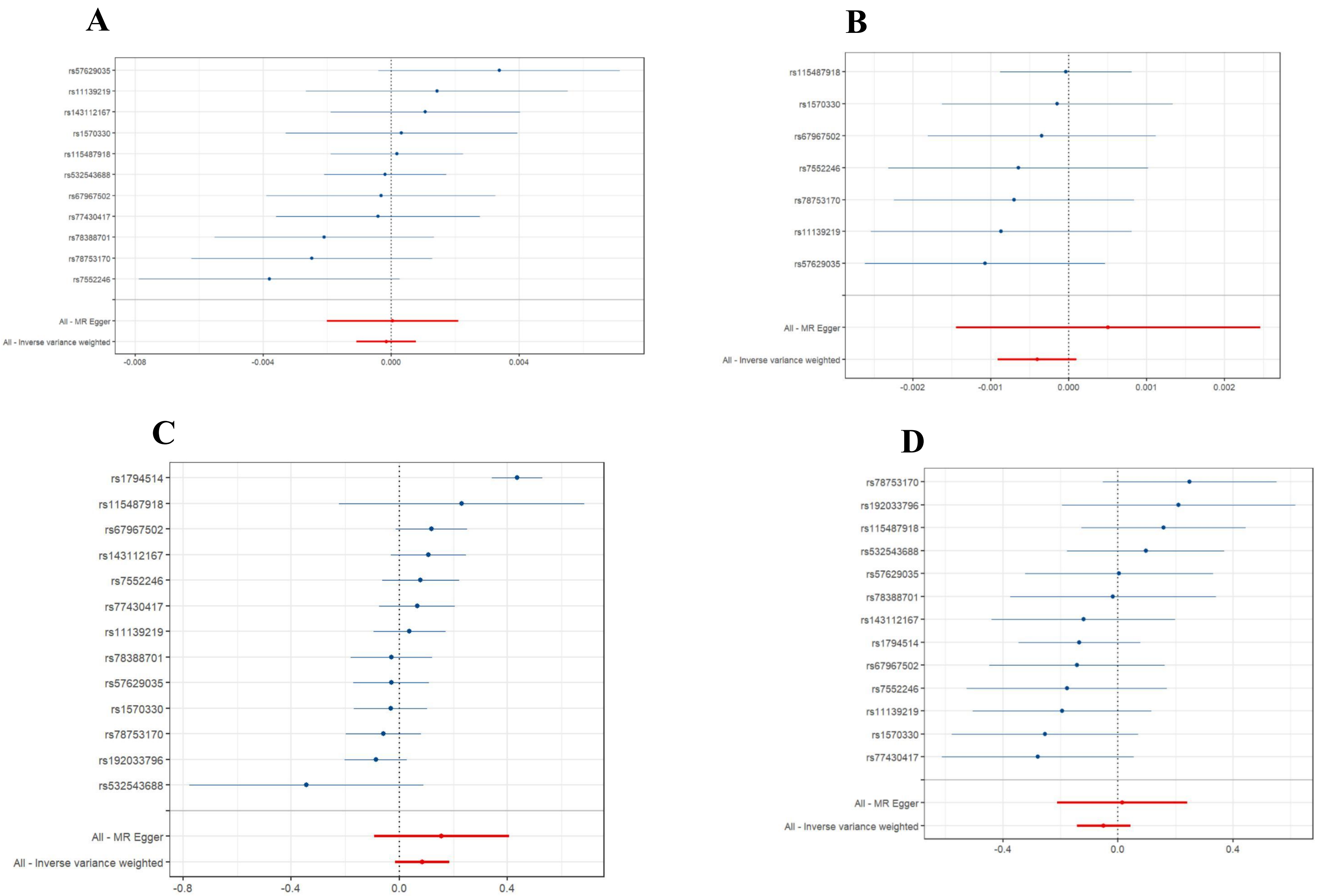

Figure S22. The forest plot of SNPs associated with AIH and their risk on thyroid diseases. (A) Hypothyroidism, (B) Hyperthyroidism, (C) Hashimoto’ thyroiditis and (D) Thyroid cancer. MR: mendelian randomization.

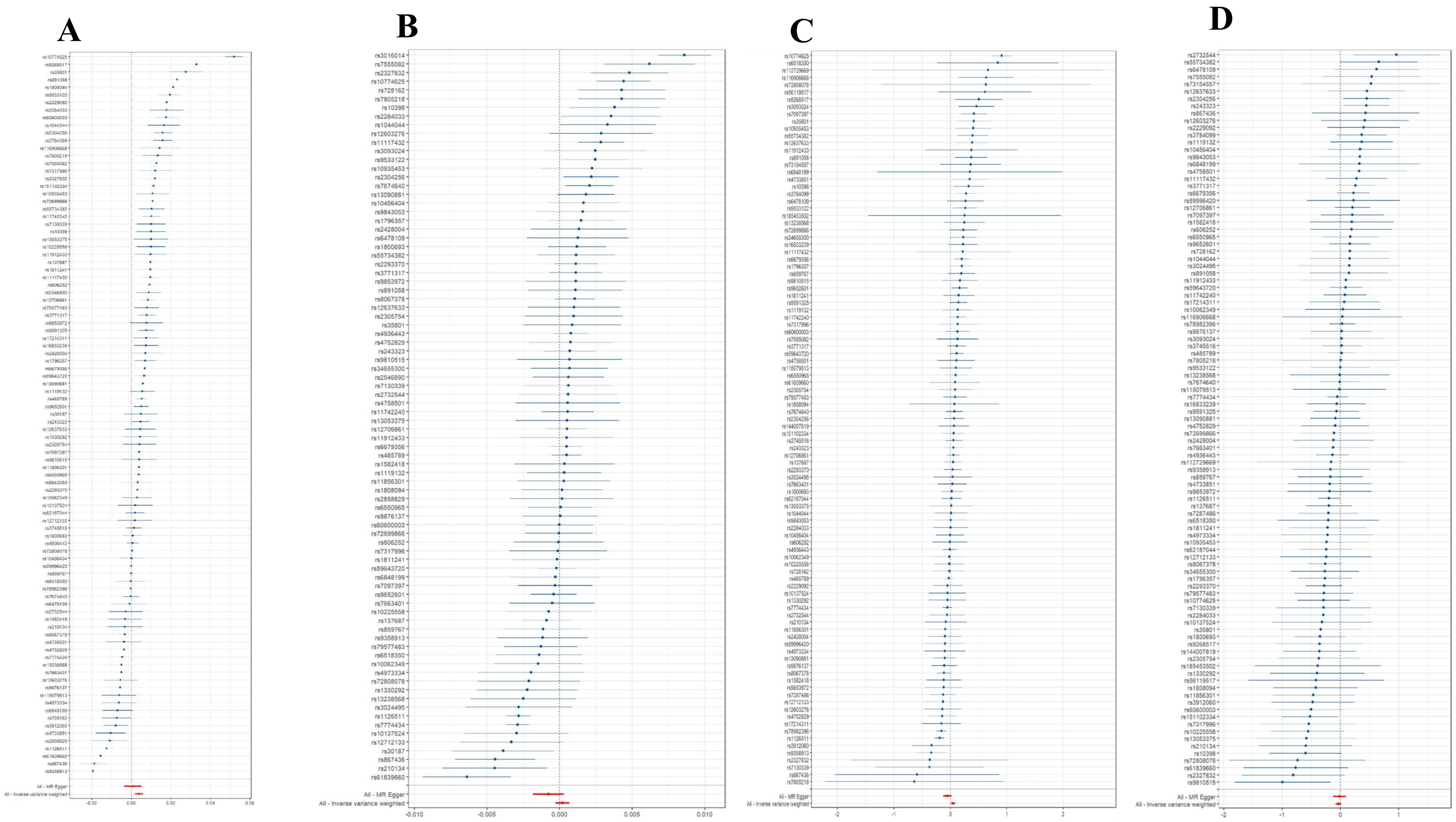

Figure S23. The forest plot of SNPs associated with PBC and their risk on thyroid diseases. (A) Hypothyroidism, (B) Hyperthyroidism, (C) Hashimoto' thyroiditis and (D) Thyroid cancer. MR: mendelian randomization.

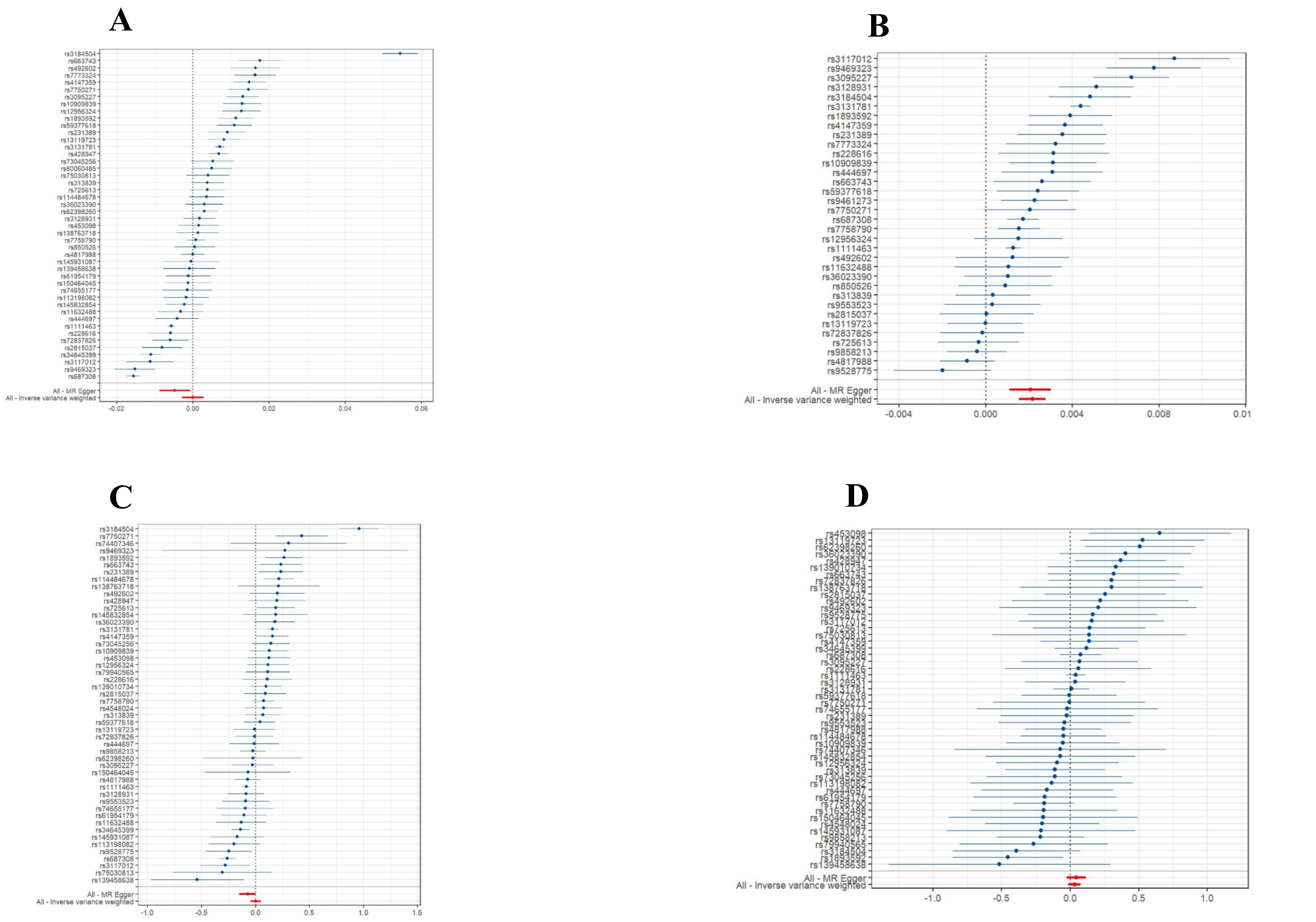

Figure S24. The forest plot of SNPs associated with PSC and their risk on thyroid diseases. (A) Hypothyroidism, (B) Hyperthyroidism, (C) Hashimoto' thyroiditis and (D) Thyroid cancer. MR: mendelian randomization.

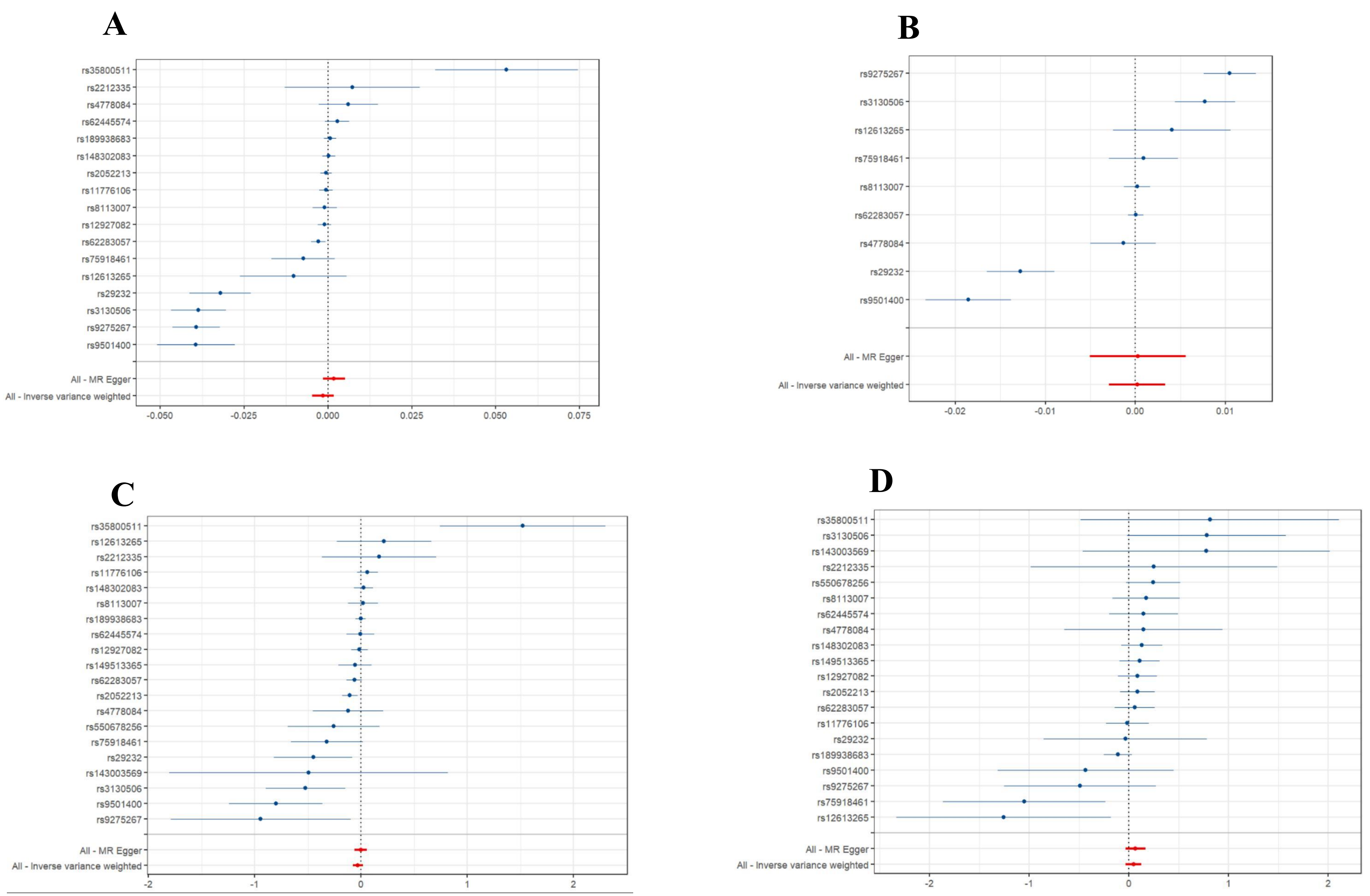

Figure S25. The forest plot of SNPs associated with chronic hepatitis C infection and their risk on thyroid diseases. (A) Hypothyroidism, (B) Hyperthyroidism, (C) Hashimoto’thyroiditis and (D) Thyroid cancer. MR: mendelian randomization.

A

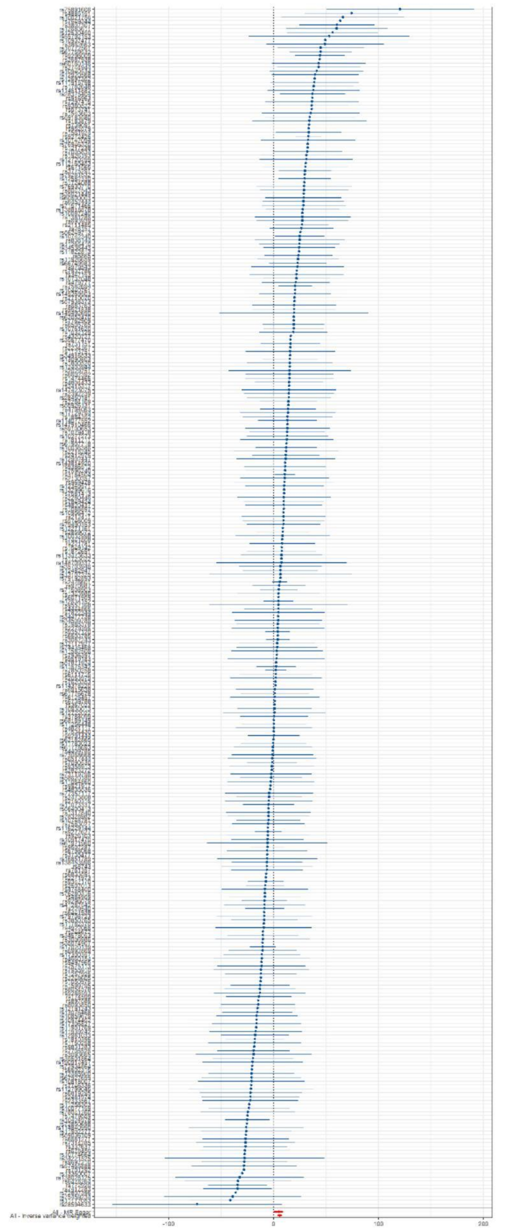

B

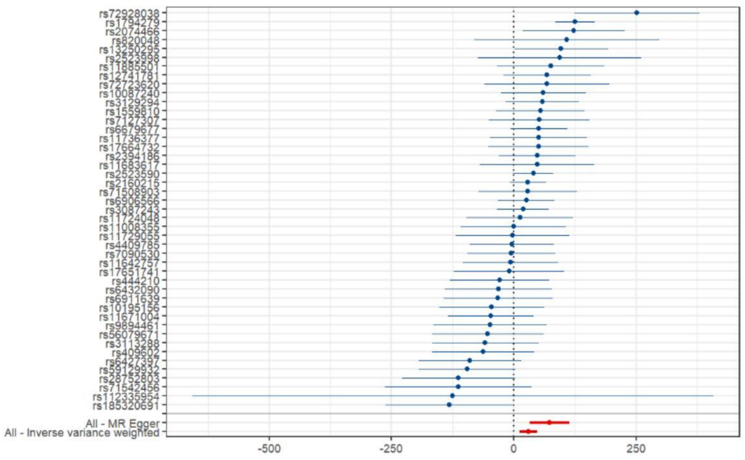

C

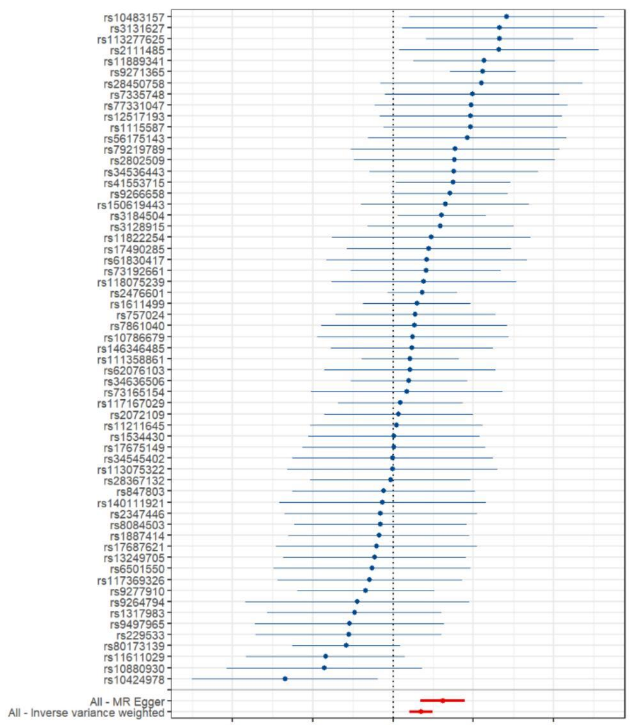

D

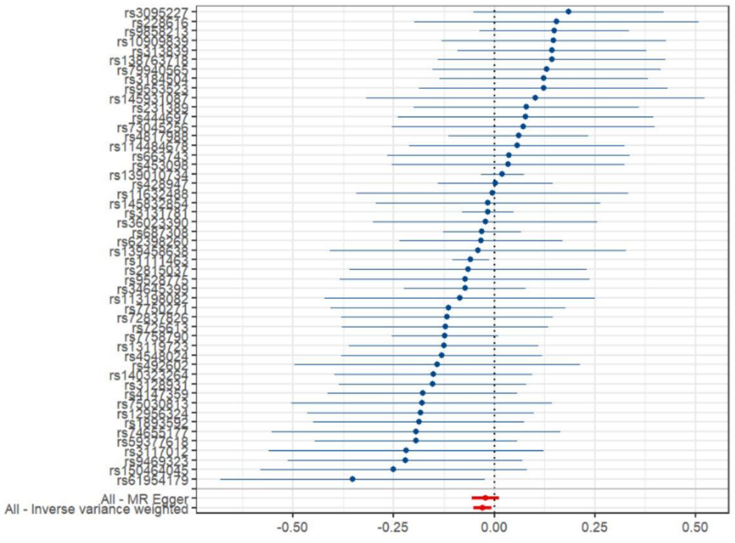

Figure S26. The forest plot of SNPs associated with thyroid diseases and their risk on AIH. (A) Hypothyroidism, (B) Hyperthyroidism, (C) Hashimoto' thyroiditis and (D) Thyroid cancer. MR: mendelian randomization.

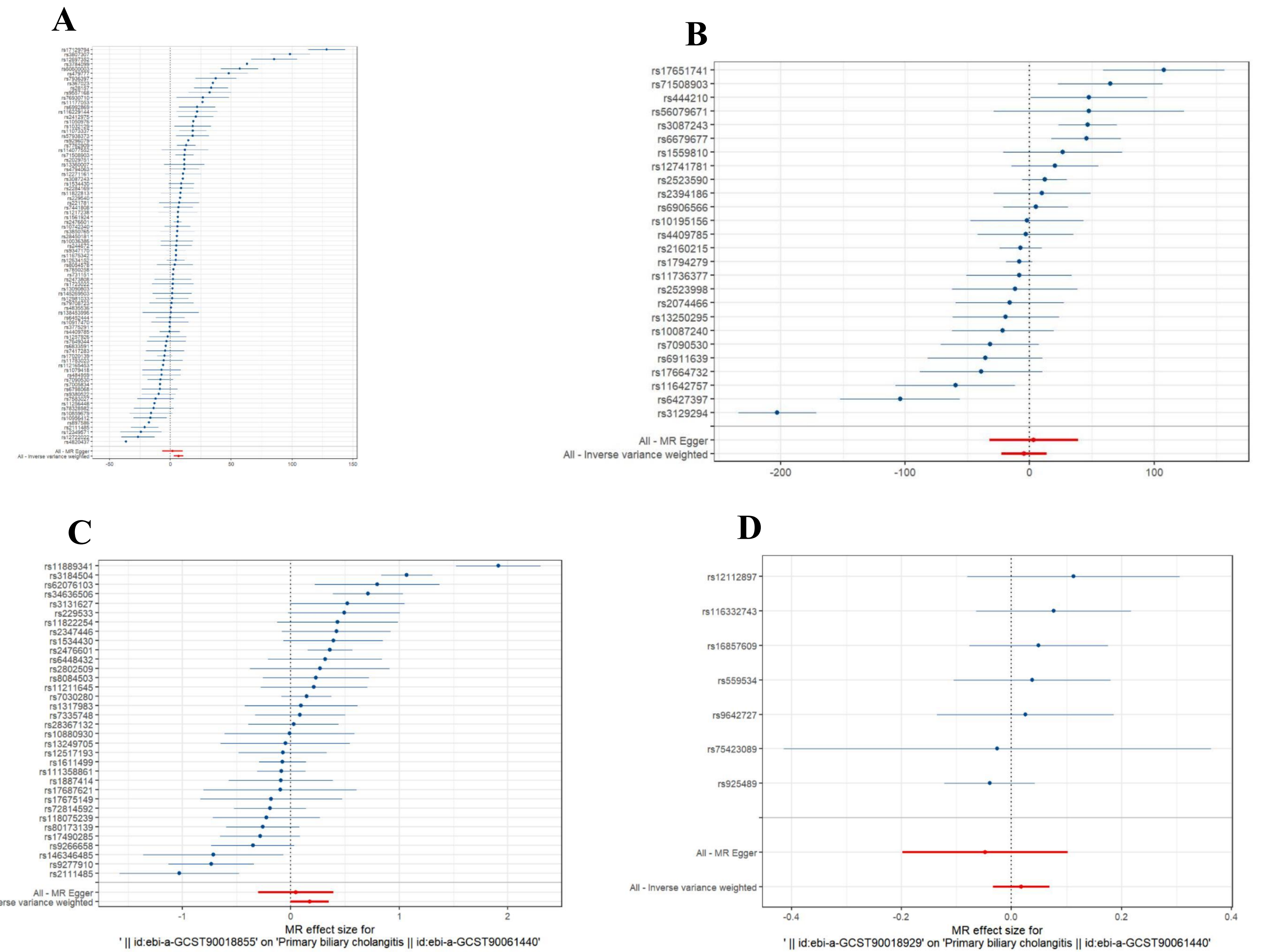

Figure S27. The forest plot of SNPs associated with thyroid diseases and their risk on PBC. (A) Hypothyroidism, (B) Hyperthyroidism, (C) Hashimoto' thyroiditis and (D) Thyroid cancer. MR: mendelian randomization.

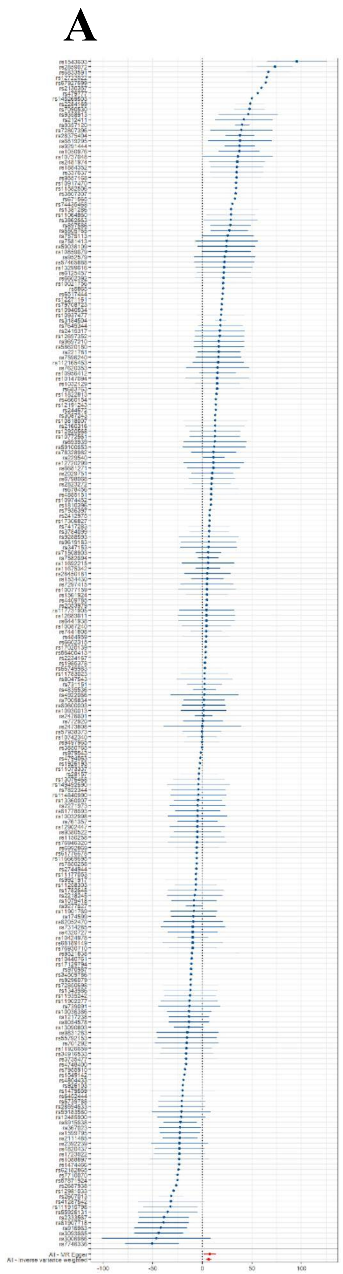

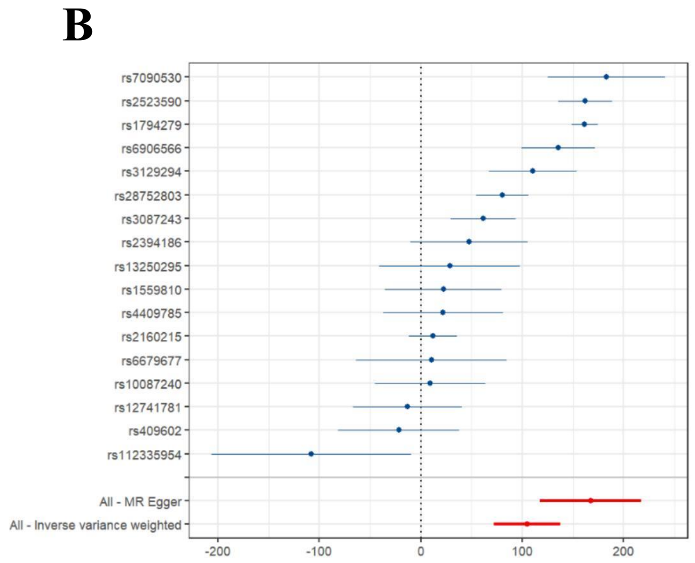

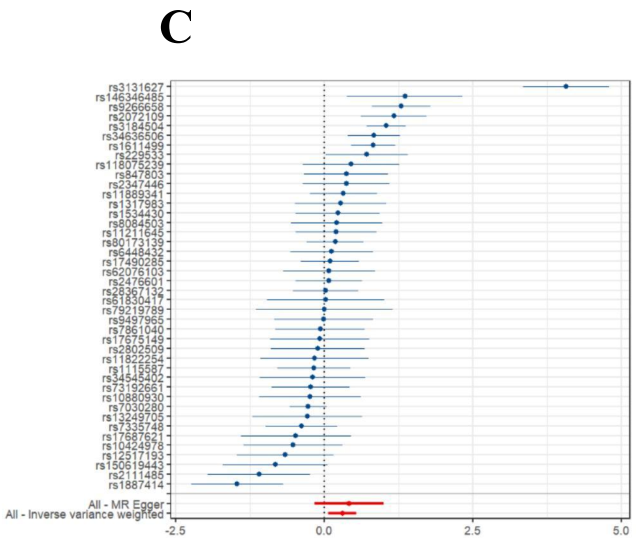

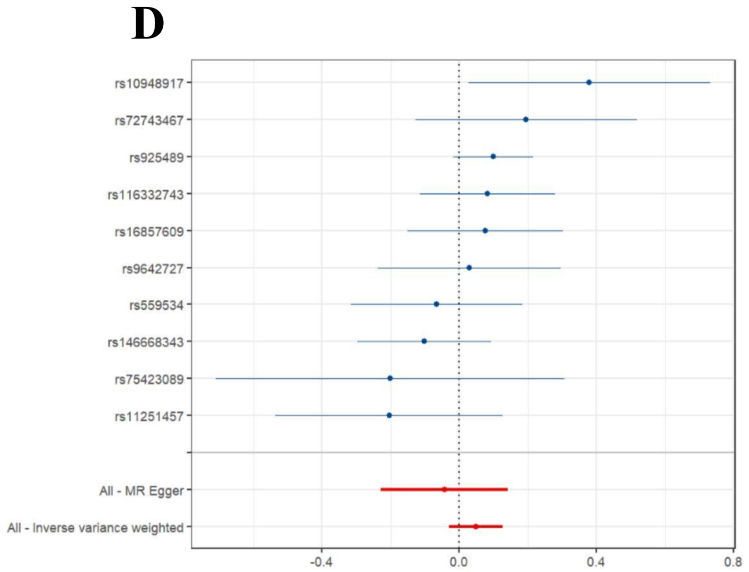

Figure S28. The forest plot of SNPs associated with thyroid diseases and their risk on PSC. (A) Hypothyroidism, (B) Hyperthyroidism, (C) Hashimoto' thyroiditis and (D) Thyroid cancer. MR: mendelian randomization.
